# A Phase II Trial of Defactinib Combined with Avutometinib in Patients with Metastatic Uveal Melanoma

**DOI:** 10.3390/cancers18142232

**Published:** 2026-07-11

**Authors:** Rino S. Seedor, Mizue Terai, Sergei Koshkin, Andrew E. Aplin, Marlana Orloff, Erin Sharpe-Mills, Madeleine Naccarato, Aurora Mills, Alfredo A. Molinolo, J. Silvio Gutkind, Takami Sato

**Affiliations:** 1 Department of Medical Oncology, Sidney Kimmel Comprehensive Cancer Center, Thomas Jefferson University, Philadelphia, PA 19107, USA; mizue.terai@jefferson.edu (M.T.); sergei.koshkin@jefferson.edu (S.K.); marlana.orloff@jefferson.edu (M.O.); erin.sharpe-mills@jefferson.edu (E.S.-M.); madeleine.naccarato@jefferson.edu (M.N.); takami.sato@jefferson.edu (T.S.); 2 Department of Pharmacology, Physiology and Cancer Biology, Thomas Jefferson University, Philadelphia, PA 19107, USA; andrew.aplin@jefferson.edu; 3 Moores Cancer Center, University of California San Diego, San Diego, CA 92093, USA; amolinolo@health.ucsd.edu (A.A.M.); sgutkind@health.ucsd.edu (J.S.G.)

**Keywords:** uveal melanoma, metastatic uveal melanoma, targeted therapy, FAK inhibitor, defactinib, avutometinib

## Abstract

**Background:** Treatment options are limited, and outcomes remain poor for patients with metastatic uveal melanoma (MUM). We conducted an investigator-initiated, prospective, single-arm, single-institution, phase II study evaluating the combination of an FAK inhibitor (defactinib) with a RAF/MEK inhibitor (avutometinib) for the treatment of MUM. **Methods:** From February 2021 through January 2023, 12 patients with MUM were treated with the combination of defactinib and avutometinib. Defactinib was given 200 mg twice daily, and avutometinib was given 3.2 mg twice a week. Both drugs were given for 3 weeks on and 1 week off (28-day cycle). Disease control rate was the primary endpoint of this study. **Results:** Median lines of prior therapies for the patients were two. After two cycles, six patients achieved stable disease while six patients developed progressive disease (disease control rate of 50%). With a median follow-up of 20.0 months, the median progression-free survival was 3.0 months and the median overall survival was 20.0 months. The combination was quite tolerable for patients, with no patients requiring dose reduction or discontinuation. Trial enrollment was stopped early by study sponsors before the anticipated accrual of 18 patients due to no patients having a significant reduction in disease. **Conclusions:** This is the first study reporting on the use of an FAK inhibitor combination in MUM. Further research should seek to elucidate an optimal combination treatment strategy for MUM, such as FAK and PKC inhibitors, for improved blockade of the signaling pathways downstream of GNAQ/GNA11 driver mutations.

## Simple Summary

Although significant improvements in survival have been achieved in cutaneous melanoma, metastatic uveal melanoma (MUM) remains a deadly disease with limited treatment options. This study is a phase II study evaluating the combination of an FAK inhibitor (defactinib) with a RAF/MEK inhibitor (avutometinib) for the treatment of MUM. Twelve patients with a median of two prior therapies were treated in the study, with a disease control rate of 50%. Patients who achieved stabilization of their disease were on treatment for an average of 5.6 months. The combination was quite tolerable for patients, with no patients requiring dose reduction or discontinuation. This is the first study reporting on the use of an FAK inhibitor combination in MUM.

## 1. Introduction

Uveal melanoma (UM) is the most common intraocular malignant tumor in adults. Primary UMs are effectively treated by plaque radiotherapy or enucleation; however, up to 50% of UM patients who are treated for their primary tumors ultimately succumb to advanced disease [1,2]. The liver is the organ of metastasis in approximately 90% of advanced-stage disease [3,4]. Standard chemotherapies rarely induce clinical responses in patients with macro-metastasis [5,6,7] and 1-year survival of metastatic UM (MUM) patients is less than 30% [8,9]. UM tumors have a low mutational burden [10,11], which is thought to contribute to their low immunogenicity and poor responses to immunotherapy. Objective response rates (ORR) appear low, ranging from 5% with the cytotoxic T-lymphocyte-associated protein 4 (CTLA-4) antibody ipilimumab, 3.6% with anti-programmed cell death 1 (PD-1) antibodies (e.g., nivolumab), 10–21% with combination ipilimumab and nivolumab, and 7.7% with the combination of the lymphocyte activation gene-3 (LAG-3) antibody relatlimab and nivolumab [12,13,14,15,16,17,18]. Tebentafusp was approved for the treatment of MUM as it led to an improvement in overall survival (OS), but it is restricted to patients with HLA-A*02:01, and the ORR was only 9% [19]. Percutaneous hepatic perfusion was also recently approved for the treatment of MUM, but it is limited to patients with less than 50% hepatic involvement and limited extrahepatic metastasis [20]. There is still an urgent need to develop effective therapeutic strategies for MUM patients.

Activating mutations (typically Q209) in genes encoding alpha subunits of the heterotrimeric G proteins, GNAQ and GNA11, are found in 80–90% of UM [21,22,23,24]. Mutant GNAQ and GNA11 signal to several pathways, including MEK-ERK1/2, which are required for tumor cell growth [22,23,25,26]. Unfortunately, inhibition of the MEK/ERK pathway has failed to provide clinical benefits in MUM patients. In a phase II trial of the MEK inhibitor selumetinib (NCT01143402), the partial response rate was 15%, and median progression-free survival (PFS) was improved compared to standard chemotherapy (temozolomide/dacarbazine) (15.9 versus 7.0 weeks, respectively); however, OS improvement did not reach statistical significance, possibly due to the cross-over design of the trial [27]. The SUMIT phase III trial [28] analyzing selumetinib in combination with dacarbazine was terminated early since it showed a poor response rate and only a one-month improvement of median PFS, compared to dacarbazine alone. This suggests that, although MEK/MAPK networks activated by PLC-β may contribute to UM initiation, they may not be critical for the maintenance of tumorigenic potential in UM.

Using a synthetic biology approach and a genome-wide RNAi screen, it was discovered that Gαq activates a highly conserved Rho-GEF, TRIO, and the consequent stimulation of Rho-regulated pathways leading to the activation of YAP, independently of PLC-β, the best-known target of Gαq [29]. The tyrosine kinase FAK was found to provide a direct link between Gαq and tyrosine phosphorylation networks controlling YAP and promotes UM growth [30]. FAK is a cytosolic protein kinase that has multiple effects on a cancer cell and its environment. It is associated with drug resistance, as it can be activated in cancer cells when it is exposed to other signal tyrosine kinase inhibitors. FAK can also be activated by cellular stress and receptor tyrosine kinase inhibitors and signals through critical nodes in the signal transduction pathway such as MEK [31]. Nuclear FAK drives the transcription of chemokines and cytokines, including Ccl5 and TGFβ2, which promote the formation of an immunosuppressive, pro-tumorigenic microenvironment [32]. The kinase activity of nuclear-targeted FAK in squamous cell carcinoma cells has been shown to drive exhaustion of CD8+ T cells and recruitment of regulatory T cells in the tumor microenvironment by regulating chemokine/cytokine and ligand-receptor networks [32]. These changes inhibit antigen-primed cytotoxic CD8+ T cell activity, permitting growth of FAK-expressing tumors. These findings suggest that FAK may have a significant impact on tumor stroma, restoring anti-tumor immunity [32].

Interestingly, UM represents the human cancer harboring the highest level of FAK overexpression [30]. Furthermore, *PTK2* expression (the gene that encodes for FAK) has been significantly correlated with reduced overall survival in UM patients, aligning with its potential biological role in UM [30]. FAK knockdown or CRISPR/Cas9 genome editing reduced UM cell proliferation and tumorigenesis [30]. FAK inhibitors had limited effects in cutaneous melanoma harboring *BRAF* oncogenes, but inhibited FAK (and YAP), and reduced cell proliferation and tumor growth in vivo in UM [31], thereby establishing FAK as a druggable target in UM.

Building on this, a kinome-wide CRISPR-Cas9 sgRNA screen was performed to identify synthetic lethal gene interactions that can be exploited therapeutically [33]. sgRNAs targeting the PKC and MEK-ERK signaling pathways were significantly depleted after FAK inhibition, with ERK activation representing a predominant resistance mechanism. Furthermore, pharmacological inhibition of MEK and FAK showed remarkable synergistic growth-inhibitory effects in UM cells and exerted cytotoxic effects leading to tumor collapse in UM xenograft and liver MUM models in vivo [33].

Defactinib is a small-molecule, orally available selective inhibitor of FAK. Avutometinib is a small-molecule, orally available first-in-class oral RAF/MEK clamp that potently inhibits MEK1/2 kinase activities and induces inactive complexes of MEK with ARAF, BRAF, and CRAF, potentially creating a more complete and durable anti-tumor response through maximal RAS/MAPK pathway inhibition [34,35,36]. In contrast to currently available MEK-only inhibitors, avutometinib blocks both MEK kinase activity and the ability of RAF to phosphorylate MEK. This unique mechanism allows avutometinib to block MEK signaling without the compensatory activation of MEK that appears to limit the efficacy of the MEK-only inhibitors.

Together, avutometinib and defactinib have the potential to offer more complete blockade of the signaling that drives the growth of RAS/MAPK pathway-dependent tumors with the objective of deeper and more durable responses [37]. The combination of defactinib and avutometinib is being evaluated in several clinical trials, and has been recently approved by the FDA for KRAS-mutated recurrent low-grade serous ovarian cancer (LGSOC) [38]. The FRAME study was a phase 1 study investigating the combination in patients with advanced solid tumors including LGSOC, KRAS wild-type or mutant, and non-small cell lung cancer (NSCLC) with KRAS mutations (NCT03875820) [39]. The response rate in subjects with KRAS-mutated LGSOC was 58.3% (7 of 12 subjects with partial response (PR)), and 42.3% (11 of 26 subjects with PR) for all subjects with LGSOC (KRAS wild-type or mutant). The FRAME study led to the development of several tumor specific clinical trials with the combination, including the phase 1/2 RAMP 205 trial evaluating the combination with gemcitabine and nab-paclitaxel in first-line metastatic pancreatic cancer (NCT05669482), the Phase 1/2 RAMP 203 study of KRAS G12C-mutated NSCLC combined with sotorasib (NCT05074810), and the phase 2 RAMP 201 (NCT04625270) and phase 3 RAMP 301 (NCT06072781) trials evaluating the combination in patients with recurrent LGSOC. The phase 2 RAMP 201 trial of defactinib and avutometinib in recurrent LGSOC was notable for a confirmed ORR of 31% with a median duration of response of 31.1 months [39]. Median PFS was 12.9 months. In KRAS-mutated patients, the ORR was even better at 44% and median PFS of 22 months. The combination was generally well-tolerated, with a 10% discontinuation rate due to AEs. The most frequent grade ≥3 treatment-related adverse events (AEs) were elevated creatine phosphokinase (24%), diarrhea (8%), and anemia (5%). Accelerated FDA approval of defactinib and avutometinib for the treatment of adults with KRAS-mutated recurrent LGSOC was obtained on May 8, 2025, based on the phase 2 RAMP 201 trial analysis.

Given the preclinical data demonstrating synergistic efficacy of FAK and RAF/MEK inhibition for UM, and the safety and efficacy demonstrated in other trials, we designed a clinical trial evaluating defactinib and avutometinib in metastatic uveal melanoma.

## 2. Materials and Methods

### 2.1. Study Design

This was an investigator-initiated, prospective, single-arm, single-institution, phase 2 trial evaluating the combination of an FAK inhibitor (defactinib) with a RAF/MEK inhibitor (avutometinib) for the treatment of patients with MUM (ClinicalTrials.gov number, NCT04720417). Patients were eligible if they were ≥18 years with confirmed metastatic UM. Patients could be treatment-naïve or have received prior treatment (4-week washout period required). Defactinib was given 200 mg twice daily, and avutometinib was given 3.2 mg twice a week (e.g., Monday/Thursday or Tuesday/Friday). Both drugs were given for 3 weeks on and 1 week off (28-day cycle). Treatment continued for up to 2 years until disease progression, death, unacceptable toxicity, or withdrawal of consent occurred. Post-treatment, patients were followed for survival until their death or up to 5 years after the last patient was enrolled. Scans were performed every 2 cycles/8 weeks on study with a CT scan of the chest and pelvis and MRI of the abdomen, which were assessed locally. Additional details are provided in the study protocol (Supplementary File S1).

All patients provided written informed consent prior to enrollment into this study. The original protocol and all amendments were approved by the Institutional Review Board at Thomas Jefferson University (IRB#20P.1113). The study was conducted in accordance with the Declaration of Helsinki and Good Clinical Practice guidelines.

### 2.2. End Points and Assessments

The primary objective of the study was to investigate the potential efficacy of the combination of defactinib and avutometinib in patients with MUM. No medication was approved for MUM by the US FDA at the time of the study design, and it was felt that potential clinical benefit could be obtained if MUM patients achieved stable disease (SD); therefore, we considered disease control rate (DCR) of 50% as a meaningful primary endpoint for this early phase clinical study. DCR included complete response (CR), partial response (PR), and stable disease (SD) as determined by RECIST criteria version 1.1 after two cycles. This was considered to be an appropriate goal since the overall response rate (ORR) (CR+PR) and disease control rate (DCR) (CR+PR+SD) of combination treatments using the FDA-approved medications, ipilimumab and nivolumab, were reported to be 11.8–16.7% and 29.4–64%, respectively [15]. The ORR and DCR for tebentafusp were 9% and 46%, respectively [19]. The ORR in MUM is historically quite low across systemic therapies, even with therapies demonstrating overall survival benefit. Even with a low response rate, tebentafusp demonstrated significant overall survival benefit, likely in part driven by patients achieving SD. To avoid premature termination based on low ORR, we decided to use DCR as the primary endpoint of this clinical study. DCR represents a clinically meaningful and statistically feasible primary endpoint and is being considered a valid endpoint in many recent clinical trials [40].

The secondary endpoints of the study were PFS, OS, and safety. PFS was defined as the time from start of first study treatment until confirmed progression of metastasis or death from any cause, or censored at the date of last follow-up visit. OS was defined as the time from start of first study treatment until death from any cause, or censored at the date last known alive. Adverse events were scored with the use of the National Cancer Institute Common Terminology Criteria for Adverse Events, version 5.0.

### 2.3. Biomarker Studies

Although clearly exploratory, we included a couple of biomarker studies to investigate the response and potential resistance mechanisms to study drugs.

### 2.4. ERK Phosphorylation

As part of an exploratory analysis, we studied the ERK activity in blinded pre-treatment and post-treatment tumor biopsies in sections of formalin-fixed, paraffin-embedded tissues. Coded tissue slides were stained without quenching the pigmentation, as the cytoplasmic background did not interfere with the assessment of nuclear immunoreactivity of the phosphorylated, activated form of ERK (pERK). The percentage of cells with nuclear pERK signals was quantified in several areas of interest that covered the entire tumor. Positive nuclear staining was further confirmed using QuPath [41].

### 2.5. RNA-Seq Profiling of Paired Tumor Specimens

We also examined whether any biomarkers were associated with therapeutic sensitivity or disease progression. To explore this, we analyzed RNA gene expression profiles in patient-derived xenograft (PDX) models established from biopsy specimens from patients. The deidentified tumor tissues obtained from patients pre- and post-treatment were implanted into the livers of NSG mice (NOD.Cg-Prkdc scid Il2rg tm1Wjl/SzJ; Jackson Laboratory, Bar Harbor, ME) to establish PDX models (*n* = 2–3 from each tumor tissue) [42]. The animal protocol (ID#01587) was approved by the Institutional Animal Care and Use Committee at Thomas Jefferson University. Mice were housed in a specific pathogen-free facility. The mice were euthanized at 6–7 months post-tumor implantation, and tumor tissues were harvested for subsequent analyses. Two tumor fragments harvested from PDX were processed for RNA extraction using the RNA Mini Kit (Qiagen, Hilden, Germany). RNA-seq was performed by Azenta (Burlington, MA, USA). Raw reads underwent adapter trimming and quality control, followed by a standard pipeline for bulk RNA-seq differential expression analysis. To remove murine contamination inherent to liver PDX samples, trimmed reads were aligned in parallel with STAR to the human GRCh38 reference genome (GENCODE v42) and the mouse GRCm39 genome (GENCODE vM32). Human-specific reads were identified using the Disambiguate tool. Transcript-level abundances were quantified with Salmon in alignment-based mode using GENCODE v42 transcript annotations. Differential expression analysis was performed with DESeq2, and results were visualized with volcano plots generated using the Enhanced Volcano package v1.28.2. Technical replicates, represented by tumor fragments collected from the same PDX tumor, were collapsed prior to differential expression analysis.

### 2.6. Reverse-Phase Protein Array (RPPA)

Reverse-phase protein array (RPPA) profiling was performed by the MD Anderson Cancer Center Functional Proteomics Core Facility using validated antibodies and standardized procedures as previously described [43]. Protein and phosphoprotein abundance levels were quantified using the RPPASPACE algorithm. Level 4 (L4) log2-transformed RPPA data, normalized for sample loading and batch effects, were used for downstream analyses. The RPPA panel included 401 validated antibodies recognizing total and phosphoproteins involved in major signaling pathways. Because RPPA measures a defined antibody panel rather than the full proteome, pathway assignment was restricted to key functional protein and phosphoprotein nodes represented in the RPPA dataset. Pathway-related antibody groups were initially defined using MSigDB pathway annotations and then filtered to include only antibodies available in the RPPA panel. Eight signaling modules were evaluated for their relevance, including FAK/MEK pathway inhibition, inflammatory responses, apoptosis, and potential adaptive resistance mechanisms:

- BCL2 anti-apoptotic pathway (BCL2, BCL-xL, MCL1): reflects pro-survival signaling and resistance to apoptosis.
- Apoptotic priming (BIM, PUMA, BAX, BAK): represents the readiness of cells to undergo apoptosis.
- Apoptosis execution (cleaved caspase-3, caspase-7): indicates active apoptotic cell death.
- MAPK/ERK pathway (pERK, pMEK, p90RSK): reflects canonical downstream signaling of MEK inhibition and potential reactivation.
- FAK/Src signaling (pFAK, pSRC, pPYK2): represents the direct target axis of FAK inhibition.
- JAK/STAT pathway (IL-6, JAK2, STAT3, PD-L1): reflects cytokine-mediated inflammatory signaling, STAT3-dependent survival programs, and immune checkpoint regulation.
- mTORC1 output (pS6K, pS6, p4E-BP1): captures translational and metabolic signaling downstream of growth pathways.
- SGK/mTORC2 pathway (SGK1, SGK3, pNDRG1, pRICTOR): represents an AKT-independent survival axis implicated in resistance.

For visualization, RPPA L4 log2-normalized values were displayed as a descriptive heatmap to provide an overview of pathway-related protein and phosphoprotein abundance patterns. Antibodies were grouped by signaling module, while samples were displayed without unsupervised clustering. No statistical testing was applied to this visualization.

### 2.7. Immunohistochemical Staining of Aldehyde Dehydrogenase 1 Family Member A3 (ALDH1A3)

For immunohistochemistry (IHC), tumor biopsy specimens from patients and PDX specimens were fixed in 10% neutral-buffered formalin overnight at room temperature, embedded in paraffin, and sectioned. Paraffin-embedded tissue sections with 4 μm were deparaffinized, and antigen retrieval was accomplished using TE buffer pH 9.0 with a steamer for 20 min. Sections were incubated with Bioxall blocking solution (Vector Laboratories, Burlingame, CA, USA) followed by rabbit anti-human ALDH1A3 (Proteintech, 25167-1-AP) antibody (1/400) at 4 °C overnight. The following day, sections were incubated for 60 min at room temperature in ImmPRESS AP anti-Rabbit IgG Reagent (Vector Laboratories) and for 7 min in ImmPact Vector Red (Vector Laboratories). Sections were counterstained with hematoxylin (Vector Laboratories) for 30 s at room temperature.

### 2.8. Statistical Analysis

In order to characterize the efficacy of the combination of defactinib and avutometinib in MUM patients, Simon’s Optimal two-stage design was used for conducting this clinical trial [44]. The best response during study treatment in individual patients was used for efficacy analysis. The null hypothesis was that the true response rate is 20%, and the alternative hypothesis was that the true response rate is 50%. Simon’s Optimal two-stage design was selected with a maximum of 18 patients and a target alpha level of 0.05 and power of 80%. The cohort escalation design was guided by a desire to stop the trial early if the actual stabilization rate is 20% or less.

The trial was carried out in two stages. In stage I, a total number of 8 patients would be accrued. If there were 2 or fewer overall responses (CR+PR+SD) among these 8 patients, further enrollment of patients would be stopped with the conclusion that the DCR cannot be 50% or greater. Otherwise, an additional 10 patients would be accrued in stage II, resulting in a total sample size of 18. If there were 7 or more responses among these 18 patients, we would reject the null hypothesis and claim that the treatment is promising [Optimal Design]. With this design, we had no more than a 5% chance of concluding effective (≥50% stabilization rate) when the success rate was at most 20%. Similarly, we had no more than a 20% chance of concluding ineffective (≤20% stabilization rate) when it was effective (50% stabilization rate). If the actual response rate was 20% or worse, we had at least a 0.79 probability that the trial would stop after the first 14 subjects. The overall power of this design was 80.0%.

Documenting anti-tumor activity was the primary objective of this trial. Patients must have received at least one cycle of the trial medication and have had at least one post-baseline RECIST assessment (or have progressed prior to this assessment) to be evaluable for response. To visualize the overall response time, we plotted the response from on-treatment time in swimmer’s plots. Waterfall plots were used to visualize the tumor reduction or increase in size after 2 cycles. Kaplan–Meier curves were constructed to estimate the median PFS and OS.

For the exploratory ERK activity analysis, differences between pre- and post-treatment values were analyzed for each case using the paired *t*-test (GraphPad Prism version 10.0.0 for Windows, GraphPad Software, Boston, MA, USA, www.graphpad.com).

## 3. Results

From February 2021 through January 2023, a total of 13 subjects were enrolled in the study. The study terminated enrollment in April 2023. Trial enrollment was stopped early by study sponsors before the anticipated accrual of 18 patients due to no patients having a significant reduction in disease (only SD, no CR or PR). Stage I enrollment was complete, and the continuation rule was met, but only 4 of the planned 10 patients were enrolled in stage II.

**Figure 1:**
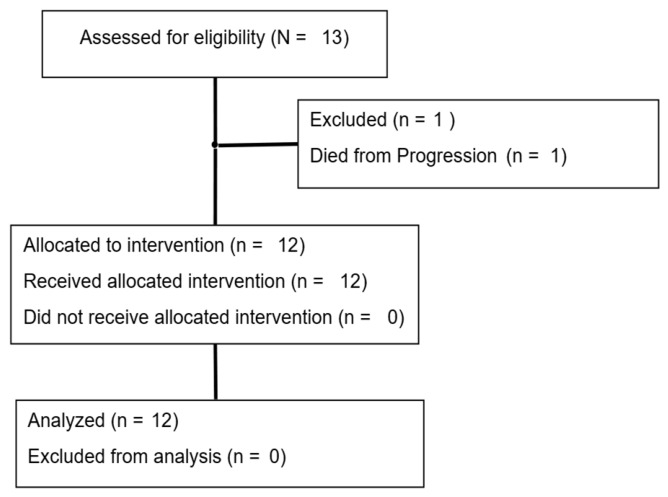
CONSORT diagram.

Of the 13 enrolled patients, one subject did not complete screening due to rapid disease progression. 12 total patients received treatment. In stage I, eight subjects were treated, and DCR was 50% (four patients with SD, four patients with PD), meeting criteria to move on to stage II. In stage II, four additional subjects were treated before the study terminated enrollment. DCR for the 12 patients was 50% (6 patients with SD, 6 patients with PD). All 12 patients have died from UM. The CONSORT diagram can be found in Figure 1.

Patient baseline demographic and clinical information can be found in Table 1. The median age of the patients was 52 years, and 50% were male. Aside from the one patient with a known *SF3B1* mutation that recurred 8 years after her primary eye diagnosis, all other patients developed metastasis within 3 years of their primary eye diagnosis. All patients had hepatic metastases, while eight of the patients had both hepatic and extrahepatic metastases at the time of study enrollment. Eight patients (66.7%) had M1a disease, three patients (25%) had M1b, and only one patient (8.3%) had M1c disease. Median lines of prior therapy were 2 (range 0–6), with three patients (25%) being treatment-naïve. For the nine patients who had prior therapy, median time on the treatment immediately before this trial was 5 months. The time to systemic recurrence from initial eye treatment was 28 months, 36 months, and 12 months in the 3 treatment-naïve patients. The breakdown of each patient’s clinical and demographic information, response to therapy, and treatment pre- and post- progression can be found in Table 2. All but one patient received at least one subsequent treatment prior to death, which varied from liver-directed therapy such as immunoembolization, radioembolization, chemoembolization, to systemic therapy such as tebentafusp, checkpoint inhibitor therapy, and a clinical trial.

**Figure 2:**
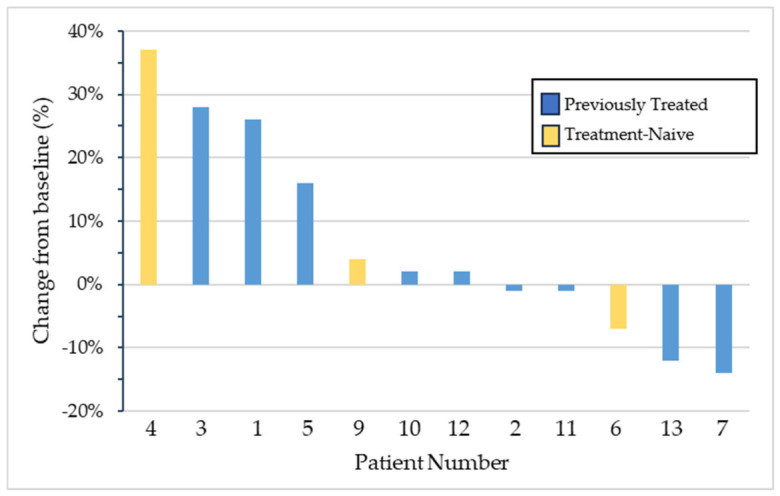
Waterfall plot of best tumor response (*n* = 12).

**Figure 3:**
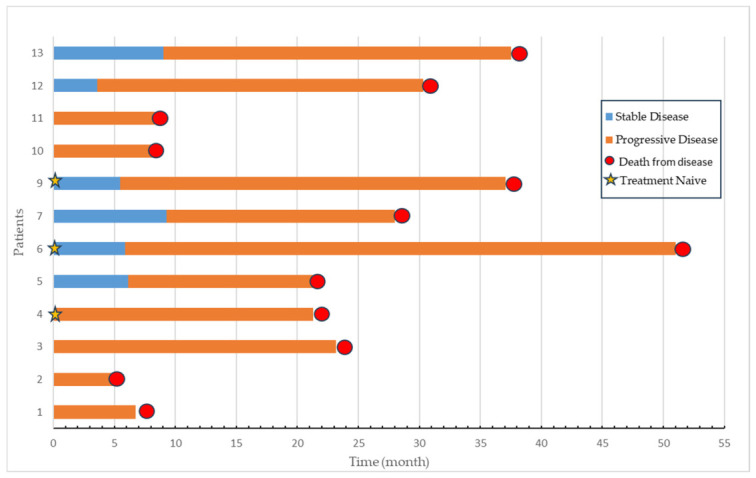
Swimmer’s plot (*n* = 12).

As shown in Table 2, after two cycles, six patients achieved SD (50%) while the other six patients developed PD. No patients achieved CR or PR. Of the three patients who were treatment-naïve, two achieved SD while one patient had PD. Tumor shrinkage was experienced in 58% of patients, or 7 of 12 patients (Figure 2). Patient 2 had a −1% change in the target liver lesions but experienced non-target bone and nodal progression after two cycles, so she had PD at first scans. Best overall response (BOR) was achieved in the first two cycles in most patients, although two patients achieved BOR after four cycles and one patient achieved BOR after four cycles (Table 2). Median duration of treatment was 2.6 months for all patients, and 5.6 months for patients with SD. Median duration of stabilization (time from scan showing SD to scan showing PD) in the six patients who achieved SD was 3.8 months. The three patients with more than 20% liver involvement were all PD. Swimmer’s Plot is shown in Figure 3.

**Figure 4:**
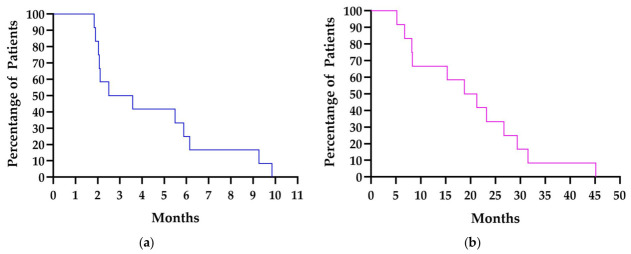
Progression-free survival and overall survival (*n* = 12). (**a**) Progression-free survival; (**b**) Overall survival.

With a median follow-up of 20.0 months, the median PFS was 3.0 months (95% CI 2.1–6.0 months) and median OS was 20.0 months (95% CI 8.3–28.1 months). The Kaplan–Meier curves of PFS and OS are shown in Figure 4. Median PFS and OS were longer for treatment-naïve patients (*n* = 3) vs. the previously treated patients (*n* = 9). Treatment-naïve patients had a median PFS of 5.5 months and median OS of 31.5 months. Previously treated patients had a median PFS of 2.5 months and median OS of 15.3 months. The prolonged OS of the treatment-naïve group may have been driven by post-progression therapy, since the median number of post-progression therapies received before death was 3 for the entire group, 5 for treatment-naïve patients, and 2 for the previously treated patients.

### 3.1. Safety

All treatment-related adverse events (AEs) were Grade 1–2 except one Grade 3 AE of asymptomatic elevated creatine phosphokinase levels (CPK) that resolved with holding treatment and did not recur upon restarting (Table 3). There were no Grade 4 AEs. Patient 13 developed Grade 3 CPK elevation on cycle 1 day 13, which resolved to Grade 1 twelve days later, and treatment was resumed without any dose adjustments. Most frequently reported treatment-related AEs included rash (12/12, 100.0%), diarrhea (8/12, 66.7%), edema (6/12, 50.0%), nausea (6/12, 50%), fatigue (6/12, 50.0%), and blurry vision (6/12, 50.0%).

Because avutometinib is known to cause visual changes, all patients underwent detailed ophthalmological examination at multiple time points (at screening, cycle 2 day 1, cycle 3 day 1, cycle 5 day 1, every 3rd cycle after, and end of treatment). All visual changes on trial were Grade 1, transient, and self-resolved. Four of the patients experienced blurry vision that lasted 24 h or less. Patient 5 had three periods of Grade 1 blurry vision, each lasting 6–15 days, during the first three cycles (none during subsequent cycles). There were no changes in the ophthalmologic examination for patient 5. Patient 13 experienced two episodes of blurry vision during the first two cycles, lasting 8 days and 24 h, respectively. By cycle 3, the blurry vision had completely resolved, but ophthalmologic examination on cycle 3 day 1 was notable for a new small focus of subfoveal fluid in the affected eye on optical coherence tomography scan. Treatment was continued, and on the next ophthalmologic examination the subfoveal fluid had resolved without intervention.

No dose reductions or discontinuations were required for any patients. All side effects resolved after discontinuing trial medications. No treatment-related serious AEs or deaths occurred.

### 3.2. Investigation on Biomarkers Linked to Therapeutic Sensitivity

During this clinical study, biopsy specimens were collected before and after completion of one cycle of combination therapy if clinically feasible. Four biopsy specimens from SD patients (patients 5, 7, 12, 13) and three from PD patients (patients 1, 4, 10) were analyzed for biomarker studies. Additionally, PDX were established from seven metastatic biopsy specimens and used for analysis: four PDX from SD (patients 7, 9, 12, 13) and three PDX from PD (patients 1, 4, 10). Due to the limited amounts of available tissue from individual patients, fewer specimens were used for individual tests, and not all exploratory translational endpoints were able to be evaluated.

### 3.3. Phosphorylation of ERK

**Figure 5:**
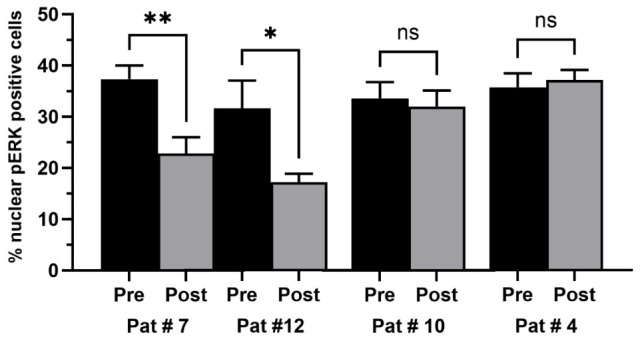
Pre- and post-treatment pERK nuclear staining. ns = not significant. *p* values **: 0.0192; *: 0.0371.

We speculated that if a combination of FAK and RAF/MEK inhibitors works, the downstream activation of the Mitogen-Activated Protein Kinase (MAPK) pathway should be inhibited. We tested four patients whose pre- and post-treatment biopsy samples were available for ERK activity analysis. Reduction in active ERK (pERK) was observed in two patients who achieved SD (patients 7 and 12), but not in two patients who had PD (patients 4 and 10) (Figure 5). This result indicates the presence of alternative pathways to activate ERK despite upstream RAF/MEK inhibition.

### 3.4. RNA Gene Expression Profiles in Patient-Derived Xenograft (PDX)

**Figure 6:**
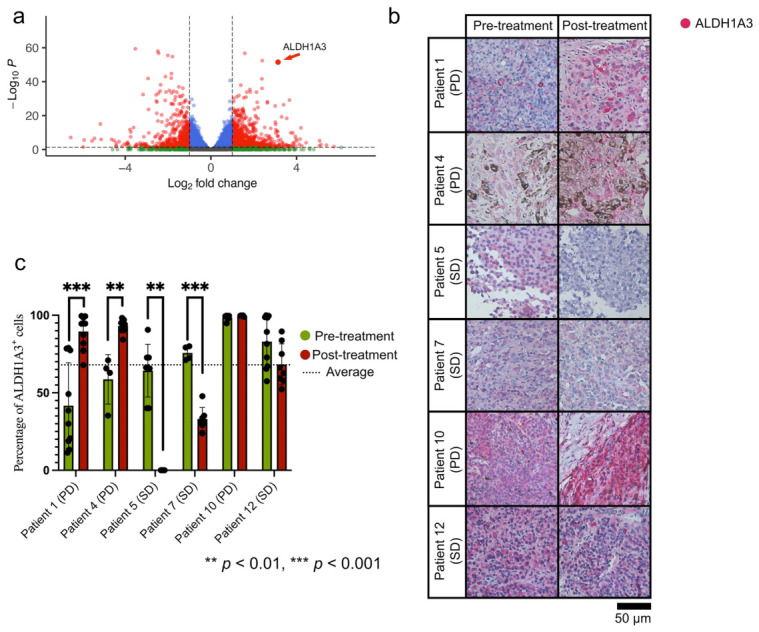
Expression of the ALDH1A3 gene and protein in PDX specimens and biopsy specimens obtained from pre- and post-treatment. (**a**) Volcano plot of RNA-seq data comparing post- and pre-treatment obtained from patient-derived xenograft (PDX) specimens. Dashed lines mark log2FC = 1 and FDR = 0.05. (**b**) Immunohistochemical staining for ALDH1A3 protein in biopsy specimens from MUM patients. ALDH1A3 protein was visualized using an antibody conjugated to alkaline phosphatase. Representative 200× fields are shown; scale bar, 50 μm. (**c**) Quantification of ALDH1A3-positive cells in biopsy specimens from MUM patients. Green bar: pre-treatment, red bar: post-treatment. The dotted line indicates the average level across all samples.

To obtain sufficient tissue for RNA sequencing and to identify potential biomarkers associated with treatment response or disease progression, we established PDX models using pre- and post-treatment biopsy specimens. RNA-seq analysis of matched pre- and post-treatment PDX samples from two patients with PD (patients 1 and 10) showed increased Aldehyde Dehydrogenase 1 Family Member A3 (ALDH1A3) mRNA expression in post-treatment PDX tumors (Figure 6a).

To validate this finding at the protein level, IHC analysis for ALDH1A3 was performed on patient biopsy specimens (Figure 6b) and PDX tumors (Supplemental Figure S1). The percentage of positive tumor cells was quantified in up to 10 non-overlapping high-power fields per sample using Celleste 4.1. The percentage of ALDH1A3-positive cells significantly increased after treatment in two out of the three patients who experienced PD (patients 1 and 4), whereas patients who achieved SD (patients 5, 7, and 12) tended to show a decrease in ALDH1A3 expression in their post-treatment biopsy specimens (Figure 6c). One patient with PD showed no significant change (patient 10). However, in the pre-treatment biopsy, this patient exhibited nearly 100% ALDH1A3-positive cells, which did not change after treatment. In contrast to biopsy specimens, PDX tumors developed from biopsy specimens of patient 10 showed areas of positive and negative tumor cell staining, indicating heterogeneity among the original uveal melanoma cells (Supplement Figure S1). These observations should be considered exploratory because of the limited number of matched PDX specimens.

### 3.5. Investigation on Signaling Modules That Capture the Potential Adaptive Resistance Mechanisms

Since the sample size was very small, this analysis was explorative and performed to obtain baseline data for future investigation on the FAK and MEK inhibitor combination.

**Figure 7:**
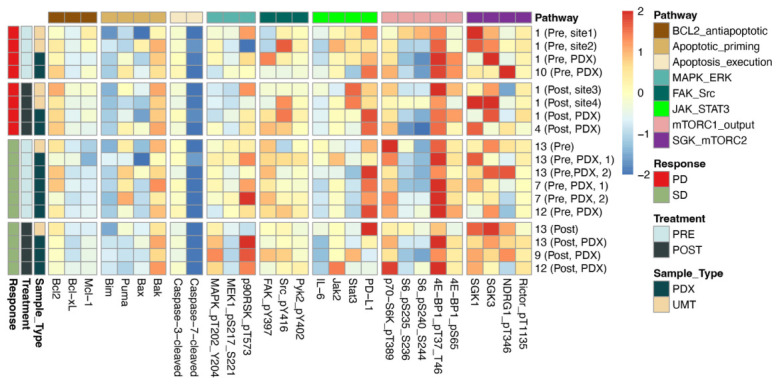
Descriptive heatmap of protein and phosphoprotein abundance across pathway-related RPPA markers. RPPA values are shown as L4 log2-normalized protein and phosphoprotein abundance, where 0 represents the cohort median, +1 indicates approximately 2-fold higher abundance, and −1 indicates approximately 2-fold lower abundance of a given protein or phosphoprotein relative to the cohort. Positive values indicate higher protein abundance and/or phosphorylation (consistent with increased pathway output), whereas negative values indicate lower abundance and/or phosphorylation compared with the typical tumor in the dataset. Antibodies were grouped by signaling module, while samples were displayed without unsupervised clustering. No statistical testing was applied to this visualization. The heatmap should be interpreted as a descriptive overview of pathway-related RPPA patterns. Treatment annotation indicates pre-treatment (Pre) and post-treatment (Post) specimens. Response annotation indicates progressive disease (PD) or stable disease (SD). Sample type annotation indicates metastatic uveal melanoma biopsy specimens (UMT) or patient-derived xenograft tumors (PDX).

As shown in Figure 7, we observed a trend towards persistent pro-survival signaling, especially through mTORC1 and mTORC2 pathways. In particular, the elevated SGK-mTORC2 signaling suggests an AKT-independent survival mechanism of UM cancer cells. Although the results are highly speculative with a small sample size, there was also a trend towards increased BCL2 anti-apoptotic pathway activity in PD tumors, particularly after treatment. Together, these data suggest that resistance to combined FAK/MEK inhibition in UM might be associated with persistent pro-survival signaling, particularly in the mTORC1 and mTORC2 pathways, and with BCL2 expression.

Notably, these features were consistently observed across both patient-derived tumor samples and matched PDX models in our MUM omics database in patients who did not receive FAK and MEK inhibitor treatment (Supplemental Figures S2 and S3), supporting the biological relevance of the observed phenomena in this clinical trial.

## 4. Discussion

We conducted a phase 2, single-arm, single-institution study of the combination of defactinib, an FAK inhibitor, and avutometinib, an RAF/MEK inhibitor, in patients with metastatic uveal melanoma. We treated 12 patients with the combination and achieved SD in 50% of the patients. The median PFS was 3.0 months, and the median OS was 20.0 months with the combination. The combination was quite tolerable for patients, with only one transient and asymptomatic Grade 3 AE of elevated CPK. No patients required dose reduction or discontinuation.

Patients who were treatment naïve had a longer median PFS and OS in comparison to patients who had previous treatment, although the numbers are quite small to make a definitive comparison. Patients who were treatment-naïve received more post-progression therapies, which could have driven the improvement in OS but would not explain the improvement in PFS. Because of the heterogeneity of pre- and post-trial treatments, it is difficult to comment on the influence of specific therapies on the outcomes.

No benefit relative to other available therapies was observed with the combination of avutometinib and defactinib, highlighting the difference in the biology of MUM relative to other cancer types, such as KRAS-mutated LGSOC, in which avutometinib and defactinib have been shown to be effective. There were no CR or PRs seen after 2 cycles in our trial. A DCR of 50% is in line with other systemic therapies for MUM, and our median PFS and OS also are similar to the results of published tebentafusp and ipilimumab/nivolumab studies. Tebentafusp achieved an objective response of 11% compared to 5% in the control group, with a DCR of 46% [45]. Patients who received tebentafusp had a median PFS of 3.4 months and OS of 21.6 months [45]. Similarly, ipilimumab with nivolumab achieved an ORR of 11.5–18%, DCR of 51–63.5%, median PFS of 3.0–5.5 months, and median OS of 12.7–19.1 months [46,47]. Despite strong growth suppression in in vitro experiments with MEK inhibitors, results of other MEK-targeted therapies have also been disappointing, with a systematic review of six clinical trials of MEK inhibitors in MUM showing a median PFS of 3.1 weeks to 16 weeks and overall response rate of 0 to 14% [48]. This discrepancy might indicate the presence of resistance mechanisms or alternative pathways for downstream ERK activation in the tumor microenvironment of patients.

Increasing ALDH1A3 mRNA expression was identified by RNA-seq analysis of PDX specimens and validated at the protein level in patient biopsy specimens, particularly after disease progression. Since PDXs are passaged only in vivo, they avoid the selective culture pressures associated with in vitro. ALDH1A3, an aldehyde-metabolizing enzyme associated with metabolic and oxidative stress, has been reported to exhibit stem cell-like properties. A previous study has shown that ALDH1A3 contributes to therapy resistance in various cancers and is associated with poor survival as well as tumor regrowth post-therapy [49]. Consistent with the literature, increased ALDH1A3 was also observed in MUM patients who experienced PD. ALDH-high tumor subpopulations have been proposed as potential targets for ALDH-directed or ALDH-activated prodrug strategies, including metronidazole-based approaches [50,51]. Although successful PDX engraftment may enrich for biologically aggressive tumor populations and potentially introduce selection bias, the association of ALD1H3 expression was independently confirmed in patient original biopsy specimens, supporting the translational relevance of our finding. However, due to the limited sample size, the findings should be considered exploratory and interpreted with caution. Further investigation with larger patient samples is required to confirm this observation.

Given a lack of tumor response with the combination, other combination strategies are likely necessary to achieve clinical benefit for patients with MUM. A high-throughput chemogenetic drug screen in four genetically distinct *GNAQ*-mutant UM cell lines using a collection of ~2500 mechanistically annotated, oncology-focused agents identified PKC-targeting drugs among the top hits [52]. Among them, darovasertib, a PKC inhibitor under current clinical investigation for the treatment of UM [53], exhibited the most differential Z-AUC score and lowest IC50 across all PKC inhibitors tested. Darovasertib concomitantly inhibits both canonical and non-canonical Gαq-driven signaling pathways (e.g., PKC and PKN/FAK, respectively) [52]. While the blockade of PKC/PKN by darovasertib disrupts Gαq-driven signaling axes, darovasertib alone is insufficient to promote a sustained inhibition of YAP activity downstream of FAK. The partial reduction in FAK activation by darovasertib may sensitize FAK for its further inhibition by direct ATP-competitive kinase inhibitors, thus reinforcing a multipronged pharmacology paradigm that ultimately requires co-targeting both Gαq-regulated signaling axes to achieve a durable clinical benefit. In fact, the combination of darovasertib and an FAK inhibitor has been found to have efficacy in complementary UM PDX models, further supporting the potential combination in MUM [54]. Currently, darovasertib is being evaluated with crizotinib in MUM, and this combination has shown impressive clinical efficacy, with 92% of patients having tumor shrinkage in 68 patients treated in the phase 1/2 dose-escalation trial [53,55]. However, darovasertib with crizotinib has common drug-related AEs that can be challenging for patients. It would be worth investigating whether defactinib in combination with darovasertib could be a more tolerable, efficacious alternative relative to darovasertib with crizotinib.

It is of note that Lubrano et al. recently identified multiple resistance mechanisms by screening 100 pathway-activating mutant complementary DNAs by lentiviral overexpression in *BAP1* wild-type and *BAP-1* mutant UM cells [56]. Using a “signaling toolkit” strategy, their study revealed JAK-STAT activation, overexpression of anti-apoptotic BCL2/BCLXL, and dysregulated PI3K/mTOR or Hippo pathways as escape routes under MEK-ERK or FAK inhibition. In fact, our institutional omics data indicate upregulation of key signaling components in JAK-STAT3 and SGK-mTORC2 pathways in MUM tissue specimens. SGK1 is functionally associated with the PI3K/AKT signaling pathway [57]. However, SGK1 is not a direct component of AKT, but they are parallel downstream effectors of PI3K signaling. As HGF/MET-mediated PI3K/AKT signaling is commonly activated in hepatic UM metastases [58], the increased SGK1 expression observed in tumors may not simply indicate the constitutional pathway activation, but rather may reflect a shift toward SGK1-mediated adaptive and compensatory survival signaling in the liver microenvironment. These data might partly explain the lack of response to treatment with FAK and MEK inhibitors in this clinical trial. Lubrano et al. further demonstrated improved efficacy by adding a BCL2 inhibitor to FAK and MEK [56]. These findings provide a mechanistic rationale for investigating combination strategies with inhibitors targeting compensatory networks of FAK and MEK inhibition, such as mTOR inhibitors and BCL-inhibitors, to overcome resistance mechanisms. Although a 3-drug combination treatment might be clinically challenging, the above in vitro and omics data of MUM suggest directions for improvement.

Our study has several limitations. Our statistical design was based on Simon’s two-stage design, with a planned enrollment of 18 patients during two stages. Although our primary endpoint was DCR of 50% and the DCR of the enrolled 12 patients was 50%, the lack of any CR or PR was disappointing, and thus trial enrollment was stopped early by study sponsors. Therefore, although our data met the goal for the first stage, we were not able to complete the second stage and reject the null hypothesis. As only 12 patients were treated, the limited sample size limits the strength of our conclusions about efficacy. Furthermore, our study is a single-institution small study with significant variation in treatments before and after our trial medication; this may introduce selection bias and also limit generalizability. Molecular information was only available for five of the patients. Further studies with integrated molecular profiling could help identify patients who are more likely to benefit from the combination treatment. Finally, due to limited amounts of available tissue from individual patients, not all exploratory translational endpoints were able to be evaluated. FAK inhibitors have recognized immunomodulatory effects, so future evaluation of the tumor immune microenvironment, such as CD8-positive lymphocytes, macrophage populations, PD-L1 expression, or spatial immune profiling, could also be helpful in better understanding the impact of FAK inhibitors on MUM tumors.

Although the data from this study are preliminary and mostly exploratory, our results suggest that the combination treatment with FAK and MEK inhibitors would not be clinically useful in metastatic uveal melanoma. An improved design for future clinical studies needs to be developed.

## 5. Conclusions

In conclusion, in this phase 2 study of the combination regimen of defactinib and avutometinib, stable disease was achieved in half of patients with MUM. Treatment was well tolerated with only one transient asymptomatic Grade 3 CPK elevation. Although our trial was small and stopped early, it provides important insights into non-canonical targeting of Gαq-driven signaling pathways, including FAK and potential resistance mechanisms. Further research should seek to elucidate an optimal combination treatment strategy for MUM, such as FAK and PKC inhibitors, for a clinically tolerable and efficacious treatment, targeting the signaling pathways downstream of GNAQ/GNA11 driver mutations.

## Figures and Tables

**Table 1:** Baseline demographic and clinical characteristics.

Demographic Characteristics	*n* = 12
Age (median, age)—years	52 (27–72)
Gender, male	6 (50.0%)
Race, white	12 (100.0%)
Primary eye tumor size (mean, range)	Diameter—11.6 mm (8.0–20.0) Thickness—4.1 mm (2.7–20.0)
High-risk molecular features of primary uveal melanoma	Monosomy 3—1 (8.3%) 8q amplification—1 (8.3%) Monosomy 3+8q amplification—6 (50.0%) Class 2—2 (16.7%) Monosomy 3+8q amplification & Class 2—1 (8.3%) Unknown—1 (8.3%)
HLA type	HLA-A*02:01—5 (41.7%) Other HLA—7 (58.3%)
Metastasis stage	M1a (largest ≤ 3 cm)—8 (66.7%) M1b (3.1–8 cm)—3 (25.0%) M1c (>8 cm)—1 (8.3%)
Metastatic organ involvement	Liver—12 (100.0%) Bone—4 (33.3%) Lung—6 (50.0%) Others—6 (50.0%)
Liver tumor burden	<20% involvement—9 (75.0%) 20–50% involvement—3 (25.0%) >50% involvement—0 (0%)
Line of therapy (median, range) Treatment naïve	2 (0–6) 3 (25.0%)

**Table 2:** Detailed patient characteristics.

ID	Age	Mutation *	Stage	% Liver Involved	Extrahepatic Met	Tx Duration (Months)	RECIST Response	BOR		Hepatic PD	Extrahepatic PD	# of Prior Met Tx	Prior Met Tx	Post PD Tx
1	30	GNA11 Q209L	M1c	20–50%	Y	2.1	PD	26% after 2 cycles		Y	Y	6	IE+ipi+VPA, tebe, ipi/nivo, CE, Sys, Sys	CE, Sys Trial, Sys
2	27	BAP1, no GNAQ/11	M1b	20–50%	Y	2.5	PD	−1% after 2 cycles		N	Y	4	IE, RE, CE, Sys	None
3	54	N/A	M1a	<20%	N	1.9	PD	28% after 2 cycles		Y	N/A	1	IE	RE, Tebe, opdualag, Sys
4	60	N/A	M1a	<20%	N	1.8	PD	37% after 2 cycles		Y	N/A	0	None	IE, CE, opdualag
5	40	GNAQ Q209L, SF3B1 R625H	M1a	<20%	Y	6.1	SD	−10% after 6 cycles		Y	N	1	RE+ipi/nivo on trial	IE, CE, Sys
6	72	N/A	M1a	<20%	N	5.9	SD	−7% after 2 cycles		Y	N/A	0	None	IE, tebe, CE, ipi/nivo, opdualag, Sys
7	72	N/A	M1b	<20%	Y	9.3	SD	−14% after 2 cycles		Y	Y	6	Sys Trial, MWA, Tebe, ipi/nivo, Sys, Sys	Sys
9	60	N/A	M1a	<20%	N	5.5	SD	−9% after 4 cycles		Y	N/A	0	None	IE, CE, tebe, nivo, ipi/nivo
10	52	N/A	M1b	20–50%	Y	2.1	PD	2% after 2 cycles		Y	Y	6	IE, RE, CE, Sys, ipi/nivo, Sys	Sys
11	51	N/A	M1a	<20%	Y	2.0	PD	−1% after 2 cycles		N	Y	2	IE, RE	Ipi/nivo, CE
12	51	GNAQ Q209P, BAP1	M1a	<20%	Y	3.6	SD	2% after 2 cycles		N	Y	1	PHP	Sys, Sys, IE, ipi/nivo
13	59	GNA11 Q209L, BAP1	M1a	<20%	Y	9.0	SD	−16% after 4 cycles		N	Y	2	IE, RE	ipi/nivo, opdualag

* Molecular mutational testing was performed on metastatic samples. BOR = best overall response; CE = chemoembolization; IE = immunoembolization; ipi = ipilimumab; Met = metastatic; MWA = microwave ablation; N/A = not available; nivo = nivolumab; PD = progressive disease; PHP = percutaneous hepatic perfusion; RE = radioembolization; SD = stable disease; Sys = systemic therapy clinical trial; tebe = tebentafusp; Tx = treatment; VPA = valproic acid.

**Table 3:** Treatment-related adverse events (*n* = 12).

AE Description	Any Grade	Grade 1	Grade 2	Grade 3	Grade 4
	Number of patients with an event (percent)				
Any adverse event	12 (100.0)	12 (100.0)	9 (75.0)	3 (25.0)	0
Treatment-related adverse events *					
Any	12 (100.0)	12 (100.0)	6 (50.0)	1 (8.3)	0
Rash	12 (100.0)	12 (100.0)	4 (33.3)	0	0
Diarrhea	8 (66.7)	8 (66.7)	1 (8.3)	0	0
Nausea	6 (50.0)	6 (50.0)	0	0	0
Fatigue	6 (50.0)	6 (50.0)	1 (8.3)	0	0
Edema	6 (50.0)	6 (50.0)	1 (8.3)	0	0
Visual impairment	6 (50.0)	6 (50.0)	0	0	0
Pruritis	4 (33.3)	4 (33.3)	0	0	0
Mucositis	4 (33.3)	3 (25.0)	1 (8.3)		
Abdominal pain	3 (25.0)	3 (25.0)	0	0	0
Dysgeusia	3 (25.0)	3 (25.0)	0	0	0
Dizziness	2 (16.7)	2 (16.7)	0	0	0
Anorexia	2 (16.7)	2 (16.7)	0	0	0
Dry skin	2 (16.7)	2 (16.7)	0	0	0
CPK elevation	1 (8.3)	1 (8.3)	1 (8.3)	1 (8.3)	0
Dry Mouth	2 (16.7)	2 (16.7)	0	0	0
Fever	2 (16.7)	1 (8.3)	1 (8.3)	0	0
Alopecia	1 (8.3)	1 (8.3)	1 (8.3)	0	0
Vomiting	1 (8.3)	1 (8.3)	0	0	0
Reflux	1 (8.3)	1 (8.3)	0	0	0
Thrombocytopenia	1 (8.3)	1 (8.3)	0	0	0
Constipation	1 (8.3)	1 (8.3)	0	0	0
Concentration impairment	1 (8.3)	1 (8.3)	0	0	0

* The investigators determined whether adverse events were related to a trial agent. Patients may have had more than one event.

## Data Availability

The original contributions presented in this study are included in the article/Supplementary Material. Further inquiries can be directed to the corresponding author.

## Supplementary Materials

The following supporting information can be downloaded at: https://www.mdpi.com/article/10.3390/cancers18142232/s1, Figure S1: Immunohistochemical staining for the ALDH1A3 protein in matched pre- and post-treatment PDX specimens from MUM patients; Figures S2: Descriptive boxplot of TPM-normalized expression of selected pathway-related genes in an independent institutional cohort of metastatic uveal melanoma biopsy specimens; Figure S3: Descriptive heatmap of pathway-related protein and phosphoprotein abundance measured by RPPA in metastatic biopsy specimens; File S1: Protocol.

## Abbreviations

The following abbreviations are used in this manuscript:

UM	Uveal Melanoma
MUM	Metastatic Uveal Melanoma
ORR	Overall response rate
CTLA-4	Cytotoxic T-lymphocyte-associated protein 4
PD-1	Programmed cell death 1
LAG-3	Lymphocyte activation gene-3
OS	Overall survival
PFS	Progression-free survival
LGSOC	Low-grade serous ovarian cancer
NSCLC	Non-small cell lung cancer
PR	Partial response
CR	Complete Response
SD	Stable Disease
DCR	Disease control rate
AE	Adverse events
pERK	Phosphorylated ERK
PDX	Patient-derived xenograft
RPPA	Reverse-phase protein array
IHC	Immunohistochemical
BOR	Best overall response
CPK	Creatine phosphokinase
