# Supplementary material for: A Phase II Trial of Defactinib Combined with Avutometinib in Patients with Metastatic Uveal Melanoma
Source: Cancers (Basel). 2026 Jul 11;18(14):2232. doi: 10.3390/cancers18142232 (PMC13407007; doi:10.3390/cancers18142232)
Supplement: Supplementary file 1 [file cancers-18-02232-s001.zip › File S1 Protocol.pdf]

THOMAS JEFFERSON UNIVERSITY
Sidney Kimmel Cancer Center

A Phase II trial of Defactinib (VS-6063) combined with VS-6766 (CH5126766) in patients with metastatic uveal melanoma

Principal Investigator:	Rino S. Seedor, MD<br>Department of Medical Oncology<br>215-955-8874
Co-Investigator(s):	Takami Sato, MD, PhD<br>Department of Medical Oncology<br>215-955-1195<br><br>Marlana Orloff, MD<br>Department of Medical Oncology<br>215-955-8874<br><br>Erin Sharpe-Mills, MSN, CRNP<br>Department of Medical Oncology<br>215-955-8874<br><br>Andrew Aplin, PhD<br>Sidney Kimmel Cancer Center<br>Thomas Jefferson University<br><br>Maddi Wechsler, CRNP<br>Department of Medical Oncology<br>215-955-8874
	J. Silvio Gutkind, PhD<br>University of California, San Diego – Moores Cancer Center<br>3855 Health Sciences Dr.<br>La Jolla, CA 92037<br>858-534-5980
Statistician:	Inna Chervoneva, PhD<br>Department of Pharmacology & Experimental Therapeutics<br>215-955-4083
Funding Sponsor:	Verastem Oncology, Inc.<br>117 Kendrick Street, Suite 500<br>Needham, MA 02494<br>781-292-4200
IND/IDE Holder:	Takami Sato, MD, PhD
IND/IDE Number:	153970
Study Product:	VS-6063 (Defactinib) and VS-6766
Protocol IDs:	JeffTrial # 16166<br>IRB Control # 20P.1113

Version Number:	Version Date:
1.0	06/12/2020
1.1	07/18/2020
1.2	09/16/2020
1.3	10/12/2020
1.4	12/11/2020
2.0	08/07/2021
2.1	03/07/2022
3.0	06/21/2022

*Protocol history appears in Appendix IV

CONFIDENTIAL
This document is confidential and the property of THOMAS JEFFERSON UNIVERSITY. No part of it may be transmitted, reproduced, published, or used by other persons without prior written authorization from the study sponsor.

Table of Contents

Signature Page 10
Statement of Compliance 10
List of Abbreviations 11
Study Summary 15
1 Introduction 18
1.1 Background Information 18
Figure 1 20
1.2 Clinical Studies 20
1.3 Potential Risks 21
1.3.1 Potential Risks 21
2 Study Objectives 29
2.1 Objectives 29
2.1.1 Primary 29
2.1.2 Secondary 29
2.1.3 Exploratory 29
2.2 Endpoints/Outcome Measures 29
2.2.1 Primary 29
2.2.2 Secondary 29
2.2.3 Exploratory 30
3 Study Design 30
3.1 Characteristics 30
3.2 Number of Participants 30
3.3 Duration of Therapy 31
3.4 Duration of Follow Up 31
3.5 Treatment Assignment Procedures 31

3.6 Study Timeline 32
3.6.1 Primary Completion 32
3.6.2 Study Completion..32
4 Study Enrollment and Withdrawal 32
4.1 Eligibility Criteria 32
4.1.1 Inclusion Criteria 32
4.1.2 Exclusion Criteria 33
4.2 Gender/Minority/Pediatric Inclusion for Research 36
4.3 Strategies for Recruitment and Retention 36
4.4 Participant Withdrawal...36
4.4.1 Reasons for Withdrawal...36
4.4.2 Handling of Participant Withdrawals and Participant Discontinuation of Study Intervention...37
4.5 Premature Termination or Suspension of Study..37
5 Study Intervention 38
5.1 Study Product 38
5.2 Defactinib (VS-6063) 38
5.2.1 Acquisition...38
5.2.2 Formulation, Packaging, and Labeling...38
5.2.3 Product Storage and Stability 38
5.2.4 Mechanism of action of VS-6063 39
Figure 2...39
5.2.5 Non-clinical anti-tumor activity 40
5.2.6 Toxicology..40
5.2.7 Clinical experience 40
5.2.8 Pharmacokinetics...41
5.2.9 Pharmacodynamics 42

5.2.10 Efficacy ... 42
5.3 VS-6766 (CH5126766) ... 43
5.3.1 Acquisition ... 43
5.3.2 Formulation, Packaging, and Labeling ... 43
5.3.3 Product Storage and Stability ... 43
5.3.4 Mechanism of action of VS-6766 ... 43
5.3.5 Non-clinical anti-tumor activity ... 44
5.3.6 Toxicology ... 45
5.3.7 Clinical experience ... 45
5.3.8 Pharmacokinetics ... 48
5.3.9 Pharmacodynamics ... 48
5.3.10 Efficacy ... 49
5.4 Rationale for the proposed trial and dosing regimen ... 50
5.5 Dosage, Preparation, and Administration ... 50
5.6 Dose Modifications and Dosing Delays ... 51
Table 1: Dose reductions ... 52
5.7 Study Product Accountability ... 53
5.8 Assessing Participant Compliance with Study Product Administration ... 53
5.9 Concomitant Medications/Treatments ... 53
5.10 Dietary Restrictions ... 55
6 Study Schedule ... 55
6.1 Pretreatment Period/Screening ... 55
6.1.1 Obtaining written informed consent ... 55
6.1.2 Evaluations within four weeks (28 days) ... 55
6.1.3 Evaluations within two weeks (14 days) ... 56
6.2 Evaluations during the trial ... 57

6.3 Evaluations at ‘off-study’ visit 58
6.4 Follow-up 59
6.5 Withdrawal Visit/Discontinuation of Therapy 59
6.6 Laboratory Procedures/Evaluations 60
6.6.1 Clinical Laboratory Evaluations 60
6.6.2 Special Assays or Procedures 61
7 Evaluation of Efficacy 62
7.1 Measurement of Effects 63
7.1.1 Measurable and Non-Measurable Disease 63
7.1.2 Baseline documentation of “Target” and “Non-Target” Lesions 63
7.2 Timing of tumor assessments 64
7.2.1 Baseline evaluations 64
7.2.2 Evaluations during and at ‘off-study’ 64
7.3 Evaluation of overall response of metastasis 65
7.4 Response Criteria for metastases 65
Table 2. The definition of response of metastases 66
Table 3. Evaluation of Target and Non-Target Lesions 66
7.4.1 Recording of response in eCRF 67
7.5 Evaluation of progression free survival (PFS) 67
7.6 Evaluation of overall survival (OS) 67
8 Evaluation of Safety 67
8.1 Specification of Safety Parameters 67
8.1.1 Unanticipated Problems 67
8.1.2 Adverse Events 67
8.1.3 Serious Adverse Events 68
8.2 Safety Assessment and Follow-Up 68

8.3 Recording Adverse Events 68
8.3.1 Relationship to Study Intervention 68
8.3.2 Expectedness 69
8.3.3 Severity of Event 69
8.3.4 Intervention 69
8.4 Safety Reporting 69
8.4.1 Reporting to IRB 70
8.4.2 Reporting to SKCC DSMC 71
8.4.3 Reporting of serious adverse events to Verastem Oncology 72
8.4.4 Reporting to FDA 73
8.4.5 Sponsor reporting responsibilities 73
8.4.6 Recording of AE and SAEs in eCRFs 74
8.4.7 Reporting of Pregnancy 74
8.5 Halting Rules 76
9 Study Oversight 77
10 Clinical Site Monitoring and Auditing 78
11 Statistical Considerations 78
10.1 Study Hypotheses 78
10.2 Analysis Plans 78
10.3 Interim Analyses and Stopping Rules 80
10.3.1 Efficacy Review 80
10.4 Defining the end of trial 80
10.5 Sample Size Considerations 80
10.5.1 Replacement Policy 81
10.5.2 Accrual Estimates 81
10.6 Data Analysis 81

10.7 Evaluation of Safety 82
10.8 Pharmacodynamics 82
11 Source Documents and Access to Source Data/Documents 82
12 Quality Control and Quality Assurance 83
13 Ethics/Protection of Human Participants 84
13.1 Ethical Standard 84
13.2 Institutional Review Board 84
13.3 Informed Consent Process 84
13.4 Exclusion of Women, Minorities, and Children (Special Populations) 85
13.5 Participant Confidentiality 85
13.6 Future Use of Stored Specimens and Other Identifiable Data 86
14 Data Handling and Record Keeping 86
14.1 Data Management Responsibilities 86
14.2 Data Capture Methods 86
14.3 Types of Data 87
14.4 Study Records Retention 87
14.5 Protocol Deviations 87
15 Study Finances 88
15.1 Funding Source 88
15.2 Conflict of Interest 88
15.3 Participant Stipends or Payments 88
16 Publication and Data Sharing Policy 88
17 Literature References 90
Appendices 94
APPENDIX A: SCHEDULE OF EVENTS 95
APPENDIX B: MEASUREMENT OF DISEASE (42, 43) 97

APPENDIX C: RECOMMENDATIONS RELATED TO CONTRACEPTION AND PREGNANCY TESTING IN CLINICAL TRIALS (45) 98

APPENDIX D: PROTOCOL HISTORY 99

Signature Page

The signature below constitutes the approval of this protocol and the attachments, and provides the necessary assurances that this trial will be conducted according to all stipulations of the protocol, including all statements regarding confidentiality, and according to local legal and regulatory requirements and applicable US federal regulations and ICH guidelines.

Principal Investigator:

Signed: ______________________________ Date: ______________

Name: Rino Seedor, MD

Title: Assistant Professor of Medical Oncology

Statement of Compliance

This study will be conducted in accordance with the International Conference on Harmonisation guidelines for Good Clinical Practice (ICH E6), the Code of Federal Regulations on the Protection of Human Subjects (45 CFR Part 46), and Thomas Jefferson University research policies.

List of Abbreviations

AE	Adverse Event/Adverse Experience
Alk phos	Alkaline phosphatase
ALT	Alanine aminotransferase
ANC	Absolute neutrophil count
AST	Aspartate aminotransferase
BOR	Best overall response
BP	Blood pressure
^{0}C	Degrees Celsius
CDM	Clinical Data Manager
CFR	Code of Federal Regulations
CI	Chief Investigator
CIOMS	Council for International Organizations of Medical Sciences
CK	Creatine kinase
C_{eff}	Efficacious free concentration
C_{max}	Maximum observed plasma concentration
$C_{ss,ave}$	Average steady-stage concentration
CNS	Central nervous system
CONSORT	Consolidated Standards of Reporting Trials
CPK	Creatine phosphokinase
CR	Complete response
CRA	Clinical Research Associate
CRF	Case Report Form
CRMO	Clinical Research Management Office
CRO	Clinical Research Organization
CSFs	Colony stimulating factors
CSM	Clinical Study Manager
CT	Computerized tomography
CTA	Clinical trial authorization
CTCAE	Common Terminology Criteria for Adverse Events
ctDNA	Circulating free tumor DNA
CTFG	Clinical Trial Facilitation Group
CTM	Clinical Trial Manager
Day	Calendar Day
DCR	Disease control rate

DDU	Drug Development Unit
DLT	Dose limiting toxicity
DSMC	Data and Safety Monitoring Committee
DSMP	Data and Safety Monitoring Plan
DVT	Deep vein thrombosis
ECG	Electrocardiogram
eCRF	Electronic case report form
FAK	Focal adhesion kinase
FDA	Food and Drug Administration
FDG	Fluorodeoxyglucose
FFPE	Formalin fixed and embedded in paraffin
FIH	First in Human
FWA	Federalwide Assurance
GCP	Good Clinical Practice
g/dL	Gram(s) per deciliter
GGT	Gamma-glutamyltranspeptide
GWAS	Genome-Wide Association Studies
Hb	Hemoglobin
HCG	Human chorionic gonadotropin
HIPAA	Health Insurance Portability and Accountability Act
HIV	Human immunodeficiency virus
HR	Hazard ratio
IB	Investigator's Brochure
IC-50	50% inhibitory concentration
ICF	Informed Consent Form
ICH	International Conference on Harmonisation
IDE	Investigational Device Exemption
IMP	Investigational medicinal product
IND	Investigational New Drug Application
IRB	Institutional Review Board
IST	Investigator-sponsored trials
ITF	Investigator trial file
LVEF	Left ventricular ejection fraction
LGSOC	Low-grade serous ovarian cancer
MedDRA	Medical Dictionary for Regulatory Activities

mg/m^2	Milligram per square meter
MOP	Manual of Procedures
MRI	Magnetic resonance imaging
MT/TF	Monday Thursday/ Tuesday Friday
MTD	Maximum tolerated dose
MUM	Metastatic uveal melanoma
N	Number (typically refers to participants)
NA	Not applicable
NCI	National Cancer Institute
NE	Not evaluable
NIH	National Institutes of Health
NSCLC	Non-small cell lung cancer
NYHA	New York Heart Association
OCR	Office for Civil Rights
OHRP	Office for Human Research Protections
OPRR	Office for Protection from Research Risks
OS	Overall survival
PBMC	Peripheral blood mononuclear cells
PD	Pharmacodynamic
PDX	Patient Derived Xenograft
PD-1	Programmed cell death 1
PE	Pulmonary embolism
PET	Positron emission tomography
PFS	Progression-free survival
PHI	Protected Health Information
PI	Principal Investigator
PK	Pharmacokinetic
PR	Partial response
PRC	Protocol Review Committee
PV	Pharmacovigilance
QA	Quality Assurance
QC	Quality Control
QP	Qualified person
Qtc	Corrected QT interval
REC	Research Ethics Committee

RECIST	Response Evaluation Criteria in Solid Tumours
RVO	Retinal vein occlusion
SAE	Serious Adverse Event/Serious Adverse Experience
SD	Stable disease
SDS	Safety Data Sheet (formerly MSDS; Material Safety Data Sheet)
SDV	Source data verification
SKCC	Sidney Kimmel Cancer Center
SOP	Standard Operating Procedure
SUSAR	Suspected unexpected serious adverse (drug) reaction
$T_{1/2}$	Terminal elimination half-life
T_{max}	Time to reach C_{max}
TEAE	Treatment-emergent adverse effects
TJU	Thomas Jefferson University
UAP	Unanticipated Problem
ULN	Upper limit of normal
UM	Uveal melanoma
USM	Urgent safety measure
WBC	White blood cell
WHO	World Health Organization

Study Summary

**Title:**	A Phase II trial of Defactinib (VS-6063) combined with VS-6766 (CH5126766) in patients with metastatic uveal melanoma
**Précis:**	This is a prospective, single arm, phase II clinical trial of the combination of the FAK inhibitor, Defactinib (VS-6063), and the dual RAF/MEK inhibitor, VS-6766 (CH5126766) in patients with metastatic uveal melanoma. The efficacy of this combination treatment will be assessed with the Simon's two stage design. The choice of design is guided by a desire to stop the trial early if the actual stabilization rate (CR + PR + SD) is 20% or less. The recommended doses of the two medications were previously determined in a Phase I study of the combination of Defactinib (VS-6063) and VS-6766 (CH5126766) in patients with advanced solid tumours (IST-VS-6063-003, protocol CCR4642, FRAME).
**Objectives:**	Primary:<br>To investigate the potential efficacy of the combination of Defactinib (VS-6063) and VS-6766 in patients with metastatic uveal melanoma.<br><br>Secondary:<br>(1) To assess the survival benefit of Defactinib in combination with VS-6766 in patients with MUM.<br>(2) To assess the safety and toxicity profile of Defactinib in combination with VS-6766.<br><br>Exploratory:<br>(1) To study the pharmacodynamic profile of Defactinib in combination with VS-6766 in pre-treatment, on-treatment, and post-treatment tumor biopsies.<br>(2) To investigate mechanisms of resistance to the combination of Defactinib and VS-6766.<br>(3) To investigate the potential efficacy of circulating cell free DNA for prediction/monitoring.
**Population:**	Male and female patients with metastases from uveal melanoma; 18 years or older. Sample size will be 18 subjects.
**Phase:**	Phase II
**Number of Sites:**	Thomas Jefferson University Hospital
**Description of Intervention:**	Patients will be treated with a combination of Defactinib (FAK inhibitor) with VS-6766 (dual RAF/MEK inhibitor), given in an intermittent schedule. The dose of Defactinib (VS-6063) will be 200mg twice daily. The dose of VS-6766 (CH5126766) will be 3.2mg

	once a day. Defactinib (VS-6063) will be administered orally twice a day within 30 minutes after a meal. VS-6766 will be administered orally twice a week on Monday/Thursday or Tuesday/Friday at least one hour prior or two hours after a meal. Dose modification will be considered based on toxicity.<br><br>A cycle length is 4 weeks (28 days). Combination dosing (Defactinib and VS-6766) will commence on Cycle 1 Day 1 for 3 weeks followed by one week without either drug in week 4 (i.e. 3 weeks on, 1 week off). If this schedule is not tolerated, alternative schedules may be explored following discussion with Verastem Oncology.
**Study Duration:**	Estimated time from when the study opens to enrollment until completion of data analyses is 42 months.
**Participant Participation Duration:**	Patients may receive treatment until maximum clinical benefit is obtained, the development of intolerable side effects, or progression of disease is confirmed. Post-treatment, patients will be followed for survival until their death or up to 5 years after the last patient is enrolled.
**Estimated Time to Complete Enrollment:**	It is expected that the trial recruitment duration will be 6 to 18 months with an expected accrual rate of approximately 1-3 patients per month at Thomas Jefferson University Hospital. A total of up to 18 patients will be entered into this trial, depending on the disease control rate in the first stage of the study.

Schematic of Study Design:

Time point	Activities
Screening (within 28 days of first dose)	Obtain informed consent, perform baseline assessments - medical history, pre-treatment tumor biopsy, radiological disease assessments, echocardiogram or MUGA, and ophthalmological examination.
Enrollment visit (within 14 days of first dose)	Perform physical exam- clinical disease measurements, vital signs, ECG, baseline laboratory tests, baseline blood samples for PD and ctDNA analysis, and pregnancy test.
Visit 1 (Cycle 1 Day 1)	Perform pre-treatment assessments - physical exam, vitals, laboratory tests (weekly), pregnancy test.<br>Administer initial study drugs.<br>*Ophthalmological examination at end of Cycle 1 before Visit 2.
Visit 2 (Cycle 2 Day 1)	Perform pre-treatment assessments - physical exam, vitals, ECG, laboratory tests (weekly), pregnancy test, toxicity assessment<br>*Ophthalmological examination at end of Cycle 2 before Visit 3.<br>**On-treatment tumor biopsy after Cycle 1 or 2.
Visit 3+ (Day 1 of each cycle)	Continuation of study drugs.<br>Perform follow-up assessments of outcome measures and safety evaluation, physical exam, vitals, labs, pregnancy test, toxicity assessment.<br>*Blood samples for PD cycle on C3D1<br>**Ophthalmological examination on C5D1 and every 3 cycles thereafter.<br>***Disease assessment every 2 cycles.<br>****ctDNA analysis every 2 cycles (up to 6 cycles).
Visit Study Exit Time Point	Perform final Assessments - physical exam, vitals, labs, ECG, pregnancy test, ophthalmological examination, post-treatment tumor biopsy, blood sample for PD and ctDNA analysis, disease assessment, AE evaluation.

1 Introduction

1.1 Background Information

Uveal melanoma (UM) is the most common intraocular malignant tumor in adults. Primary UMs are effectively treated by plaque radiotherapy or enucleation; however, up to 50% of UM patients who are treated for their primary tumors ultimately succumb to advanced disease (1, 2). The liver is the organ of metastasis in ~90% of advanced-stage disease (3, 4). Standard chemotherapies rarely induce clinical responses in patients with macro-metastasis (5-7) and 1-year survival of metastatic UM (MUM) patients is <30% (8, 9). UM tumors have a low mutational burden (10, 11), which is thought to contribute to their low immunogenicity and poor responses to immunotherapy. Objective response rates appear low in retrospective studies, ranging from 5% with ipilimumab, 3.6% with anti-programmed cell death 1 (PD-1) antibodies, and 10-21% with combination ipilimumab and nivolumab (12-17). Currently, there is no Food and Drug Administration (FDA) approved treatment for MUM. There is an urgent unmet need for effective therapeutic strategies for advanced-stage UM.

Activating mutations (typically Q209) in genes encoding alpha subunits of the heterotrimeric G proteins, GNAQ and GNA11, are found in 80-90% of UM (18-21). Mutant GNAQ and GNA11 signal to several pathways including MEK-ERK1/2 which are required for tumor cell growth (19, 20, 22, 23). Unfortunately, inhibition of the MEK/ERK pathway has failed to provide clinical benefits in metastatic uveal melanoma (MUM) patients. In a phase II trial (NCT01143402), the MEK inhibitor, selumetinib, exhibited a 15% partial response rate and improved median progression-free survival (PFS) compared to standard chemotherapy (temozolomide/ dacarbazine) (15.9 versus 7.0 weeks, respectively); however, overall survival improvement did not reach statistical significance, possibly due to the cross-over design of the trial (24). The SUMIT phase III trial (25) analyzing selumetinib in combination with dacarbazine was terminated early since it showed a poor response rate and only a 1 month improvement of median PFS, compared to dacarbazine alone. This suggests that, although MEK/MAPK networks activated by PLC-β may contribute to UM initiation, they may not be critical for the maintenance of tumorigenic potential in UM.

Using a synthetic biology approach and a genome-wide RNAi screen, Dr. Gutkind's group at UCSD have shown that Gαq activates a highly conserved Rho-GEF, TRIO, and the consequent stimulation of Rho-regulated pathways leading to the activation of YAP, independently of PLC-β, the best known target of Gαq (26). Using a novel computational biology approach to identify synthetic lethal gene interactions, his group further discovered that the tyrosine kinase FAK provides a direct link between Gαq and tyrosine phosphorylation networks controlling YAP and promotes UM growth (27).

FAK is a cytosolic protein kinase which has multiple effects on a cancer cell and its environment. It is associated with drug resistance as it can be activated in cancer cells when it is exposed to other signal tyrosine kinase inhibitors. FAK can also be activated by cellular stress and receptor tyrosine kinase inhibitors and signal through critical nodes in signal transduction pathway such as MEK (28). It has been shown that targeting FAK kinase activity has the potential to modulate intra- tumoral Treg levels establishing an immunosuppressive tumor microenvironment. These findings suggest that FAK may have a significant impact on tumor stroma, restoring anti-tumor immunity (29).

Interestingly, UM represents the human cancer harboring the highest level of FAK overexpression (27). Furthermore, *PTK2* expression (the gene that encodes for FAK) has been significantly correlated with reduced overall survival in UM patients, aligning with its potential biological role in UM (27). Dr. Gutkind's group has previously confirmed that a) FAK knock down (KD) or CRISPR/Cas9 genome editing reduces UM cell proliferation and tumorigenesis, b) treatment FAK inhibitors had limited effects in SKCM harboring *BRAF* oncogenes, but inhibited FAK (and YAP), and reduced cell proliferation and tumor growth in vivo in UM (28), thereby establishing FAK as a druggable target in UM.

Building on this, laboratories of Drs. Gutkind, Aplin, and Sato have directed their efforts on identifying combinatorial precision therapies which may be used for the treatment of uveal melanoma. They have found that the combination of FAK and MEK inhibition synergistically inhibits uveal melanoma growth (***Fig. 1A***) and promotes apoptosis in vitro, and reduces xenograft tumor volume in vivo *(**Fig. 1B**).*

Together, the robust preliminary data generated by our team and, the urgent need for therapeutic options for patients with MUM provide a promising opportunity to use this combination in MUM patients. The strong basic science, translational, and clinical expertise of our team put us in an excellent position to conduct bi-directional bench to clinic and clinic to bench translational research.

Figure 1

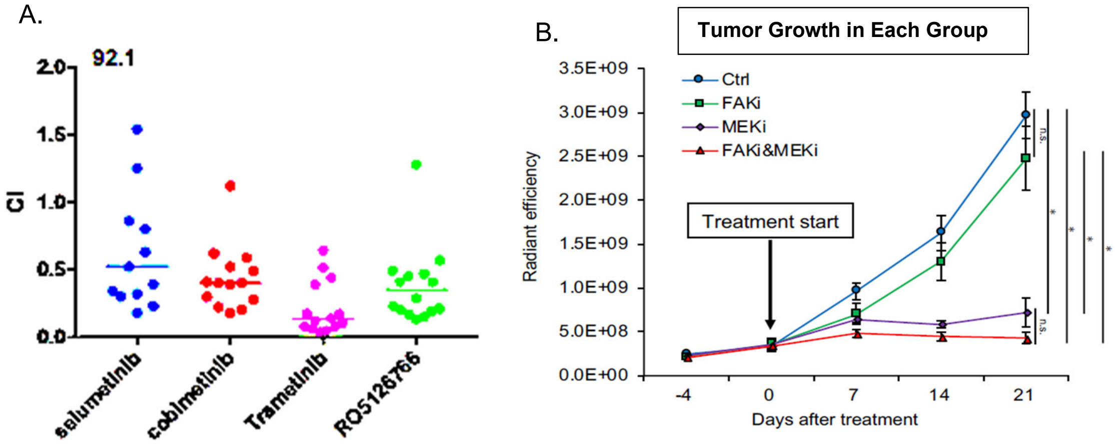

Figure 1: Combination of FAK and MEK inhibitors in uveal melanoma.
A: Synergistic effects of combination treatment with FAK and MEK inhibitors on 92.1 uveal melanoma cell line. The CI value indicates synergism (CI < 1), additive effect (CI = 1), or antagonism (CI > 1) induced by the drug combination. Combination of FAK inhibitor (VS4718) and various MEK inhibitors including RO512766 showed synergistic effects on uveal melanoma cells.
B: Growth suppression of metastatic uveal melanoma (UM001) established in the liver of NSG mice. MEK inhibitor (Trametinib) in combination with FAK inhibitor (VS4718) completely suppressed the growth of metastatic uveal melanoma cells in the liver (red line).

1.2 Clinical Studies

The combination of Defactinib (VS-6063) and VS-6766 (CH5126766) has previously been evaluated in patients with advanced solid tumours (IST-VS-6063-003, protocol CCR4642, FRAME). Preliminary efficacy results are available for a small number of subjects with low-grade serous ovarian cancer (LGSOC, KRAS wild-type or mutant) and non-small cell lung cancer (NSCLC) with KRAS mutations treated with a combination of VS-6766 and Defactinib. The response rates in subjects with KRAS-mutated LGSOC were 67% (4 of 6 subjects with PR) and 50% (4 of 8 subjects with PR) of all subjects with LGSOC (KRAS wild-type or mutant). Five of the 8 subjects had prior MEK inhibitor therapy, including 3 responders. Of the 10 subjects with NSCLC, 1 subject with KRAS (G12V)-mutated cancer achieved PR; 3/10 subjects received treatment for ≥24 weeks. The results of the combination treatment are promising.

Together, the robust preliminary data generated by our team and the urgent need for therapeutic options for patients with MUM provide a promising opportunity to use this combination in metastatic uveal melanoma patients to improve outcomes.

1.3 Potential Risks

The clinical data for both Defactinib (VS-6063) and VS-6766 (CH5126766) can be found in the Investigator Brochures. The safety and tolerability of the combination of the two drugs was shown in the ongoing investigator-sponsored basket study of Defactinib with VS-6766 (IST-VS-6063-003, protocol CCR4642, FRAME).

1.3.1 Potential Risks

1.3.1.1 Toxicity seen in Defactinib (VS-6063) Clinical Studies

A summary of treatment-emergent adverse effects (TEAEs) reported among patients with advanced cancer treated with Defactinib can be found in the IB. Overall, 277 (75.5%) of 367 patients with cancer treated with Defactinib experienced at least 1 TEAE that was considered by the Investigator to be at least possibly related to Defactinib. Overall, GI disorders were the most common type of treatment-related TEAEs reported to date (171 patients; 46.6%), with nausea being the most common individual study drug-related TEAE (85 patients; 23.2%) and diarrhea (76 patients; 20.7%) and vomiting (50 patients; 13.6%) being among the most common. Other common study drug-related TEAEs included fatigue (78 patients; 21.3%), hyperbilirubinemia (48 patients; 13.1%)/blood bilirubin increased (43 patients; 11.7%), and decreased appetite (37 patients; 10.1%).

Overall, 85 (23.2%) of 367 patients with cancer treated with Defactinib experienced at least 1 SAE. The most common types of SAEs were respiratory, thoracic, and mediastinal disorders (20 patients; 5.4%); GI disorders (19 patients; 5.2%); and infections and infestations (15 patients; 4.1%). The most commonly reported serious adverse events by preferred terms were malignant neoplasm progression (16 patients; 4.4%), vomiting (7 patients; 1.9%), dyspnea (6 patients; 1.6%), and nausea and pneumonia (each 5 patients; 1.4%). All other SAEs occurred in ≤3 patients.

Overall, 19 (5.2%) of 367 patients experienced at least 1 serious adverse reaction (SAR) (i.e., an SAE that was considered by the Investigator to be at least possibly related to Defactinib. GI disorders were the most common type of SAR, occurring in 6 (1.6%) patients. The only individual SARs occurring in >1 patient included nausea and vomiting (each 3 patients; 0.8%) and dehydration (2 patients; 0.5%).

In addition, in 3 non-Verastem-sponsored clinical trials, 46 SAEs have been reported in a total of 21 (38.8%) of 54 patients. The most commonly reported SAEs are abdominal pain (7 patients; 13.0%), disease progression (2 patients; 3.7%) and hyponatremia (2 patients; 3.7%). Abdominal pain and disease progression were also reported as SAEs in Verastem-sponsored trials. Hyponatremia however was only reported as an SAE in non-Verastem-sponsored clinical trials. Of the SAEs reported in the 3 studies, SAEs

considered related by the investigator included single reports (1 patient; 1.9%) of abdominal pain, arthralgia, asthenia, back pain, dehydration, nausea, and syncope.

Gastrointestinal Effects
Nausea with or without vomiting and diarrhea are commonly reported with Defactinib. In the VS-6063-202 study in patients with malignant pleural mesothelioma, nausea and diarrhea were reported more commonly in patients on Defactinib than in patients who received placebo, whereas the incidence of vomiting was relatively similar. These events are generally mild or moderate (Grade 1 or 2) in intensity and non-serious, although serious cases have been reported. Prophylactic medications may be used as needed if nausea is found to occur with administration of Defactinib and cannot be managed with small amounts of food. Diarrhea should be managed as described in the clinical study protocol.

Increased Bilirubin
Increased bilirubin has been observed with Defactinib. These increases are generally asymptomatic and not associated with increases in AST or ALT. Increased bilirubin often resolves spontaneously, even with continued drug treatment.

Non-serious adverse events reported from monotherapy studies VS-6063-102, VS-6063-201, VS-6063-202, VS-6063-203, B0761001 (N=313) that are determined by the sponsor to be expected with the use of VS-6063 include hyperbilirubinemia (N=38; 12.1%) and blood bilirubin increase (N=32; 10.2%) with a maximum CTCAE Grade 3.

Drug Interactions
In vitro studies have shown that Defactinib and metabolites inhibits CYP2C9 and/or CYP3A4 in human liver microsomes. Extrapolation to expected human exposure indicates the potential for inhibition or induction of CYP enzymes is low. However, as the clinical significance of these potential interactions has not been formally assessed, it is recommended that compounds that are substrates for CYP2C9 and CYP3A4 are used with caution in concomitant administration with Defactinib may increase their exposure. Defactinib inhibited efflux transporter P-gp (IC=16 μM) and influx transporters OATP 1B1 (IC50= 0.4 μM) and OATP1B3 (IC50=8.5 μM) in vitro. Defactinib and metabolites inhibited UGT1A1 in vitro.

Additionally, Defactinib has been shown to be metabolized by CYP2C9 and CYP3A4, concomitant use of strong inhibitors and inducers of CYP3A4 or CYP2C9 should be avoided if possible or used with caution.

Warfarin
Defactinib may have the potential to increase warfarin exposure due to a potential drug-drug interaction affecting warfarin's metabolism. Based on a review of information from all VS-6063 clinical studies, an increased level of caution is recommended. If patients can safely stop taking warfarin, they should do so. If patients require anti-coagulation, an alternative to warfarin should be considered. Patients who require anti-coagulation but cannot discontinue warfarin should be monitored closely and have their INRs checked more frequently whilst on Defactinib. For patients requiring the start of anti-coagulation therapy during the course of any Defactinib study, alternatives to warfarin are recommended.

Pregnancy and Fertility
Fertility and teratology studies with Defactinib have not been conducted. Safety for women of childbearing capacity cannot be implied from the existing data. It is required for clinical studies that men and women of childbearing potential agree to use adequate contraception (double barrier birth control) for the duration of study therapy and for 3 months after the last dose of Defactinib.

Additional Precautions
As with any investigational agent, there is also the potential risk of toxicities observed in preclinical studies.

With the exception of some hepatobiliary and testicular toxicities, the toxicities observed in the preclinical studies may reasonably be expected to reverse following drug discontinuation. Corresponding slight to marked increases in ALT, AST, ALP, total bilirubin, and GGT were generally seen in affected animals, although individual animal parameters were highly variable. Elevated liver enzymes generally preceded the observation of histopathological findings, although a rigorous time course of enzymes and histological changes was not investigated. These parameters will be routinely monitored.

Defactinib has not been tested for carcinogenic activity in a lifetime rodent bioassay. The risk associated with the finding of in vitro aneuploidy with Defactinib in an advanced cancer population is unknown.

1.3.1.2 **Toxicity seen in VS-6766 (CH5126766) Clinical Studies**
Final safety data are available for two Phase 1 clinical studies (30, 31). The overall frequency of VS-6766 (CH5126766) treatment-emergent adverse events (TEAEs) is based on the data from 52 subjects in Phase 1 study NO21805, with comparative TEAE data provided by Phase 1 study JO22631 in 12 Japanese subjects.

The most commonly reported TEAEs (≥25%) were dermatitis acneiform and rash (67%), diarrhea (65%), blood creatinine phosphokinase increase (58%), asthenia (46%), peripheral edema (40%), nausea (38%), blurred vision (37%), constipation (33%), hypoalbuminemia (33%), vomiting (29%), aspartate aminotransferase (AST) increase (27%), stomatitis (27%) and pyrexia (25%). In general, the available TEAE data from the other studies are consistent with this safety profile.

The identified risks for VS-6766 treatment are skin toxicity, eye toxicity, creatinine phosphokinase elevation, gastrointestinal tract toxicities, and edema. The potential risks are effects on cartilage, genotoxicity, hematological toxicity, drug-drug interactions, liver function abnormalities, renal toxicity, and tissue mineralization.

There were 103 SAEs reported in the total of 131 subjects in monotherapy studies NO31895 (CSR final data), JO22631 (CSR final data), MED18-052 (29 Aug 2019 data cut-off) and ISTCCR3808 monotherapy cohorts (April 1, 2020 data cut-off). There were 43 SAEs judged to be at least possibly treatment related, with most frequent (≥2 events) being rash (8 events), CPK elevation (5 events), increased alkaline phosphatase (2 events) and diarrhea (2 events). The remaining single events were abnormal hepatic function, acute pigment epithelial detachment, abdominal pain, ALT increase, anemia, AST increase, auditory hallucination, blurred vision, bronchial infection, capillary leak syndrome, confusional state, dyspnea, febrile neutropenia, gamma-glutamyltransferase increase, left ventricular dysfunction, myopathy, nausea, pulmonary embolism, pyrexia, retinal detachment, troponin increase, urosepsis, venous embolism, visual hallucinations, visual impairment and vomiting.

The AE and SAE profile of VS-6766 is generally consistent with the AEs reported for other MEK/BRAF inhibitors.

IDENTIFIED RISKS

Skin Toxicity

Skin disorders have been one of the most frequently reported study treatment-related adverse events observed in Study NO21895. Eighty-two treatment-related adverse events were observed in 41 out of 52 subjects in the “skin and subcutaneous tissue disorders” System Organ Class. Thirty-five (35) events of dermatitis acneiform and rash were observed, of which 13 events were Grade 3. In Study JO22631, all 12 subjects experienced AEs reported as treatment-related rash. The most common event was dermatitis acneiform (10 subjects). One subject experienced Grade 3 rash. In Study CCR3808, 40 events of acneiform and/or maculopapular skin rash were reported, of which 10 events were Grade 3.

Rash-related toxicity and the intensity of such reactions were reversible after drug decrease and/or drug interruption, and were associated with adequate medical treatment including corticosteroids (topical and systemic) and antibiotics (topical and systemic).

Recommended guidelines for rash management are as follows:

- Grade 1: No change in study drug. Any of the following: minocycline (recommended dose: 200 mg twice daily [loading dose] followed by 100 mg oral twice daily x 7 to 10 days), topical tetracycline, topical clindamycin, topical silver sulfadiazine, oral diphenhydramine, or oral prednisone (short course) at discretion of the Investigator.
- Grade 2: No change in study drug. Manage as described above for Grade 1 rash.
- Grade 3 (or intolerable Grade 2): Dose reduce or interrupt. Manage as described above for Grade 1 rash. Dose can be re-escalated when rash is Grade ≤2.
- Grade 4: Discontinue subject from study or treat with a reduced dose (previous dose level or intermediate dose after approval of Sponsor) according to clinical judgment. Manage as described above for Grade 1 rash.

Treatment options for rash could also follow recent treatment strategies that have been proposed in published guidelines for effective management of EGFR inhibitor-associated cutaneous toxicities (32). Where possible drug related skin toxicity will be further characterized by photography, paired biopsies (affected and unaffected skin) and in the case of ≥ Grade 3 rash a predose PK blood sample.

Subjects should be advised to avoid sun exposure, and to use sunscreen (sun protection factor [SPF] 30 or higher) containing zinc or titanium oxide rather than synthetic sun blocks, wear protective clothing and apply lip balm with sunscreen. Subjects should use soap-free face cleansers (such as no-rinse cleansing gel twice a day). It is recommended to use an antimicrobial soap, however, to effectively clean areas like the armpits and genitals, which are prone to infection, subjects should use a mild soap for showering. Subjects should use an alcohol-free, oil-free moisturizing cream twice daily. Gloves should be worn to protect the subjects' hands when household cleaning is performed. If subjects experience skin toxicity, they should inform the Investigator and discuss treatment of the skin.

Meanwhile, investigations of intermittent regimens have been implemented in an attempt to decrease the incidence and intensity of skin disorders observed in Study NO21895

while retaining the plasma exposure at levels expected to have a potential for therapeutic benefit.

Eye Toxicity

Eye disorders, including blurred vision and retinal disorders, have been observed in Study NO21895. Fifty-eight adverse events in 36 out of 52 subjects in the “eye disorders” System Organ Class. Blurred vision was observed in 19 events, 3 of which were Grade 3. In Study JO22631, blurred vision occurred in 4 out of 12 subjects, of which all of them were Grade 1 or 2. In Study CCR3808, 20 events of visual abnormalities such as blurred vision were reported, of which one subject experienced Grade 3 blurred vision with Grade 3 acute pigment epithelial detachment.

Complete ophthalmologic examinations should be performed and interpreted by a qualified ophthalmologist, including visual acuity test, corneal examination, intraocular pressure measurements by tonometry, slit-lamp ophthalmoscopy, indirect ophthalmoscopy, spectral domain optical coherence tomography (OCT), digital fundus photography and, if indicated, angiography. Interruption and/or reduction of dosing is recommended for management of eye toxicity. Any abnormality observed by an ophthalmologic examination as well as symptoms should be carefully monitored until improved. Guidelines from a specialized ophthalmologist can be provided if requested by the Investigator.

Creatine Phosphokinase Elevation

In Study NO21895, there were 30 events of blood CPK elevation reported (29 treatment-related events) including 6 Grade 3 events and 1 Grade 4event. In Study JO22631, blood creatine phosphokinase increased in 10 out of 12 subjects, of which all of them were Grade 1 or 2. In study CCR3808, CPK elevation was observed in 29 out of 44 subjects including 4 subjects with Grade 3 events and one subject with a Grade 4 event.

In the event of Grade 3 CPK elevation, Investigators should measure myoglobinuria using dipstick or accurate urine analysis and consider additional diagnostic evaluation (CPK-MB, CPK electrophoresis, troponin) to help determine the etiology of the CPK abnormality and to guide treatment. Investigators should check for any cardiac symptoms and conduct a muscle examination. Information regarding muscle symptoms, exercise, concomitant medications, and alcohol consumption should be recorded.

Regardless of the grade of the CPK elevation, CPK should be monitored until levels return to normal and at least weekly and/or bimonthly as per the protocol.

Gastrointestinal Tract Toxicities

Gastrointestinal toxicities were observed in Study NO21895. A total of 145 adverse events occurred in 45 out of 52 subjects in the “gastrointestinal disorders” System Organ Class. The most commonly reported AEs was diarrhea (34 events). There were no ≥ Grade 3 AEs of gastrointestinal disorders. In Study JO22631, gastrointestinal AEs, which at least 4 out of 12 subjects experienced, were diarrhea (11), stomatitis (9), nausea (4), constipation (4) and decreased appetite (4). There were no ≥ Grade 3 AEs of gastrointestinal disorders.

Recommended guidelines for diarrhea are as follows:

- Grade 1: No change in study drug. Consider treatment with loperamide (4 mg at first onset, followed by 2 mg every 2-4 hours until diarrhea free for 12 hours).
- Grade 2: No change in study drug. Manage as described for Grade 1.
- Grade 3: Decrease dose or interrupt dosing. Manage as described for Grade 1. Dose can be re-escalated when diarrhea is Grade ≤ 1.
- Grade 4: Discontinue subject from study or treat with a reduced dose (previous dose level or intermediate dose after approval of Sponsor) according to clinical judgment. Manage as described for Grade 1.

Interruption and/or reduction of dosing as well as supportive treatment will improve gastrointestinal toxicity in the clinical studies. If gastrointestinal bleeding is suspected, interruption of dosing and a standard work-up of the gastrointestinal tract is required.

Edema

In Study NO21895, 21 events of peripheral edema were observed, 1 of which was Grade 3. In Study JO22631, edema occurred in 5 out of 12 subjects, of which all were Grade 1 or 2.

POTENTIAL RISKS

Effects on the Bone and Cartilage

Relevance of bone and cartilage findings, which were only observed in nonclinical species, is considered low in adult subjects in clinical studies. In rats, bone and cartilage thickening was observed at higher doses, with bone changes partially or completely resolving during the recovery period. A single monkey showed osteoclast activation and decreased osteoblasts at a dose above the repeat dose MTD. There were no effects on bone in monkeys at any dose. Suspicion of bone and cartilage changes should lead to evaluation.

Genotoxicity

No genotoxic potential was observed in any of the tests (Ames test, chromosome aberration test, or in vivo micronucleus test) conducted with VS-6766.

Hematological Toxicity

Hematological toxicity is unlikely and is assessed in clinical studies. Dose interruption and/or dose reduction for management of hematological toxicities is recommended.

Drug-drug interactions

No drug interaction studies have been conducted with VS-6766. In vitro testing demonstrated a low potential for drug-drug interactions mediated by CYP inhibition at pharmacologically relevant concentrations; however, a weak potential for CYP3A4 induction was noted in vitro.

VS-6766 exposure may be affected by CYP3A4 inhibitors or inducers since VS-6766 is mainly metabolized by CYP3A4 enzymes. As such, concomitant administration of CYP3A4 inhibitors and inducers is not recommended.

Liver Function Abnormalities

In Study NO21895, aspartate aminotransferase increase, alanine aminotransferase increase, and blood lactate dehydrogenase increase were reported in 14 events (3 treatment-related), 11 events (2 treatment-related), and 9 events (no treatment-related), respectively. Among them, Grade 3 events were observed (5, 2, 4 events, respectively). In Study JO22631, aspartate aminotransferase increase was observed in 9 out of 12 subjects, blood ALP increase was observed in 5 subjects and ALT increase was observed in 4 subjects. Treatment-related ≥ Grade 3 AEs occurred in one subject each as AST increase, ALT increase and gamma-glutamyl transferase increase. Only the AST increase was determined as Grade 4, and the other seven events were determined as Grade 3.

Liver related laboratory abnormalities, including AST, ALT, and ALP, can be observed in the standard monitoring of blood chemistries in the clinical studies. If any clinically significant increase in liver enzyme level occurs, the dosing should be suspended.

Renal Toxicity

In Study NO21895, only one treatment-related adverse event in one subject was observed in the “renal and urinary disorders” System Organ Class, which was proteinuria. Abnormalities of kidney function, including creatinine and blood urea nitrogen, can be observed through standard monitoring of blood chemistries in the clinical studies. If any

clinically significant increase in creatinine and/or blood urea nitrogen level occur, the dosing should be suspended.

Tissue Mineralization
The relevance of VS-6766 treatment-related tissue mineralization for humans is considered low. Suspicion of ectopic mineralization should lead to evaluation, with consideration of dosing interruption.

2 Study Objectives

2.1 Objectives

2.1.1 Primary

- To investigate the potential efficacy of the combination of Defactinib (VS-6063) and VS-6766 in patients with metastatic uveal melanoma.

2.1.2 Secondary

- To assess the effectiveness of Defactinib in combination with VS-6766 in patients with MUM.
- To assess the safety and toxicity profile of the combination of Defactinib and VS-6766.

2.1.3 Exploratory

- To study the pharmacodynamic profile of Defactinib in combination with VS-6766 in pre-treatment, on-treatment, and post-treatment tumor biopsies.
- To investigate mechanisms of resistance to the combination of Defactinib and VS-6766.
- To investigate the potential efficacy of circulating cell free DNA for prediction/monitoring.

2.2 Endpoints/Outcome Measures

2.2.1 Primary

- Determination of best overall response (BOR) and disease control rate (DCR) (CR+PR+SD) as determined by RECIST criteria version 1.1.

2.2.2 Secondary

- Determination of progression free survival and overall survival.

- Determination of causality of each adverse event to Defactinib and VS-6766 and grading severity according to the National Cancer Institute (NCI) Common Terminology Criteria for Adverse Events (CTCAE) Version 5.0.

2.2.3 Exploratory

- Changes in tumor metabolic activity, signaling to the ERK, YAP, FAK, and PI3K-TOR pathways, changes in UM cell proliferation (Ki67) and apoptosis induction (caspase activation), and changes in tumor microenvironment.
- Detection of biomarkers of resistance.
- Correlation of circulating free DNA with cancer remission and predicting cancer relapse.

3 Study Design

3.1 Characteristics

This is an investigator-initiated, Phase II trial evaluating the combination of a FAK inhibitor (Defactinib, VS-6063) with a RAF/MEK inhibitor (VS-6766, CH5126766) for the treatment of patients with metastatic uveal melanoma.

No medication is approved for MUM by the US FDA and potential clinical benefit could be obtained if MUM patients achieve stable disease (SD); therefore, we will consider Disease Control Rate (DCR) (CR+PR+SD) of 50% as a meaningful endpoint for this early phase clinical study. This is considered to be an appropriate goal since the Overall Response Rates (ORR) (CR+PR) and Disease Control Rate (DCR) (CR+PR+SD) of combination treatments using the FDA approved medications ipilimumab and nivolumab are reported to be 11.8-16.7% and 29.4-64%, respectively (15).

The efficacy of this combination treatment will be assessed using the Simon's two stage design [Optimal Design]. The trial is carried out in two stages. In stage I, a total number of 8 patients are accrued. If there are 2 or fewer overall responses (CR+PR+SD) among these 8 patients, further enrollment of patients may be stopped with the conclusion that the DCR cannot be 50% or greater. Otherwise, an additional 10 patients will be accrued in stage II, resulting in a total sample size of 18. If there are 7 or more responses among these 18 patients, we reject the null hypothesis and claim that the treatment is promising.

3.2 Number of Participants

A total of up to 18 patients will be entered into this trial, depending on the disease control rate in the first stage of the study.

3.3 Duration of Therapy

Treatment should continue for up to 2 years unless (a) the patient asks to be withdrawn, (b) there is evidence of disease progression or (c) the patient is experiencing unacceptable toxicity or for any of the reasons listed in Section 11.4.

In the absence of treatment delays due to adverse event(s), treatment may continue until one of the following criteria applies:

- Disease progression,
- Intercurrent illness that prevents further administration of treatment,
- Unacceptable adverse event(s),
- Patient decides to withdraw from the study, or
- General or specific changes in the patient's condition render the patient unacceptable for further treatment in the judgment of the investigator.

If patients have mild progression (20-30% increase in the sum of the longest diameters from the baseline), and if the Principal Investigator concludes that patients have had clinical benefit from the study medications, the study treatment will be allowed to continue for 2 more cycles as long as patients tolerate the study medications without grade 3 or higher toxicity. If further progression is confirmed with follow-up imaging studies, patients will be removed from the study. Patients who are treated past mild progression must meet the following criteria:

- Absence of symptoms and signs indicating clinically significant progression of disease.
- No decline in ECOG or Karnofsky performance status.
- Absence of symptomatic rapid disease progression requiring urgent medical intervention (e.g., symptomatic pleural effusion, spinal cord compression).

If patients obtain significant benefit from the study treatment (CR or PR), treatment continuation beyond 2 years will be discussed with Verastem Oncology, Inc.

3.4 Duration of Follow Up

Post-treatment, patients will be followed for survival until their death or up to 5 years after the last patient is enrolled.

3.5 Treatment Assignment Procedures

All patients will be given the combination of a FAK inhibitor (Defactinib, VS-6063) with a RAF/MEK inhibitor (VS-6766, CH5126766). There is no randomization procedure or masking procedure.

3.6 Study Timeline

3.6.1 Primary Completion

Estimated time from when the study opens to enrollment until completion of enrollment is 6 to 18 months with an expected accrual rate of approximately 1 to 3 patients per month at Thomas Jefferson University Hospital. Patients may receive treatment until maximum clinical benefit is obtained, the development of intolerable side effects, or progression of disease is confirmed. Post-treatment, patients will be followed for survival until their death or up to 5 years after the last patient is enrolled.

3.6.2 Study Completion

Estimated time from when the study opens to enrollment until completion of data analyses is 42 months.

4 Study Enrollment and Withdrawal

4.1 Eligibility Criteria

4.1.1 Inclusion Criteria

Individuals must meet all of the following inclusion criteria in order to be eligible to participate in the study:

1. Histologically confirmed metastatic uveal melanoma.
2. Predicted life expectancy of at least 12 weeks.
3. ECOG performance status of 0 or 1.
4. Measurable disease according to RECIST 1.1 based on spiral CT or MRI scan, all radiology studies must be performed within 28 days prior to registration.
5. Corrected QT interval (QTc) < 470 ms (as calculated by the Fridericia correction formula, averaged over 3 ECGs).
6. Hematological and biochemical indices within the ranges shown below. These measurements must be performed within two weeks (Day -14 to Day 1) before the patient goes on the trial.

Laboratory Test	Value required
Hemoglobin (Hb)	≥ 9.0 g/dL
Absolute neutrophil count (ANC)	≥ 1.5 x 10^9/L
Platelet count	≥ 100 x 10^9/L
Serum bilirubin	≤ 1.5 x upper limit of normal (ULN)
Albumin	≥ 3.0 mg/dL
Creatine phosphokinase (CPK)	≤ 2.5 x ULN

Alanine aminotransferase (ALT) and/or aspartate aminotransferase (AST)	≤ 2.5 x ULN unless raised due to tumour in which case up to 5 x ULN is permissible
Calculated creatinine clearance	≥ 45 mL/min by the Cockcroft-Gault formula
INR	≤ 1.5 in absence of anticoagulation or therapeutic levels in presence of anticoagulation
PTT	≤ 1.5 x ULN in absence of anticoagulation or therapeutic levels in presence of anticoagulation

7. Patients with adequate cardiac function (left ventricular ejection fraction ≥ 50%) by echocardiography or MUGA scan.
8. No active retinopathy or retinal vein occlusion confirmed by full ophthalmological exam in the eye unaffected by uveal melanoma.
9. Adequate recovery from toxicities related to prior treatments to at least Grade 1 by CTCAE Version 5.0. Exceptions include alopecia and peripheral neuropathy grade ≤ 2. Subjects with other toxicities that are stable on supportive therapy may be allowed to participate with prior approval by the Sponsor.
10. Men and women aged 18 years or over.
11. Females with reproductive potential and their male partners agree to use highly effective method of contraceptive (per Clinical Trial Facilitation Group [CFTG] recommendations in Appendix C during the trial and for 3 months following the last dose of study drug.
12. Written (signed and dated) informed consent and be capable of cooperating with treatment and follow-up.
13. Absence of any psychological, familial, sociological or geographical condition potentially hampering compliance with the study protocol and follow-up schedule.

4.1.2 Exclusion Criteria

An individual who meets any of the following criteria will be excluded from participation in this study:

1. Radiotherapy (except for palliative reasons), endocrine therapy, biological therapy, immunotherapy or chemotherapy during the previous four weeks (six weeks for nitrosoureas, Mitomycin-C) before treatment.

2. Ongoing toxic manifestations of previous treatments. Exceptions to this are alopecia or certain Grade 1 toxicities, which in the opinion of the Investigator and the DDU should not exclude the patient.
3. Known untreated or active central nervous system (CNS) metastases (progressing or requiring corticosteroids for symptomatic control). Patients with a history of treated CNS metastases are eligible, provided they meet all of the following criteria:
   - Evaluable or measurable disease outside the CNS is present.
   - Radiographic demonstration of improvement upon the completion of CNS-directed therapy and no evidence of interim progression between the completion of CNS- directed therapy and the baseline disease assessment for at least 28 days.
4. Gilbert syndrome diagnosed with elevated indirect (unconjugated) bilirubin (>1.2 mg/dl) at least two occasions with normal direct bilirubin in the absence of hemolysis or structural liver damage.
5. Ability to become pregnant (or already pregnant or lactating). However, those female patients who have a negative serum or urine pregnancy test before enrollment and agree to use two medically approved forms of contraception (oral, injected or implanted hormonal contraception and condom, have an intra-uterine device and condom, diaphragm with spermicidal gel and condom) from time of consent, during the trial and for six months afterwards are considered eligible.
6. Male patients with partners of child-bearing potential (unless they agree to take measures not to father children by using one form of medically approved contraception [condom plus spermicide] during the trial and for six months afterwards). Men with pregnant or lactating partners should be advised to use barrier method contraception (for example, condom plus spermicidal gel) to prevent exposure to the fetus or neonate.
7. Major surgery within 4 weeks prior to entry to the study (excluding placement of vascular access), or minor surgery within 2 weeks of entry into the study and from which the patient has not yet recovered.
8. Treatment with warfarin. Patients on warfarin for DVT/PE can be converted to low-molecular-weight heparin (LMWH).
9. Acute or chronic pancreatitis.
10. At high medical risk because of non-malignant systemic disease including active uncontrolled infection.
11. Known to be serologically positive for hepatitis B, hepatitis C or human

immunodeficiency virus (HIV).

12. Patients with the inability to swallow oral medications or impaired gastrointestinal absorption due to gastrectomy or active inflammatory bowel disease.

13. History of abdominal fistula, gastro-intestinal perforation, or diverticulitis.

14. Patients with history of symptomatic cholelithiasis or cholecystitis within six months before enrollment.

15. Concurrent ocular disorders in the eye unaffected by uveal melanoma:
    a) Patients with history of retinal vein occlusion (RVO), predisposing factors for RVO, including uncontrolled hypertension, uncontrolled diabetes
    b) Patients with history of retinal pathology or evidence of visible retinal pathology that is considered a risk factor for RVO, intraocular pressure > 21 mm Hg as measured by tonometry, or other significant ocular pathology, such as anatomical abnormalities that increase the risk for RVO.
    c) Patients with a history of corneal erosion (instability of corneal epithelium), corneal degeneration, active or recurrent keratitis, and other forms of serious ocular surface inflammatory conditions.

16. Concurrent congestive heart failure, prior history of class III/ IV cardiac disease (New York Heart Association [NYHA]), myocardial infarction within the last 6 months, unstable arrhythmias, unstable angina or severe obstructive pulmonary disease.

17. Patients exposed to strong CYP3A4 and strong CYP2C9 inhibitors within 7 days prior to the first dose. Because the lists of these agents are constantly changing, it is important to regularly consult a frequently-updated list such as http://medicine.iupui.edu/clinpharm/ddis/table.aspx; medical reference texts such as the Physicians' Desk Reference may also provide this information.

18. Is a participant or plans to participate in another interventional clinical trial, whilst taking part in this Phase II study of VS-6766 in combination with VS-6063. Participation in an observational trial would be acceptable.

19. Patients with a history of hypersensitivity to any of the inactive ingredients (hydroxypropylmethylcellulose, mannitol, magnesium stearate) of the investigational product.

20. Prior corticosteroids as anti-cancer therapy within a minimum of 14 days of first receipt of study drugs.

21. Any other condition which in the Investigator's opinion would not make the patient a good candidate for the clinical trial.

4.2 Gender/Minority/Pediatric Inclusion for Research

Our study will include both women and minorities but no children. Recruitment of patients will be non-biased, but there are epidemiological differences in the incidence of MUM which may be reflected in the final enrollment numbers.

In population-based epidemiological studies, the age-adjusted incidence of uveal melanoma is higher in males compared with females. However, gender differences in large cohort clinical studies with no age adjustments have not been found (33). We expect about half of our patients to be female (9 of 18 patients).

The relative risk calculation of uveal melanoma in various racial groups (Black, Asian and Pacific Islander, Hispanic, and non-Hispanic White) in 1352 patients using 1992 to 2000 SEER program data revealed the ratios of Black:Asian:Hispanic:non-Hispanic White at 1 : 1.2 : 5 : 19 (33). Therefore, we expect less than 5 patients to be of minority.

4.3 Strategies for Recruitment and Retention

Thomas Jefferson University and Wills Eye Hospital are national referral centers for uveal melanoma. We have been seeing approximately 500 new uveal melanoma patients annually, in which 50-100 patients are metastatic; therefore, accrual of 18 uveal melanoma patients with metastasis within 18 months is feasible. This study will be registered on Clinicaltrials.gov; therefore, referral from other cancer institutes is expected. Furthermore, we will notify melanoma patient-supporting organizations such as MRF, OMF, and Cure-in-Sight once we open this study at Thomas Jefferson University Hospital.

4.4 Participant Withdrawal

4.4.1 Reasons for Withdrawal

Participants are free to withdraw from participation in the study at any time upon request. The following are justifiable reasons for the Investigator to withdraw a patient from trial.

- Unacceptable toxicity (AE/SAE)
- Withdrawal of consent
- Serious violation of the trial protocol (including persistent patient attendance failure and persistent non-compliance)
- Sponsor’s decision to terminate the trial
- Withdrawal by the Investigator for clinical reasons not related to the study medications

- Evidence of disease progression. If patients have mild progression (20-30% increase in the sum of the longest diameters from the baseline) and meet criteria listed in section 3.3, and if Investigators conclude that patients have had clinical benefit from the study medications, the study treatment will be allowed to continue for 2 more cycles as long as patients tolerate the study medications without grade 3 or higher toxicity. If further progression is confirmed with follow-up imaging studies, patients will be removed from the study.
- Symptomatic deterioration
- Pregnancy

Patients that are discontinued from the study will be followed for their survival.

4.4.2 Handling of Participant Withdrawals and Participant Discontinuation of Study Intervention

The Investigator must make every reasonable effort to keep each patient on trial for the whole duration of the trial (i.e. until 28 days after last combination therapy administration). However, if the Investigator removes a patient from the trial or if the patient declines further participation, final 'off-study' assessments should be performed before any therapeutic intervention. All the results of the evaluations and observations, together with a description of the reasons for withdrawal from the trial, must be recorded in the medical records and in the eCRF.

Patients who are removed from the trial due to adverse events (clinical or laboratory) will be treated and followed according to accepted medical practice until adverse events become grade 1 or less, or chronic. All pertinent information concerning the outcome of such treatment must be recorded in the eCRF and on the serious adverse event (SAE) report form where necessary.

Patients who do not complete one cycle of dosing with VS-6766 and Defactinib (VS-6063) for reasons other than toxicity will be replaced.

4.5 Premature Termination or Suspension of Study

This study may be suspended or prematurely terminated if there is sufficient reasonable cause. Written notification, documenting the reason for study suspension or termination, will be provided by the suspending or terminating party to Verastem Oncology and the Food and Drug Administration (FDA). If the study is prematurely terminated or suspended, the principal investigator will promptly inform the IRB and will provide the reason(s) for the termination or suspension.

Circumstances that may warrant termination include, but are not limited to:

- Determination of unexpected, significant, or unacceptable risk to participants.
- Insufficient adherence to protocol requirements.
- Data that is not sufficiently complete and/or evaluable.
- Determination of futility.

5 Study Intervention

5.1 Study Product

This is an investigator-initiated, Phase II trial evaluating the combination of a FAK inhibitor (Defactinib, VS-6063) with a RAF/MEK inhibitor (VS-6766, CH5126766) for the treatment of patients with metastatic uveal melanoma. Both VS-6063 and VS-6766 are investigational medicinal products of Verastem Oncology.

5.2 Defactinib (VS-6063)

Defactinib, or VS-6063, is a small molecule, orally available potent adenosine-5'-triphosphate (ATP)-competitive, reversible inhibitor of focal adhesion kinase (FAK) and proline-rich tyrosine kinase-2 (Pyk2).

5.2.1 Acquisition

Defactinib (VS-6063) will be supplied by Verastem Oncology.

5.2.2 Formulation, Packaging, and Labeling

Defactinib (VS-6063) is formulated as a white to off-white oval tablet for oral administration and supplied in single unit dose strength of 200 mg. In addition to VS-6063, formulation components include microcrystalline cellulose, lactose monohydrate, sodium starch glycolate, and magnesium stearate. VS-6063 tablets are packaged in 120 cc (HDPE) bottles, using an induction seal closure and a white polypropylene child-resistant cap. Packaging and labelling will be prepared to meet all regulatory requirements.

5.2.3 Product Storage and Stability

All supplies must be stored in a secure, limited access storage area.

Defactinib is stable when stored in the defined container closure (HDPE bottles). The labelled storage condition for the drug product is “Store between 15 and 25°C (59 and 77°F).” Bottles are labelled with pill strength and other information as per local regulatory requirements.

Dispensing and storage instructions for Defactinib will be provided in the Pharmacy Manual.

5.2.4 Mechanism of action of VS-6063

FAK and Pyk2 are members of the same family of non-receptor protein tyrosine kinases sharing significant sequence homology and are implicated as important integrating molecules in signal transduction cascades (Figure 2) (34). High expression of the protein tyrosine kinase FAK has been frequently demonstrated to be associated with invasive and metastatic malignancies, implicating FAK in malignant progression of multiple epithelial tumors (35). The role of Pyk2 in tumorigenesis is not as well established. However, recently Pyk2 upregulation was identified as a critical mechanism of feedback following inhibition of FAK function in endothelial cells implicating the dual inhibition of both FAK and Pyk2 as a potential strategy for targeting tumor angiogenesis (36). A comprehensive evaluation of selectivity against a large panel of other kinases in enzyme assays demonstrated that VS-6063 was highly selective for FAK and Pyk2 kinases strongly suggesting that its predominant pharmacologic activity is mediated by inhibition of FAK and Pyk2 kinases.

Figure 2.

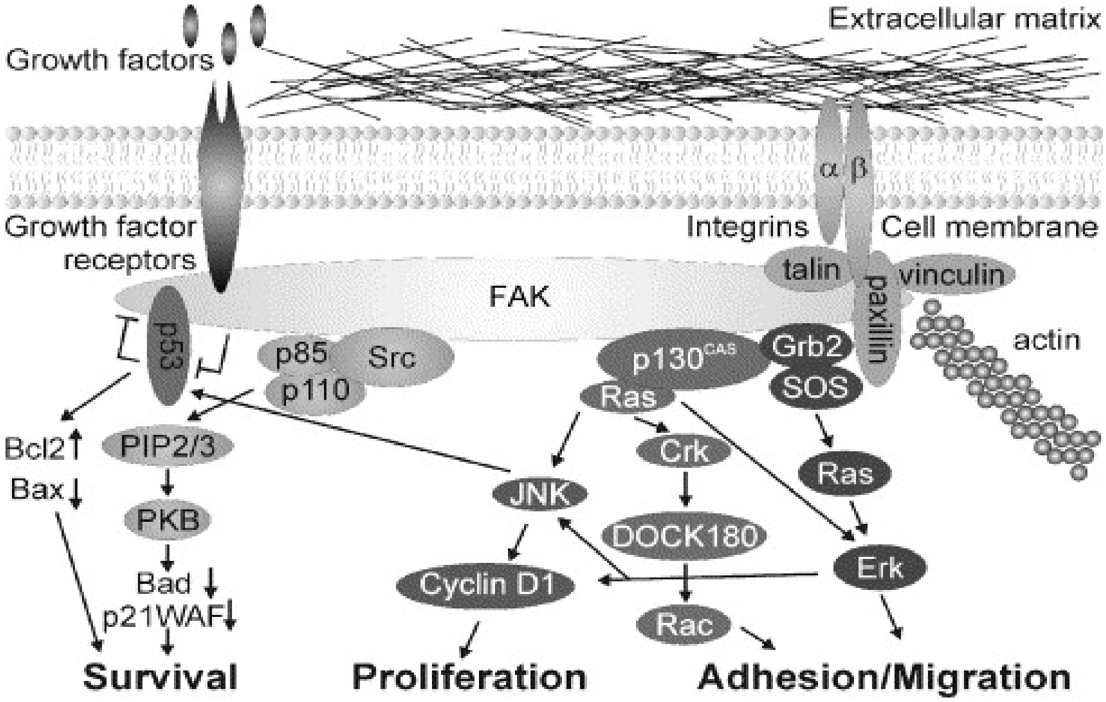

[Biochemical Pharmacology, 73: 597-609, 2007]

Defactinib (VS-6063) has demonstrated antitumor efficacy in multiple human tumor xenograft models in mice including glioma, ovarian, colon, and pancreatic carcinoma

models. In these studies, oral administration of VS-6063 resulted in significant growth inhibition of established tumors at well tolerated dose levels.

5.2.5 Non-clinical anti-tumor activity

Oral administration of Defactinib (VS-6063) demonstrated antitumor efficacy in multiple human tumor xenograft models in mice including glioma, ovarian, colon, pancreatic, breast and mesothelioma models.

For additional information concerning VS-6063, refer to the Investigator Brochure.

5.2.6 Toxicology

A detailed review of nonclinical toxicology, PK and pharmacodynamic findings for Defactinib (VS-6063) are provided in the Investigator's Brochure. Based on the nonclinical studies conducted with VS-6063, the liver and gallbladder, gastrointestinal tract, hematopoietic system, testes/epididymis, and cardiovascular system have been identified as potential targets. With the exception of hepatobiliary and testicular toxicities, the toxicities observed in the nonclinical studies for each of the 2 compounds may reasonably be expected to reverse following drug discontinuation. Appropriate monitoring in humans includes serial liver function testing, routine blood biochemical and hematological parameters, urinalyses, vital signs, and electrocardiograms (ECGs).

5.2.7 Clinical experience

Defactinib (VS-6063) has been evaluated in eleven clinical studies to date: five clinical studies of single-agent treatment with VS-6063 in subjects with non-hematologic malignancies, three combination studies with VS-6063 in subjects with non-hematologic malignancies and three clinical studies in healthy volunteers.

To date, a total of 408 patients have received VS 6063 in Verastem-sponsored clinical trials. Forty-one of these patients received VS- 6063 in healthy volunteer studies (VS-6063-103, VS-6063-105, VS-6063-106), and the remaining 367 patients received VS-6063 for advanced cancer. These include 194 patients who have received VS-6063 in an open-label study (studies B0761001, VS-6063-101, VS-6063-102, VS-6063-104, VS-6063-107, VS-6063-201, and VS-6063-203) and 173 patients who received VS-6063 in one randomized blinded placebo-controlled phase 2 study (study VS-6063-202). Of these 367 patients with cancer, 313 received VS-6063 as monotherapy and 54 received VS-6063 in combination, 22 in combination with paclitaxel, 11 in combination with avelumab and 21 in combination with the Verastem investigational agent VS- 5584. In addition, 171 patients received placebo in a VS-6063 clinical study (Study VS-6063-202).

Additionally, three Investigator-sponsored trials (IST) have been initiated with Defactinib and have enrolled a total of 54 patients: IST-VS-6063-001 (Protocol 201510157, IND

128,094), a Phase 1 study of VS-6063 in combination with pembrolizumab and gemcitabine in patients with advanced cancer (sponsored by Washington University School of Medicine). For this study patient recruitment is currently ongoing. A total of 20 patients have been enrolled and received VS-6063.

IST-VS-6063-002 (Protocol FAK-PD12015, EudraCT 2015-003928-31), a Phase 1/2A study of VS-6063 in combination with pembrolizumab in patients with NSCLC, mesothelioma and pancreatic cancer (sponsored by Cancer Research UK). This study is being conducted in the UK and a total of 4 patients have been enrolled and received VS-6063. IST-VS-6063-004 (IND 126,200), a Phase 2 study of single-agent VS-6063 in patients with tumors with NF2 loss (sponsored by National Cancer Institute). A total of 30 patients have been enrolled and received VS-6063.

Clinical studies conducted to date indicate that VS-6063 has a safety (AE and laboratory) profile that is clinically manageable at the doses administered. Based on the review of the cumulative data the potential risks identified in association with VS-6063 justify the continued investigation of VS-6063 in subjects with advanced cancer.

5.2.8 Pharmacokinetics

Absorption of Defactinib (VS-6063) in rats and dogs was low to moderate. Defactinib showed high plasma protein binding with average unbound fractions similar for mice, dogs, and humans with less binding for rats. Hepatic metabolism was predicted to be the major clearance pathway for Defactinib in humans. Based on in vitro and in vivo evaluations, systemic blood clearance is 3.1 mL/min/kg, volume of distribution at steady state is 1.7 L/kg, elimination half-life is 4.4 hours, and oral bioavailability for Defactinib in humans is projected to be 19%. Assuming complete dissolution, a dosing regimen of 163 mg BID is anticipated to achieve a targeted Css,ave of 133 ng/mL total (13.3 ng/mL free or 26 nM) to produce the intended pharmacologic effects. Twice a day dosing is expected to result in plasma concentrations above Ceff of 133 ng/mL for >15 hours.

In the phase 1 First-in-Human (FIH, B0761001) study, under fasted conditions, single and multiple (Day 15) oral doses of Defactinib were rapidly absorbed and maximum serum concentrations were generally achieved 1 to 2 hours post dose. Cmax and AUC generally increased with increasing dose across the entire dose range, however, interpatient variability was high (>60%). Mean terminal elimination half-life was approximately 9 hours across the dose groups, but individual values showed high variability. Administration with food appeared to delay time to maximal concentration, and there was a trend towards increased exposure, but due to interpatient variability and the small number of patients, this data was inconclusive. Based on the phase 1 PK data for 425 mg BID fasted, the C12 for fasted patients at 400 mg BID (recommended Phase 2 dose) has been estimated to be 250 ng/mL total (25 ng/mL free or 50 nM).

In follow-up studies in healthy males, with tablet formulation at 200 mg (VS-6063-103) and 400 mg (VS-6063-105), exposures increased by approximately 2 to 3-fold following single dose administration of Defactinib in the fed state compared to the fasted state.

5.2.9 Pharmacodynamics

Due to the limited number of patients in each of the dosing cohorts in Study B0761001, no conclusions could be drawn regarding the pharmacodynamics effect of Defactinib (VS-6063) on any of the collected biomarkers in that study. Ongoing clinical studies are continuing to investigate the pharmacodynamics of Defactinib by testing several biomarkers. Pharmacodynamic data from ongoing studies are not yet available.

5.2.10 Efficacy

The clinical data in subjects with KRAS mutant non-small cell lung cancer (Study VS-6063-201) has recently been published. Fifty-five patients with KRAS mutant NSCLC were enrolled across 9 US sites between September 2013 and June 2016 (37). The patients were enrolled to one of the four molecularly defined cohorts. The mean age was 62 years; 51% were female. The median number of prior lines of therapy was 4 (range 1-8). Fifteen (28%) patients met the 12-week PFS endpoint, with one patient achieving a PR. Median PFS was 45 days. All enrolled patients terminated treatment before the study ended. Clinical efficacy did not correlate with secondary mutation status across this KRAS mutant population. Adverse events considered at least possibly related to VS-6063 were experienced by forty-four patients (80%). The majority of these were grade 1 or 2. Fifteen patients (27%) experienced at least possibly related grade 3-5 events, including 1 grade 5 respiratory failure event. Underlying disease was a confounding factor in many patients. The most commonly reported treatment emergent adverse events of any grade were fatigue (35%), nausea (22%), diarrhea (20%), vomiting (18%), and increased bilirubin (16%).

Study VS-6063-202 was terminated due to futility, following a Data Safety Monitoring Board (DSMB) recommendation from a pre-planned interim analysis. The study was not stopped due to any safety concerns with the study drug. The results of the analysis demonstrated that Defactinib was generally well-tolerated, but that there was not a sufficient level of efficacy in this patient setting (maintenance setting in malignant pleural mesothelioma patients with stable disease after ≥4 cycles of pemetrexed/platinum chemotherapy) to warrant continuation of the study.

In Study B0761001 which evaluated the safety and efficacy of multiple doses of Defactinib in patients with an advanced non hematologic malignancy who were unresponsive to currently available therapies or for whom no standard therapy existed, no patient (N=46) had a complete or partial response when treated with Defactinib at any dose level. Stable

disease was achieved in 16 of 37 patients (43%) treated at doses ≥ 100 mg BID. Stable disease was not achieved at doses < 100 mg BID (N=9). Of note, 8 patients enrolled in this study had ovarian cancer. Of these patients, 4 patients achieved stable disease (3 for ≥6 months) and the duration of stable disease with Defactinib was equal to or exceeded that reported with prior therapy.

For further information on clinical studies with VS-6063 please refer to the Investigator Brochure.

5.3 VS-6766 (CH5126766)

VS-6766 (CH5126776) is a dual RAF/MEK inhibitor which inhibits BRAF, CRAF and MEK. It has undergone a phase I trial and expectedly, the dose limiting toxicities have been chronic skin toxicity and acute eye toxicity (30). The drug has shown proof of mechanism inhibition of downstream biomarkers such as p-ERK in normal tissue and in tumor.

5.3.1 Acquisition

VS-6766 will be supplied by Verastem Oncology.

5.3.2 Formulation, Packaging, and Labeling

A complete certificate of analysis and a Qualified Person (QP) certification must be provided with each batch of study medications and be retained in the Investigator Trial File (ITF)/Pharmacy File.

All study drug will be packaged and labelled in accordance with local regulations and Good Manufacturing Practice, stating that the drug is for clinical trial use only and should be kept out of the reach and sight of children. VS-6766 will be labeled, QP released and distributed by Verastem.

VS-6766 will only be dispatched to sites after receipt of confirmation that the regulatory checklist is complete.

VS-6766 will be supplied as hydroxypropyl methyl cellulose (HPMC; hypromellose) capsules for oral administration at a dosage strength of 0.8 mg. The capsule fill consists of VS-6766 and the inactive ingredients mannitol and magnesium stearate.

5.3.3 Product Storage and Stability

All supplies must be stored in a secure, limited access storage area. VS-6766 must be stored at 2-8 C protected from light and moisture.

5.3.4 Mechanism of action of VS-6766

VS-6766 is a highly selective, dual RAF and MEK inhibitor. VS-6766 inhibited RAF family enzymes and MEK1 with a 50% inhibitory concentration (IC50) of 0.056 μM (RAF-1), 0.019 μM (BRAF), 0.0082 μM (BRAF V600E), and 0.16 μM (MEK1). VS-6766 does not inhibit other serine/threonine and tyrosine kinases in enzyme assays against 32 kinases (The Institute of Cancer Research) and in the ATP competitive binding assay against 253 kinases (Ambit Biosciences). VS-6766 is the first example of a tandem MAPK signaling pathway inhibitor and provides a new strategy for cancer therapy.

5.3.5 Non-clinical anti-tumor activity

In in vitro cell-based assays, VS-6766 inhibited the phosphorylation of both MEK1/2 and ERK1/2, the direct substrates of RAF and MEK1/2, respectively, in a dose-dependent manner. The effects of VS-6766 on cell growth from the blocking of the RAF/MEK kinase were evaluated in various cancer cell lines, with wild-type and mutated KRAS and BRAF. A broad spectrum of anti-proliferation activity was observed; in particular in melanoma, non-small cell lung cancer (NSCLC), pancreatic cancer and colorectal cancer cells. For additional information, please refer to the Investigator's Brochure.

In vivo anti-tumor activities of VS-6766 were evaluated in xenograft models. VS-6766 demonstrated a potent and wide spectrum of anti-tumor activities in diverse human tumor xenograft models, including NSCLC, pancreatic, colorectal, melanoma, acute myelocytic leukemia, and hepatocellular carcinoma. Preferential efficacy was observed both in BRAF and RAS mutated tumors. Especially, KRAS and BRAF mutated tumors exhibited better responsiveness to VS-6766 with complete tumor regressions observed.

In a 4-week repeated-dose toxicity study of VS-6766 in rats, skin lesion and tissue mineralization effects on the epithelium and findings in the gastrointestinal tract, the lymphohematopoietic system, the bone, and the liver were observed. Two male rats died during the dosing or withdrawal period. The cause of death was attributed to hemorrhage in the stomach due to vascular rupture related to vessel mineralization.

From the results of safety pharmacology studies, no critical effects on the central nervous, the respiratory, and the cardiovascular systems were observed in rats or monkeys, and an in vitro study revealed that the effect on hERG currents was not concerning. There was no genotoxicity in any of the in vitro and in vivo tests.

VS-6766 markedly suppressed the activation of the MAPK pathway in peripheral blood mononuclear cells (PBMC) as evidenced by the significant inhibition of the PMA-induced phosphorylation of both MEK and ERK. Clear correlation of pharmacokinetics (PK) and pharmacodynamics (PD) was observed.

VS-6766 is mainly metabolized by CYP3A4. Excretion of VS-6766 and its metabolites into urine and feces was almost completed within 48 h post-dose, suggesting both renal and biliary elimination. In rat urine, mainly the demethylated derivative and three unknown metabolites were observed; the parent compound was only a minor component.

VS-6766 showed low potential for drug-drug interactions mediated by CYP inhibition at pharmacologically relevant concentrations. Exposure of VS-6766 may be affected by CYP3A4 inhibitors or inducers. In vitro, VS-6766 had no marked potential for induction of CYP1A2 activity and had weak potential for induction of CYP3A4 activity. It may affect the efficacy of co-administered compounds.

5.3.6 Toxicology

Observed adverse effects following repeated administration were reversible, and they were considered to pose a reasonable safety risk/benefit profile for human subjects receiving a repeated oral administration in a well-controlled clinical setting. Based on the non-clinical safety data available, no major concerns were identified and data supported the Phase I clinical study to investigate the pharmacokinetics, pharmacodynamics, safety, and tolerability of VS-6766 in consenting cancer patients.

5.3.7 Clinical experience

To date, an estimated 198 subjects have been exposed to VS-6766, as monotherapy (n=131) or in combination with other agents (n=67), in 5 clinical studies in subjects with advanced solid tumors or multiple myeloma. These studies included 2 terminated dose-escalation Phase 1 studies (n=64 subjects) and 3 ongoing Phase 1 Investigator-sponsored trials (IST) (n=134 subjects).

The First in Human (FIH) Phase 1 trial was conducted in the Drug Development Unit (DDU) at The Royal Marsden Hospital (NO21895)(30). Expectedly, the dose limiting toxicities were chronic skin toxicity and acute eye toxicity (30). Fifty-two patients received VS-6766 at doses of 0.1–2.7 mg (QD), 2.7–4.0 mg (4-days-on, 3-days-off schedule (4/3)) or 2.7–5.0 mg (7-days-on, 7-days-off schedule (7/7)). The most common dose limiting toxicities (DLTs) were elevated creatine phosphokinase (CPK) n=3 and blurred vision, n=3. MTDs for each of the dosing schedules were 2.25 mg (QD), 4.0 mg (4/3) and 2.7 mg (7/7). The most frequent adverse events (AEs) were rash-related (94.2%) and gastrointestinal disorders, predominantly diarrhea and stomatitis (79%). Cmax was reached 1–2 hours after dosing, with a mean terminal half-life of ~60 hours. Target inhibition was demonstrated in both normal skin and tumor tissue. Tumor shrinkage was observed in 42.2% of patients evaluable for response, including three partial responses (two confirmed and one unconfirmed). Efficacy findings were also supported by fluorodeoxyglucose positron emission tomography (FDG-PET) imaging data, which showed a reduction of FDG uptake in 71% of patients between baseline and Day 15.

VS-6766 PK parameters were linear and time-independent with a substantially longer half-life than has been reported for other specific MEK inhibitors (38-40). Plasma concentrations and exposure increased dose-proportionally and all doses of VS-6766 achieved plasma concentrations known to be effective in preclinical models (41). VS-6766 had a favorable PK/PD profile and encouraging preliminary anti-tumor activity in this population of heavily pre- treated patients, achieving tumor shrinkage in >40% of patients across all dose levels. Based on observed DLTs, MTDs, safety, PK/PD assessments, efficacy and the long-term expected tolerability a dose regimen of 2.7 mg (4/3) was proposed for further investigation in Phase II trials (RP2D).

Study JO22631 was a Phase 1 dose escalation study in 12 Japanese subjects with advanced solid tumors (31). Doses of 0.8, 1.2, 1.8, and 2.25 mg once daily were tested. Three patients were enrolled in each dose cohort for a total of 12 patients. In the dose range tested, there was no evidence of a DLT and the MTD was not identified. As 2.25 mg/day was had already been defined as the MTD of the QD dosing regimen in study NO21895, a decision was made by the sponsor to terminate the study without administering doses higher than 2.25 mg/day. No partial responses were observed in this study, but four of 12 subjects achieved stable disease.

In study JO22631 a total of 10 SAEs occurred in 6/12 subjects, these being 1 subject in each of the 0.8 mg/day and the 1.2 mg/day cohorts, and 2 subjects in each of the 1.8 mg/day and the 2.25 mg/day cohorts (CSR final data). Except for Contrast media allergy (1subject in the 1.2 mg/day cohort), a causal relationship to VS-6766 could not be ruled out in all SAEs, and these included embolism venous (1 subject in the 0.8 mg/day cohort), rash and pyrexia (1 subject in the 1.8 mg/day cohort), AST increase, ALT increase and gamma-glutamyltransferase increase (1 subject in the 1.8 mg/day cohort), rash (1 subject in the 2.25 mg/day cohort), and visual impairment and auditory hallucination (1 subject in the 2.25 mg/day cohort).

IST-MED18-052 is a Phase 1 dose confirmation/expansion IST evaluating VS-6766 single agent in ~31 subjects with advanced NSCLC with KRAS mutation and prior treatment with PD-1/PD-L1 inhibitor. The dose confirmation phase (3+3 design) is assessing the safety and tolerability of 4mg twice weekly VS-6766 dosing, with one possible dose reduction, to select a dose for expansion. The dose expansion phase will treat 25 subjects and assess efficacy (ORR per RECIST version 1.1). Approximately 31 subjects will be enrolled. Ten subjects had been treated as of August 29, 2019. There were a total of 13 SAEs, with 10 at least possibly related to VS-6766 (4 mg BIW) (August 29, 2019 data cut-off). The treatment-related SAEs were Grade 3 rash, Grade 3 abdominal pain, Grade 3 AST increase, Grade 3 ALT increase, Grade 3 alkaline

phosphatase increase, Grade 2 alkaline phosphatase increase, Grade 2 nausea, Grade 2 diarrhea, Grade 3 bilirubin increase, Grade 3 anemia, and Grade 2 vomiting. The study was opened in August 2018 and is ongoing.

IST-CCR3803 is a Phase 1 dose escalation/expansion IST evaluating VS-6766 single agent in ~88 subjects with advanced tumors or multiple myeloma (~54 subjects), and VS-6766 combined with everolimus, a mTORC1 inhibitor, in subjects with advanced solid tumors (~24 subjects). On evaluating two different dosing schedules (twice-weekly and thrice-weekly), the study showed that VS-6766 was more tolerable when administered at an intermittent twice weekly dosing schedule. One subject experienced a DLT in the 4.0 mg thrice-weekly cohort (Grade 3 ophthalmological toxicity – acute pigment epithelial detachment). Two subjects experienced a DLT in the 3.2 mg thrice-weekly cohort; one subject had grade 3 CPK elevation, for which they were hospitalized, and Grade 3 skin rash (face, upper trunk, back, abdomen and both arms); the second subject had Grade 3 skin rash (face, chest and trunk). No subjects experienced a DLT in the 4.0mg, twice weekly cohort. The MTD/RP2D was determined to be 4 mg twice weekly. Approximately 54 subjects will be enrolled in the monotherapy cohorts. A total of 22 subjects have been treated in the dose escalation cohorts and 35 subjects in the single-agent dose (4mg twice weekly) expansion cohorts as of April 1, 2020. Approximately 34 subjects will be enrolled in the combination cohorts and as of April 1,2020 14 subjects have been treated but no results are currently available. The study was initiated in May 2013 and is ongoing.

Finally, IST-VS-6063-003 (protocol CCR4642, FRAME) is a Phase 1 dose escalation/expansion IST evaluating the combination of VS-6766 and Defactinib, a FAK/PYK2 inhibitor, in ~80 subjects with advanced solid tumors, including KRAS-mutant NSCLC, low-grade serous ovarian cancer (KRAS wild-type or mutant) and RAS-mutant colorectal cancer.

Dose escalation was conducted using the following four regimens:

- Cohort 1: 3.2 mg VS-6766 twice weekly + 200 mg Defactinib BID, 3 of 4 weeks
- Cohort 2a: 4 mg VS-6766 twice weekly + 200 mg Defactinib BID, 3 of 4 weeks
- Cohort 2b: 3.2 mg VS-6766 twice weekly + 400 mg Defactinib BID, 3 of 4 weeks
- Cohort 3: 4 mg VS-6766 twice weekly + 400 mg Defactinib BID, 3 of 4 weeks

No subjects experienced a DLT in Cohort 1 or Cohort 2a. There was a DLT of Grade 2 rash in 2 of 3 subjects in Cohort 2b. Cohort 3 was not conducted due to the DLTs in Cohort 2b. Due to chronic Grade 2 toxicities in subjects on treatment > 6 months in Cohort 2a, the Cohort 1 dose of 3.2 mg twice weekly VS-6766 + 200 mg BID Defactinib for 3 of 4 weeks was selected as the RP2D for dose expansion.

Preliminary data from 52 subjects as of 18 Mar 2020 indicated that the most common TRAEs were rash (90%), CPK elevation (56%), hyperbilirubinemia (42%), AST elevation (38%), fatigue (31%), glossitis/oral mucositis/mouth ulcers (31%), ALT elevation (29%), diarrhea (29%), visual disturbance (29%), nausea (25%) and peripheral edema (21%), most of which were Grades 1 or 2 and reversible. Hyperbilirubinemia is an identified risk for Defactinib and was a frequent AE associated with VS-6766 monotherapy. Grade ≥3 TEAEs reported in more than 1 subject were CPK elevation (5/52, 10%), rash (4/52, 8%), anemia (3/52, 6%) and Glossitis/Oral Mucositis/Mouth Ulcers (2/52, 4%).

There were 51 SAEs reported as of 18 Mar 2020, with 4 judged by the Investigator as at least possibly related to study treatment. G2 diarrhea, G3 facial cellulitis and G3 rash were judged by the Investigator to be at least possibly related to only VS-6766, and both treatments possibly related to G2 pneumonitis.

Dose interruptions or modifications were due primarily to rash and CPK elevation. There was 1 AE of bilirubinemia leading to discontinuation. There have been no deaths reported.

Dose expansion is ongoing in cohorts of subjects with KRAS-mutant NSCLC, low-grade serous ovarian cancer (KRAS wild-type or mutant), RAS-mutant colorectal cancer, and advanced solid tumors accessible to biopsy. Approximately 80 subjects will be enrolled. A total of 12 subjects were treated in dose-escalation cohorts and 41 subjects treated in dose-expansion cohorts as of March 18, 2020. The study was initiated in December 2017 and is ongoing.

5.3.8 Pharmacokinetics

Clinical PK data were obtained for daily continuous dosing (studies NO21895 and JO22631) (30, 31), intermittent (4 days on/3 off and 7 days on/7 off) schedules (study NO2189) and twice weekly and thrice weekly dosing regiments (IST CCR3808). VS-6766 exhibited consistent dose-proportional plasma concentration profiles and steady state accumulations supportive of long-term daily dosing in subjects. In study NO21895 and JO22631, maximum plasma concentration (Cmax) occurred 1 to 2 hours after dosing with a mean terminal half-life of approximately 55-60 hours. Apparent systemic clearance was 6 mL/min. Plasma exposure of VS-6766 increased approximately dose-proportionally. Steady state was reached by Day 15 and drug accumulations of between 2- and 7-fold were observed. PK parameters were consistent with that predicted by pre-clinical models (39).

5.3.9 Pharmacodynamics

The Pharmacodynamic effect of VS-6766 was assessed in studies NO21895 and JO22631 by pERK and pMEK suppression in peripheral blood mononuclear cells (PBMCs) and tumor biopsies (NO21895 only) (30, 31). The inhibition of pERK and pMEK

in PBMCs increased proportionally with VS-6766 dose. In NO21895, 6 of 11 subjects with paired tumor biopsies showed a reduction in pERK expression in tumor cells by 20% or more, of which 2 showed a reduction of more than 90% between baseline and day 15. In study IST-CCR3808, matched baseline and post-treatment biopsies from three subjects were obtained and reduction in pMEK (average decrease in cytoplasmic H-score: 66.7%) and pERK expression (average decrease in cytoplasmic H-score: 82.0%) occurred in all three subjects post treatment suggesting therapeutic effects on RAF and MEK activity.

5.3.10 **Efficacy**

VS-6766 has shown preliminary clinical benefit as monotherapy in various advanced solid tumor malignancies (including RAS/RAF melanoma, KRAS NSCLC, and RAS/RAF gynecological malignancies). While studies were not statistically powered to formally assess efficacy, preliminary signs of antitumor effects were observed in several advanced cancers, including tumors with RAS/RAF mutations. In NO21895, tumor shrinkage was observed in 40% (18/45) of subjects including minor response (≥10% shrinkage) in 20% (9/45) of subjects; 7 of 9 minor responses were in subjects with melanoma (30). There were partial responses (PR) per RECIST version 1.1 in 6.7% (3/45) of subjects with melanoma. Two of these subjects with PR had tumors with BRAF(V600E) mutation and one with an NRAS(Q61K) mutation. In study JO22631 five out of 12 subjects achieved stable diseases, including a melanoma subject maximum of 25 % regression (31).

In the KRAS-mutant NSCLC expansion cohort (n=10) in IST-CCR3808, 5 subjects had a tumor reduction of ≥10%, and 3 achieved PR (≥30% reduction). The 3 subjects with PR and 1 with stable disease (SD) had progression-free survival (PFS) >24 weeks, with PFS in 1 subject of >50 weeks and in another subject of >100 weeks. In 5 subjects with RAS/RAF-mutant gynecological cancers (3 ovarian, 2 endometrial), 3 of 5 subjects (2 ovarian, 1 endometrial) achieved a PR5,6. Two of the ovarian subjects with PR had PFS >24 weeks, with PFS in 1 subject >45 weeks.

In IST-VS-6063-003 preliminary efficacy results are available for a small number of subjects with LGSOC (KRAS wild-type or mutant) and NSCLC with KRAS mutations treated with a combination of VS-6766 and Defactinib (VS-6063). The response rates in subjects with KRAS-mutated LGSOC were 67% (4 of 6 subjects with PR) and 50% (4 of 8 subjects with PR) of all subjects with LGSOC (KRAS wild-type or mutant). Five of the 8 subjects had prior MEK inhibitor therapy, including 3 responders. Of the 10 subjects with NSCLC, 1 subject with KRAS(G12V)- mutated cancer achieved PR; 3/10 subjects received treatment for ≥24 weeks.

For additional information concerning VS-6766, refer to the Investigator Brochure.

5.4 Rationale for the proposed trial and dosing regimen

There is evidence that focal adhesion kinase (FAK) activation can drive the emergence of resistance after inhibition of the RAS-RAF-MEK pathway. In this trial, we propose the combination of Defactinib (FAK inhibitor, VS-6063) with VS-6766 (dual RAF/MEK inhibitor), given in an intermittent schedule. Our group has previously found that the combination of FAK and MEK inhibition (specifically VS-6766 with Defactinib) synergistically inhibits uveal melanoma growth and promotes apoptosis in vitro, and reduces xenograft tumor volume in vivo.

From previous phase I and II trials, both VS-6063 and VS-6766 are safe and tolerable. The regimen for Defactinib is based on the findings of the Phase I FIH study and supporting nonclinical data. In the FIH study, subjects received doses of 12.5 to 750 mg twice daily for 21 days. Exposure did not increase significantly above 425 mg twice daily; therefore, the maximum tolerated dose (MTD) was not established and the observed half-life was 9 hours supporting the twice daily dosing schedule. The Phase I trial for VS-6766 evaluated 3 schedules and determined the RP2D as 4mg 2 days per week (Mondays and Thursdays) with high efficacy and lower toxicity compared to more dose intense schedules.

The safety and tolerability of the combination of the two drugs was shown in the ongoing investigator-sponsored basket study of VS-6766 with Defactinib (IST-VS-6063-003, protocol CCR4642, FRAME) conducted by Dr. Banerji in UK. The dosing regimen for Defactinib (VS-6063) is 200mg twice daily administered continuously for 21 days followed by 7 days break, in 28 day cycles. The dosing regimen for VS-6766 is 3.2mg 2 days per week (Mondays and Thursdays), given 3 of 4 weeks, in 28 day cycles.

Together, the robust preliminary data generated by our team and the urgent need for therapeutic options for patients with MUM provide a promising opportunity to use this combination in MUM patients. The strong basic science, translational, and clinical expertise of our team puts us in an excellent position to conduct bi-directional bench to clinic and clinic to bench translational research.

5.5 Dosage, Preparation, and Administration

The dose of VS-6766 will be 3.2mg once a day. VS-6766 will be administered orally once a day on 2 days per week (Mondays and Thursdays or Tuesdays and Fridays) for 3 out of 4 weeks. VS-6766 should be taken 1 hour before or 2 hours after a meal. The dose of Defactinib (VS-6063) will be 200mg twice daily. Defactinib will be administered orally twice a day for 3 out of 4 weeks. Defactinib should be taken within 30 minutes after a meal. One cycle consists of 28 days and neither drug should be taken in the last week of any cycle.

A sufficient supply of study drugs (one cycle worth) will be dispensed to the subject by the Investigational Pharmacy for the subject to take on an outpatient basis between visits.

5.6 Dose Modifications and Dosing Delays

If a patient experiences a clinically significant study drug-related (definite, probable, or possible) Grade 3 or greater toxicity that is not attributable to the disease or disease-related processes under investigation, dosing will be interrupted and supportive therapy administered as required. One or both study medications may be reduced, depending on the causality of the toxicity. If patients experience Grade 4 clinical toxicity or Grade 3 ophthalmological toxicity, the patient will be removed from the study.

The expected toxicity of each drug can be found in section 1.3.1 which can be used to guide investigators in attributing causality to the study drug(s) and dose modification.

The examples of expected toxicities are listed below:
VS-6766
Skin: dermatitis, acneiform rash
GI: diarrhea, constipation, vomiting, stomatitis
General: peripheral edema, pyrexia, asthenia
Eye: blurred vision, retinal disorders
Laboratory abnormalities:

- Blood creatinine phosphokinase increase
- Liver function abnormalities: hypoalbuminemia, AST/ALT increase, ALP increase

Defactinib (VS-6063)
GI: diarrhea, vomiting, abdominal pain
General: fatigue, decreased appetite
Laboratory abnormalities:

- Hyperbilirubinemia
- Low sodium level

Once the drug-related toxicity resolves or reverts to Grade 1, the study treatment is allowed to restart. If the drug-related toxicity resolves or reverts to Grade ≤ 1 within 14 days of onset of treatment with the combination of VS-6766 and Defactinib, both may be restarted at the same dose. This is with the exception of elevated CK or rash; these toxicities should resolve or revert to Grade ≤ 2 within 14 days of onset to restart treatment with the combination of VS-6766 and Defactinib at the same dose. If it is necessary to interrupt the dose again the patient should be treated at a reduced dose level. If it takes more than 14 days until the study-related toxicity resolves or reverts to Grade 1, the dose(s) of study medication will be reduced according to Table 2. If the study-related

toxicity does not resolve or revert to Grade 1 within 28 days, the patient will be removed from the study.

Patients can have more than one dose reduction (see Table 1 below). If a patient cannot tolerate the first reduced dose or in the event a toxicity cannot be clearly associated with one study drug, both drugs will be dose reduced (2nd dose reduction).

If despite appropriate dose reductions and/or delays, the patient experiences unacceptable toxicities specifically attributed to either VS-6766 or Defactinib then consideration will be given to continuing a patient on single agent treatment at the discretion of the Principal Investigator.

In the event of multiple toxicities, dose modification should be based on the worst toxicity observed. Patients are to be instructed to notify Investigators at the first occurrence of any adverse sign or symptom.

Once the dose has been reduced it may not be re-escalated.

If a dose interruption is required within a cycle, the clock does not stop and cycles and visit dates continue until the end of the cycle. If a dose is delayed between cycles, the patient will have unscheduled visits until treatment is restarted at the next cycle. Patients may have their cycle delayed by up to two weeks, but if the dose is held more than 2 cycles the patient will be discontinued from the study.

Adverse events assessed by the Investigator as exclusively related to underlying disease, medical condition or concomitant treatment will not be taken into consideration for treatment interruption and dose reduction.

Table 1: Dose reductions

	VS-6766	Defactinib (VS-6063)
**Starting Dose**	3.2mg once daily MT/TF for 3 out of 4 weeks	200mg twice daily continuous dosing for 3 out of 4 weeks
**1st Dose Reduction for Defactinib**	3.2mg once daily MT/TF for 3 out of 4 weeks	200mg once daily continuous dosing for 2 out of 4 weeks

**1st Dose Reduction for VS-6766**	2.4mg once daily MT/TF for 2 out of 4 weeks	200mg twice daily continuous dosing for 3 out of 4 weeks
**2nd Dose Reduction**	2.4mg once daily MT/TF for 2 out of 4 weeks	200mg once daily continuous dosing for 2 out of 4 weeks

In cases not involving drug related toxicity, study medications may be interrupted for a maximum of 28 days including the scheduled week off.

5.7 Study Product Accountability

Accurate records of all study medication shipments, tablets/capsules dispensed, and all tablets/capsules returned must be maintained. Study medications supplies are to be used only in accordance with this protocol and under the supervision of the Investigator.

The Investigator delegates responsibility for study medication management to the Investigational Pharmacy. The Investigational Pharmacy undertakes not to destroy any unused study medications unless directed to by Verastem Oncology. Any unused study medications must be destroyed according to hospital procedures and properly accounted for using the Study Medication Destruction Form and also on the Study Medication Accountability Record. During the course of the trial the Investigational Pharmacy will check the numbers of capsules of VS-6766/tablets of Defactinib shipped to the center, the number used and the number destroyed or returned. The Investigational Pharmacy will give an account of any discrepancy.

5.8 Assessing Participant Compliance with Study Product Administration

Patients will maintain pill diaries to record compliance to the dosing schedule. At the time of each study visit, patients will bring the pill diaries and the bottle of study medications for pill counts. Study physicians and coordinators will also assess participant compliance during their visit. If non-compliance is identified, it will be recorded in the Case report form (CRF) and continuation of study treatment will be discussed with the patients.

5.9 Concomitant Medications/Treatments

Concomitant medication may be given as medically indicated. Details (including doses, frequency, route and start and stop dates) of the concomitant medication given must be recorded in the patient's medical records and the case report form (CRF). The Principal

Investigator should be alerted if the patient is taking any agent known to affect or with the potential to affect selected CYP450 isoenzymes.

The patient must not receive other anti-cancer therapy or investigational drugs while on the trial.

Radiotherapy may be given concomitantly for the control of pain; however, the irradiated lesions will not be evaluable for response, unless they are clearly progressing at the time of initiating systemic therapy.

In *in vitro* studies, Defactinib (VS-6063) has been shown to be metabolized by CYP2C9 and CYP3A4. *In vitro* studies have shown that VS-6063 inhibits CYP2C9 and CYP3A4 in human liver microsomes. Extrapolation to expected human exposure indicates the potential for inhibition or induction of CYP enzymes is low. VS-6766 is metabolized mainly by CYP3A4.

Therefore, it is recommended that:

- Concomitant use of strong inhibitors of CYP3A4 or CYP2C9 are prohibited. Concomitant use of moderate or weak inhibitors of CYP3A4 or CYP2C9 should be avoided if possible or used with caution.
- Concomitant use of strong inducers of CYP3A4 or CYP2C9 should be avoided if possible or used with caution. Concomitant use of moderate or weak inducers of CYP3A4 or CYP2C9 should be used with caution.
- Compounds that are substrates for CYP3A4 or CYP2C9 should be used with caution.

Because the list of these agents are constantly changing, it is important to regularly consult a frequently-updated list such as http://medicine.iupui.edu/clinpharm/ddis/table.aspx; medical reference texts such as the Physicians' Desk Reference may also provide this information.

If subjects require anticoagulation an alternative to warfarin should be used. Patients who require anticoagulation but cannot discontinue warfarin are excluded from this study. For subjects requiring the start of anti-coagulation therapy during the course of any VS-6063 trial, alternatives to warfarin should be used.

Routine use of colony stimulating factors (CSFs) is not permitted during this study; furthermore, patients should not receive prophylactic growth factors.
Use of erythropoietin is allowed.
Use of stimulators of thrombopoiesis is not allowed.

Supportive or other palliative measures are permitted, with any exceptions as noted.

5.10 Dietary Restrictions

There are no dietary restrictions that participants must adhere to during this trial.

6 Study Schedule

The full schedule of events can be found in **Appendix A.**

In cases where a patient has investigations at a different hospital, for example weekly blood samples, then it is the Investigator's responsibility to ensure he/she receives and reviews the results. The results must be recorded on the electronic case report form (eCRF) and the reports from the other hospitals must be available for source data verification. Laboratory reference ranges, including effective dates, and evidence of laboratory accreditation must be obtained from all laboratories used.

6.1 Pretreatment Period/Screening

Details of all evaluations/investigations for enrolled patients, including relevant dates, required by the protocol must be recorded in the medical records so that the eCRF can be checked against the source data.

6.1.1 Obtaining written informed consent

Written informed consent must be obtained from the patient before any protocol-specific procedures are carried out and within four weeks before the patient's first dose of the study medications (VS-6766 and Defactinib). Informed consent process detailed in section 14.3.

6.1.2 Evaluations within four weeks (28 days)

The following must be performed/obtained within the four weeks before the patient receives the first dose.

- Written informed consent (as detailed in Section 6.1.1);
- Demographic details;
- Medical history including prior diagnosis, prior treatment, concomitant diseases and concomitant treatment;
- Tumor biopsy: the first biopsy should be taken at baseline within 28 days prior to first dose (mandatory) (see Section 6.6.2.1);
- Coagulation: PT, aPTT and INR required to confirm eligibility. Should also be tested prior to each biopsy as per institutional standard practice (at minimum within 7 days prior to biopsy);

Echocardiogram or MUGA scan to ensure adequate cardiac function (left ventricular ejection fraction ≥ 50%);
Radiological disease assessments: Computerized Tomography (CT) scan of the chest and pelvis; and Magnetic Resonance Imaging (MRI) scan of the abdomen. Radiological measurements must be performed within four weeks of the patient's first dose;
Complete ophthalmologic examinations should be performed and interpreted by a qualified ophthalmologist, including visual acuity test, corneal examination, intraocular pressure measurements by tonometry, slit-lamp ophthalmoscopy, indirect ophthalmoscopy, spectral domain optical coherence tomography (OCT), digital fundus photography and, if indicated, angiography.

Note that all adverse events (AEs) must be monitored and recorded in the eCRF from the time the patient consents to any protocol-specific procedure (see Section 8 for further details).

6.1.3 **Evaluations within two weeks (14 days)**

The following must be performed **within the two weeks before** the patient receives the first dose:

Clinical disease measurements: If applicable (i.e. patients with clinically assessable disease);
Complete physical examination: Cardiovascular, respiratory, abdominal, central and peripheral nervous system, dermatological and any other system which might be relevant to the patient's medical history;
Vital signs: Height, weight, ECOG performance status, temperature, blood pressure (BP) and pulse rate;
Electrocardiogram (ECG): Resting 12-lead ECG, conducted in triplicate taken up to 5 minutes apart, with manual calculation of QT interval (QTc**F**);
Laboratory tests (blood/urine samples) to confirm eligibility:

- Hematology – hemoglobin (Hb), white blood cells (WBC) with differential count (neutrophils and lymphocytes), platelets, and D-dimer assay;
- Biochemistry – sodium, potassium, adjusted calcium, phosphate, urea, creatinine, total protein, albumin, bilirubin, alkaline phosphatase (alk phos), alanine aminotransferase (ALT) and aspartate aminotransferase (AST), gamma- glutamyltranspeptide (GGT) and creatine phosphokinase (CPK);
- Urinalysis – pH, specific gravity, glucose, protein and blood.

Serum or urine human chorionic gonadotropin (HCG) test to rule out pregnancy at trial entry; results must be obtained and reviewed before the first

dose of the study medications are administered, if applicable (i.e. women of child bearing potential). See Section 8.4.7 for further details;

Collection of blood sample for PD analysis: to be collected within 28 days before first dose (see Section 6.6.2.1);

Blood sample for circulating free DNA (ctDNA) analysis (as detailed in Section 6.6.2.2).

6.2 Evaluations during the trial

Patients will be followed up weekly during Cycles 1 and 2, then seen on Day 1 of each cycle thereafter.

*** There will be visit windows of +/- 2 days in Cycle 1, or on Day 1 of any cycle. The cycle length must always remain 28 +/- 2 days.**

Physical examination: Physical examination will be performed on the first day of each cycle before VS-6766 and Defactinib administration;

Vitals: ECOG performance status, temperature, pulse rate and BP must be repeated at each visit, before the administration of VS-6766 and Defactinib. Bodyweight should be taken on Day 1 of every cycle;

Adverse events and concomitant treatments: At each visit, before VS-6766 and Defactinib administration, an assessment of any AE experienced since the previous visit must be made by the Investigator or Clinical Research Coordinator. The start and stop dates of the AE together with the relationship of the event to treatment with VS-6766 and Defactinib (causality assessment) must be recorded in the medical records. All AE severity must be graded according to NCI CTCAE Version 5.0. Seriousness of the AE, actions taken regarding each study drug, and outcome should also be collected. Any concomitant treatment must also be recorded in the medical records and in the eCRF. (See Section 8 for further details regarding AE reporting requirements);

Laboratory tests:

Hematology and biochemistry: must be repeated weekly during cycles 1 and 2 and every 4 weeks (i.e. prior to dosing on Day 1 of each cycle) thereafter. Hematology and Biochemistry may be performed the day prior to dosing. If on a day of dosing, hematology, biochemistry and urinalysis should be before VS-6766 and VS-6063 administration.

- Hematology: detailed in Section 6.1.3;
- Biochemistry: detailed in Section 6.1.3;
- Urinalysis: repeated pre-dose on Day 1 of each cycle, as detailed in Section 6.1.3.

Electrocardiogram (ECG): resting 12-lead ECG, conducted in triplicate taken

up to 5 minutes apart, with manual calculation of QT interval (QTc**F**); to be performed on Cycle 2 Day 1, and within 28 days after the last dose of the study drugs and as clinically indicated.

Serum or urine human chorionic gonadotropin (HCG) test to rule out pregnancy; to be performed every 4 weeks if applicable (i.e. women of child bearing potential). See Section 8.4.7 for further details.

Ophthalmological examination: This will be repeated at the end of Cycle 1 (between Cycle 1 Day 22 and pre-dose Cycle 2 Day 1), at the end of Cycle 2 (between Cycle 2 Day 22 and pre-dose Cycle 3 Day 1), at the end of Cycle 4 (between Cycle 4 Day 22 and pre-dose on Cycle 5 Day 1) and every 3 cycles thereafter and within 28 days after the last dose of the study drugs. The result must be available prior to dosing. Interruption and/or reduction of dosing is recommended for management of eye toxicity. Any abnormality observed by an ophthalmologic examination as well as symptoms should be carefully monitored until improved. Guidelines from a specialized ophthalmologist can be provided if requested by the Investigator. Further ophthalmological examinations may be performed in addition to these if clinically indicated. All ophthalmological examinations must be performed and interpreted by a qualified ophthalmologist. Examination detailed in Section 6.1.2;

Assessment of disease: This must be repeated every 2 cycle(s)/8 weeks of continuous treatment, i.e. between C2D22-C3D1, C4D22-C5D1, C6D22-C7D1 etc. (Refer to Section 7). For patients who continue on treatment after six cycles the frequency may be reduced;

Tumor biopsy: Tumor biopsies will be collected from patients **(mandatory) if clinically feasible**. The second biopsy should be taken 2-6 hours after VS-6766 is taken, on day 1-21 of cycle 2 or 3 (see Section 6.6.2.1);

Collection of blood sample for PD analysis: to be collected on C3D1 prior to taking the combination dosing of VS-6766 and Defactinib (pre-Cycle 3, see Section 6.6.2.1);

Blood sample for circulating free DNA analysis: will be taken at the end of every 2 cycles for up to 6 cycles as per Section 6.6.2.2, i.e. C3D1 & C5D1.

6.3 Evaluations at 'off-study' visit

Evaluations at the 'off-study' visit must be performed within 28 days after the last dose of VS-6766 and Defactinib. The following investigations must be done:

Physical examination including ECOG performance status, temperature, pulse rate, BP and bodyweight;

Hematology tests: detailed in Section 6.1.3;

Biochemistry tests: detailed in Section 6.1.3;

Urinalysis: detailed in Section 6.1.3;

Serum or urine human chorionic gonadotropin (HCG) test if applicable (i.e. women of child bearing potential).
ECG: detailed in Section 6.1.2;
Ophthalmological exam: detailed in Section 6.1.2;
Tumor biopsy (third biopsy sample): detailed in Section 6.6.2.1;
Blood sample for PD analysis: detailed in Section 6.6.2.1;
Blood sample for circulating free DNA analysis: detailed in Section 6.6.2.2;
Assessment of tumor disease, unless assessment has been performed within the previous four weeks (28 days);
Assessment of AEs (also see Section 8); and
Assessment of concomitant treatments.

6.4 Follow-up

Post-treatment, patients will be followed for survival until their death or up to 5 years after the last patient is enrolled. All AEs regardless of causality that are still present 28 days after the last administration of VS-6766 and/or Defactinib will be followed monthly until resolution (grade 1 or less) or stabilization. Adverse event evaluation and abnormal laboratory values will be monitored during the monthly follow up visits. Survival information will be collected via telephone or clinical record review. The date and cause of death will be recorded; the cause of death will be categorized as either cancer-related or cancer-unrelated. Patients will be considered “lost to follow-up” after 3 unsuccessful contact attempts by telephone (attempts occurring within 2 years).

6.5 Withdrawal Visit/Discontinuation of Therapy

The Investigator must make every reasonable effort to keep each patient on trial for the whole duration of the trial (i.e. until 28 days after last combination therapy administration). However, if the Investigator removes a patient from the trial or if the patient declines further participation, final ‘off-study’ assessments should be performed before any therapeutic intervention. All the results of the evaluations and observations, together with a description of the reasons for withdrawal from the trial, must be recorded in the medical records and in the eCRF.

Patients who are removed from the trial due to adverse events (clinical or laboratory) will be treated and followed according to accepted medical practice. All pertinent information concerning the outcome of such treatment must be recorded in the eCRF and on the serious adverse event (SAE) report form where necessary.

The following are justifiable reasons for the Investigator to withdraw a patient from trial.

- Unacceptable toxicity (AE/SAE)
- Withdrawal of consent
- Serious violation of the trial protocol (including persistent patient attendance failure and persistent non-compliance)
- Sponsor's decision to terminate the trial
- Withdrawal by the Investigator for clinical reasons not related to the study medications
- Evidence of disease progression. If patients have mild progression (20-30% increase in the sum of the longest diameters from the baseline), and meet criteria listed in section 3.3, and if Investigators conclude that patients have had clinical benefit from the study medications, the study treatment will be allowed to continue for 2 more cycles as long as patients tolerate the study medications without grade 3 or higher toxicity. If further progression is confirmed with follow-up imaging studies, patients will be removed from the study.
- Symptomatic deterioration
- Pregnancy

Patients that are discontinued from the study are still evaluable as defined in section 3.3.

6.6 Laboratory Procedures/Evaluations

6.6.1 Clinical Laboratory Evaluations

Laboratory tests (blood/urine samples) performed in our trial include:

Hematology – hemoglobin (Hb), white blood cells (WBC) with differential count (neutrophils and lymphocytes), platelets, PT/INR, PTT, and D-dimer assay;

- PT/INR and PTT required for inclusion criteria and subsequently prior to each biopsy as per institutional standard practice (at minimum within 7 days prior to biopsy).

Biochemistry – sodium, potassium, adjusted calcium, phosphate, urea, creatinine, total protein, albumin, bilirubin, alkaline phosphatase (alk phos), alanine aminotransferase (ALT) and aspartate aminotransferase (AST), gamma- glutamyltranspeptide (GGT) and creatine phosphokinase (CPK);
Urinalysis – pH, specific gravity, glucose, protein and blood.
Serum or urine human chorionic gonadotropin (HCG) test

6.6.2 Special Assays or Procedures

6.6.2.1 *Pharmacodynamic assays*

Tumor Biopsies

Patients will be consented for collection of three mandatory pre- and post-treatment tumor biopsies. These should be collected at the following time points as long as clinically feasible:

- Baseline (within 28 days before first dose);
- On-treatment (after Cycle 1 or Cycle 2);
- Post-treatment (within 28 days after last dose).

As the fasting requirements of VS-6766 and Defactinib (VS-6063) differ, the drugs cannot be taken at the same time. Therefore, the second biopsy following combination dosing should be scheduled from the time of VS-6766 dose. The second biopsy should be scheduled for 2-6 hours after a dose of VS-6766 is taken in Cycle 2 or 3. The third biopsy should also if possible be taken 2-6 hours after a dose of VS-6766 is taken, before the patient stops drug.

Samples of tumor will undergo analysis for possible changes in tumor metabolic activity, signaling to the ERK, YAP, FAK, and PI3K-TOR pathways, changes in UM cell proliferation (Ki67) and apoptosis induction (caspase activation), and changes in tumor microenvironment. The resistance mechanism of this combination treatment will also be investigated. The specific type and number of biomarkers from the tumor samples will be decided during the course of the study and documented in the study records.

Core needle tumor biopsies will be taken according to local procedures. Within 28 days of the first dose, tumor biopsies will be divided into 3 samples, one to be immediately snap frozen in liquid nitrogen or a dry ice bath for PD analysis, one to be formalin fixed and embedded in paraffin (FFPE) as per local procedures for PD biomarker analysis, and one to establish PDX models. If clinically feasible, additional 1 core biopsy specimen will be obtained during the first biopsy for NGS analysis on oncogenic mutations and amplifications.

For the second biopsy (on-treatment), if the biopsy will be performed after cycle 1 the tumor biopsies will be divided into two samples, one to be immediately snap frozen in liquid nitrogen or a dry ice bath for PD analysis and one to be formalin fixed and embedded in paraffin (FFPE) as per local procedures for PD biomarker analysis. If the biopsy will be performed after cycle 2 the tumor biopsies will be divided into three samples, one to be immediately snap frozen in liquid nitrogen or a dry ice bath for PD

analysis, one to be formalin fixed and embedded in paraffin (FFPE) as per local procedures for PD biomarker analysis, and one to establish PDX models.

Where there is limited tissue to biopsy, priority should be given to securing a FFPE sample over a snap frozen sample.

Tumor biopsies will be analyzed at the laboratories of Dr. Takami Sato and Dr. Andrew Aplin (Thomas Jefferson University/Sidney Kimmel Cancer Center – 1015 Walnut St. Philadelphia, PA 19107), Dr. Gutkind (University of California-San Diego Moores Cancer Center – 3855 Health Sciences Dr. La Jolla, CA 92037), and other external laboratories that utilize techniques required for our study. Please refer to the Study Laboratory Standard Operating Procedures for handling, storage and shipment of samples.

Blood Samples
PD blood samples (30ml) will be collected:

- Baseline (within 14 days before first dose);
- Pre-Cycle 3 (on C3D1 prior to combination dosing of VS-6766 and Defactinib);
- Progression (within 28 days after last dose).

PD blood samples will be analyzed at the aforementioned laboratories (Drs. Sato & Aplin, Dr. Gutkind, external laboratory). Please refer to the Study Laboratory Standard Operating Procedures for handling, storage and shipment of samples.

6.6.2.2 ***Predictive biomarker assays***

Analysis of circulating free DNA
Circulating free tumor DNA in plasma will be sequenced for genetic mutations according to agreed SOPs and validated methods.

20ml of plasma will be collected for all patients at baseline (within 14 days of first dose), at the end of every 2 cycles for up to 6 cycles and at progression. This is for the purpose of studying circulating free DNA and correlating it to response.

Samples will be analyzed at the aforementioned laboratories (Drs. Sato & Aplin, Dr. Gutkind, external laboratory). Please refer to the Study Laboratory Standard Operating Procedures for handling, storage and shipment of samples.

7 Evaluation of Efficacy

Disease must be measured according to the RECIST criteria (version 1.1) given in Appendix B. These criteria are based on the revised guidelines by Eisenhauer et al. (42, 43).

7.1 Measurement of Effects

7.1.1 Measurable and Non-Measurable Disease

Measurable Disease

Measurable disease is defined by the presence of at least one measurable lesion as defined below and adapted from the RECIST criteria version 1.1 (Appendix B). All measurements must be recorded in metric notation.

The longest diameter (LD) of the largest lesion should be ≥ 10 mm by CT or MRI in order to become eligible for the study as previously mentioned.

Since some melanoma metastases contain melanin pigment and since successfully treated liver metastases may develop significant necrosis/hemorrhage, evaluation of radiological response after embolization treatments is generally difficult. Therefore, we will obtain an MRI of the liver, preferably with **gadoxetate** disodium (Eovist) contrast study for the precise evaluation of radiological responses in liver metastasis. If MRI is not available, triphasic CT of the abdomen will be obtained. However, the same modality will be used for measurement of the liver metastases and assessment of treatment response. The hepatobiliary phase images of MRI with **gadoxetate** disodium (Eovist) contrast is preferred for tumor response assessment due to its superiority in measurement of tumor size.

Non-measurable disease

All other lesions including small lesions (<10 mm on spiral CT scans or MRI) are considered “non-measurable”. The following lesions are also considered as “non-measurable”: Bone lesions, leptomeningeal disease, ascites, pleural/pericardial effusion, abdominal masses that are not confirmed and followed by imaging techniques, tumors treated by radiation, ablation or surgical resection.

7.1.2 Baseline documentation of “Target” and “Non-Target” Lesions

Target Lesions (Indicator Lesions)

The target lesions are defined as measurable lesions that will be used for evaluation of response. For this protocol, target lesions require serial measurement of a longest diameter and should be ≥10 mm by triphasic CT or MRI at the time of study entry.

Previously irradiated lesions can be taken into account as measurable disease when they are clearly progressing at the time of initiating systemic therapy.

Lymph nodes that have a short axis of <10mm are considered non-pathological and should not be recorded or followed. Pathological lymph nodes with <15mm but >10mm short axis are considered non measurable. Pathological lymph nodes with 15mm short axis are considered measurable and can be selected as target lesions, however lymph nodes should not be selected as target lesions when other suitable target lesions are available.

Target lesions (up to a maximum number of 2 lesions per organs) must be identified and measured at baseline prior to the initiation of treatment. Target lesions must be selected on the basis of their size (LD) and suitability for repetitive measurements.

Non-Target Lesions
All other lesions (or sites of melanoma) must be identified as “non-target” and their location and characteristics must be recorded at baseline. During follow-up evaluations, these lesions must be followed as “present” or “absent”.

7.2 Timing of tumor assessments

7.2.1 Baseline evaluations

These must include radiological measurements and as indicated chest, abdominal and pelvic computerized tomography (CT) scan, magnetic resonance imaging (MRI) and/or clinical measurements as appropriate. All areas of disease present must be documented (even if specific lesions are not going to be followed for response). Any non-measurable lesions must be stated as being present. For clinical measurements, documentation by color photography including a ruler to estimate the size of the lesion is strongly recommended, as this aids external independent review of responses (see Appendix B for RECIST 1.1 criteria).

7.2.2 Evaluations during and at ‘off-study’

Tumor assessments must be repeated every 2 cycle(s)/8 weeks if continuous treatment or more frequently, when clinically indicated. All lesions measured at baseline must be measured at every subsequent disease assessment, and recorded clearly on the scan reports. All non-measurable lesions noted at baseline must be noted on the scan report as present or absent.

All patients, who are removed from the trial for reasons other than progressive disease, must be re-evaluated at the time of treatment discontinuation, unless a tumor assessment was performed within the previous four weeks.

It is the responsibility of the Principal Investigator to ensure that the radiologists are aware of the requirement to follow-up and measure every target lesion mentioned at baseline and comment on the non-target lesions in accordance with RECIST 1.1 criteria.

7.3 Evaluation of overall response of metastasis

The investigators must identify target indicator lesions and measure them within 4 weeks prior to the first treatment as baseline. The investigators will then measure the same target lesions after each treatment cycle.

New lesions will be defined as: A lesion equal or greater than 10 mm in longest diameter or a lymph node equal or greater than 15 mm in short axis is considered to be a new lesion if the lesion is not previously detected or less than 0.5 cm in baseline imaging.

The sum of the baseline LDs will be compared to the sum of LDs after treatment.

7.4 Response Criteria for metastases

The response of metastases will be evaluated by MRI or CT using the criteria adapted from the international criteria proposed by the Response Evaluation Criteria in Solid Tumors (RECIST) Committee. The same imaging modality must be used for the serial measurements of tumor diameter. The definition of response of metastases is shown in Table 2. Evaluation of target and non-target lesions to determine response of metastases is shown in Table 3. The best response during study treatment will be used for efficacy analysis.

To be assigned a status of complete response (CR) or partial response (PR), changes in tumor measurements must be confirmed by repeat measurements performed no less than four weeks after the response criteria are met. To be assigned a status of stable disease (SD), follow-up measurements must have met the SD criteria at least once and at least six weeks after the initial dose of the investigational medicinal product VS-6766 and Defactinib (VS-6063) is given.

Tumor response should be classified as “not evaluable” (NE), only when it is not possible to classify it under another response category, for example, when baseline and/or follow-up assessment is not performed or not performed appropriately.

Table 2. The definition of response of metastases

Complete Response (CR)	Disappearance of all target and non-target lesions
Partial Response (PR)	>30% decrease in the sum of the longest diameters ("sum LD") relative to baseline sum LD with at least stable non-target lesions
Stable Disease (SD)	Absence of change which would qualify as response or progression
Progression (PD)	>20% increase in the sum LD in target lesions or unequivocal progression of non-target lesions<br>Appearance of one or more new lesions >10 mm

Table 3. Evaluation of Target and Non-Target Lesions

Target Lesions	Non-Target Lesions	New Lesions	Overall Response
CR	CR	No	CR
CR	SD	No	PR
PR	Non-PD	No	PR
SD	Non-PD	No	SD
PD	Any	Yes or No	PD
Any	PD	Yes or No	PD
Any	Any	Yes	PD

7.4.1 Recording of response in eCRF

The applicable overall response category for each visit that includes disease assessment must be recorded in the eCRF, even though the criteria for determination of a complete response (CR) or partial response (PR) by the protocol must be confirmed after two consecutive observations, no less than four weeks apart.

7.5 Evaluation of progression free survival (PFS)

PFS is measured from the start of the first study treatment to confirmation of progression of disease by either imaging tests or death of patient, whichever comes first. Patients without progression of metastasis at the last follow-up visit will be censored.

7.6 Evaluation of overall survival (OS)

Overall survival (OS) is measured from the first treatment to patient death. Date and cause of death will be recorded. The cause of death will be categorized as either cancer-related or cancer-unrelated. Patients who are alive at the last follow-up visit will be censored.

8 Evaluation of Safety

8.1 Specification of Safety Parameters

8.1.1 Unanticipated Problems

Unanticipated problems (UAPs) include, in general, any incident, experience, or outcome that meets the following criteria:

- unexpected in terms of nature, severity, or frequency given (a) the research procedures that are described in the protocol-related documents, such as the IRB-approved research protocol and informed consent document; and (b) the characteristics of the participant population being studied;

UAPs are considered to pose risk to participants or others when they suggest that the research places participants or others at a greater risk of harm (including physical, psychological, economic, or social harm) than was previously known or recognized.

8.1.2 Adverse Events

An adverse event is any untoward or unfavorable medical occurrence in a human participant, including any abnormal sign (for example, abnormal physical exam or laboratory finding), symptom, or disease, temporally associated with the participant’s

participation in the research, whether or not considered related to the participant's participation in the research.

8.1.3 Serious Adverse Events

A serious adverse event (SAE) is one that meets one or more of the following criteria:

- Results in death
- Is life-threatening (places the participant at immediate risk of death from the event as it occurred)
- Is disabling or incapacitating
- Results in inpatient hospitalization or prolongation of existing hospitalization
- Results in a persistent or significant disability or incapacity
- Results in a congenital anomaly or birth defect

An important medical event that may not result in death, be life threatening, or require hospitalization may be considered an SAE when, based upon appropriate medical judgment, the event may jeopardize the participant or may require intervention to prevent one of the outcomes listed in this definition.

8.2 Safety Assessment and Follow-Up

For eligible patients, serious adverse event (SAE) and adverse event (AE) collection and monitoring commences from the time the patient gives their written consent to participate in the trial and continues for 28 days after the last administration of VS-6766 and Defactinib (VS-6063).

Follow-up of all AEs will be performed regardless of causality until the events resolve, stabilize or the patient starts another anti-cancer therapy.

8.3 Recording Adverse Events

The following subsections detail what information must be documented for each adverse event occurring during the time period specified in Section 8.2 Safety Assessment and Follow-Up.

8.3.1 Relationship to Study Intervention

The relationship to study intervention or study participation must be assessed and documented for all adverse events. Evaluation of relatedness must consider etiologies such as natural history of the underlying disease, concurrent illness, concomitant therapy, study-related procedures, accidents, and other external factors.

The following guidelines are used to assess relationship of an event to study intervention:

1. Related (Possible, Probable, Definite)
    a. The event is known to occur with the study intervention.
    b. There is a temporal relationship between the intervention and event onset.
    c. The event abates when the intervention is discontinued.
    d. The event reappears upon a re-challenge with the intervention.
2. Not Related (Unlikely, Not Related)
    a. There is no temporal relationship between the intervention and event onset.
    b. An alternate etiology has been established.

8.3.2 Expectedness

The PI is responsible for determining whether an AE is expected or unexpected. An AE will be considered unexpected if the nature, severity, or frequency of the event is not consistent with the risk information previously described for the intervention. Risk information to assess expectedness can be obtained from preclinical studies, the investigator's brochure, published medical literature, the protocol, or the informed consent document.

8.3.3 Severity of Event

Adverse events will be graded for severity according to the Common Terminology Criteria for Adverse Events (CTCAE) version 5.0.

8.3.4 Intervention

Any intervention implemented to treat the adverse event must be documented for all adverse events.

8.4 Safety Reporting

The sponsor-investigator shall be responsible for all required periodic safety updates to the Regulatory Authorities and expedited reporting of all SAEs occurring during the performance of the Study, for initial and all subsequent follow-up reports, in accordance with local and regional regulations.

Adverse events (AE) will be reported to the Thomas Jefferson University IRB and the Data Safety Monitoring Board (DMSB) via Clinical Research Management Office (CRMO) as specified in the TJU Data Safety and Monitoring Plan (Refer to Table). All AEs must be graded according to NCI CTCAE Version 5.0

8.4.1 Reporting to IRB

8.4.1.1 *Unanticipated Problems*

All incidents or events that meet criteria for unanticipated problems (UAPs) as defined in Section 8.1.1 Unanticipated Problems require the creation and completion of an unanticipated problem report form (OHR-20).

UAPs that pose risk to participants or others, and that are not AEs, will be submitted to the IRB on an OHR-20 form via the eazUP system within 10 working days of the investigator becoming aware of the event.

UAPs that do not pose risk to participants or others will be submitted to the IRB at the next continuing review.

8.4.1.2 *Adverse Events*

Grade 1 AEs will be reported to the IRB at continuing review.

Grade 2 AEs will be reported to the IRB at the time of continuing review.

8.4.1.3 *Serious Adverse Events*

SAEs will be reported to the IRB on OHR-10 forms via the electronic reporting system (eSAEy) according to the required time frames described below.

Grade 3-4 AEs that are unexpected and deemed to be at least possibly related to the study will be reported to the IRB within 2 working days of knowledge of the event.

Grade 3-4 AEs that are deemed unrelated to the study will be reported to the IRB within 5 working days.

Grade 5 AEs will be reported to the IRB within one working day of knowledge of the event.

All SAEs will be submitted to the IRB at continuing review, including those that were reported previously.

8.4.1.4 *Events exempt from being reported as SAEs*

Events specified in this section do not require reporting as SAEs in this trial, unless hospitalization is prolonged for any reason and then an SAE form must be completed. The events must still be recorded in the appropriate section of the case report form (CRF).

Screening period – SAEs occurring in the period prior to administration of VS-6766 and Defactinib (VS- 6063) from the time of informed consent to the day prior to cycle 1 day 1 of VS-6766 and VS-6063 administration that are not related to the design or conduct of the trial are exempt from reporting.

Elective admissions – Elective admissions to hospital for procedures which were planned and documented in the medical records at the time of consent are not SAEs, and do not require SAE reporting. Hospitalization for administration of the study medications according to the trial protocol is also exempt from being reported as an SAE, unless the hospitalization is prolonged by any reason and then an SAE must be reported.

Death due to disease progression – all cases of death during the study period up to 28 days after the trial should be reported to Verastem. After the 28 day period, cases of death due to disease progression do not require reporting unless considered related to study medications.

8.4.1.5 *Follow-up of adverse events*

Follow-up will continue until all the necessary safety data for the event has been gathered and any AE or SAE regardless of causality has either resolved, returned to baseline or stabilized.

8.4.2 Reporting to SKCC DSMC

All AEs and SAEs, safety and toxicity data, and any corrective actions will be submitted to the DSMC per the frequency described in the SKCC DSMP. The report to the SKCC DSMC will also include any unanticipated problems that in the opinion of the PI should be reported to the DSMC.

For expedited reporting requirements, see table below:

DSMC AE/SAE Reporting Requirements

	Grade 1	Grade 2		Grade 3				Grades 4 and 5
				Unexpected		Expected		
	Unexpected and Expected	Unexpected	Expected	With Hospitalization	Without Hospitalization	With Hospitalization	Without Hospitalization	Unexpected and Expected
Unrelated Unlikely	Reviewed at Quarterly DSMC Meeting and IRB Annual Review	Reviewed at Quarterly DSMC Meeting and IRB Annual Review	Reviewed at Quarterly DSMC Meeting and IRB Annual Review	5 Working Days	Reviewed at Quarterly DSMC Meeting and IRB Annual Review	5 Working Days	Reviewed at Quarterly DSMC Meeting and IRB Annual Review	Phase I - 48 Hours<br>(Death: 24 Hours)<br>Phase II - 5 working days
Possible Probably Definite	Reviewed at Quarterly DSMC Meeting and IRB Annual Review	Reviewed at Quarterly DSMC Meeting and IRB Annual Review	Reviewed at Quarterly DSMC Meeting and IRB Annual Review	48 Hours (Death: 24 Hours)	Phase I - 48 Hours<br>Phase II - 5 working days	48 Hours (Death: 24 Hours)	Reviewed at Quarterly DSMC Meeting and IRB Annual Review	Phase I and Phase II - 48 Hours<br>(Death: 24 Hours)

8.4.3 Reporting of serious adverse events to Verastem Oncology

All initial and follow-up SAEs and Safety Information including pregnancy reports must be reported to Verastem, Inc. Safety and Pharmacovigilance in English at drugsafety@verastem.commailto: or via fax at 1-781-465-7936 within 24 hours of knowledge of the event concerning the Verastem, Inc. product.

Copies of any correspondence or telephone conversation logs with the applicable Regulatory Authorities regarding all SAE(s), irrespective of association with the Study

Drug(s), within a reasonable time frame must be provided to Verastem, Inc. Safety and Pharmacovigilance.

The Principal Investigator will report such SAEs using an FDA MEDWATCH form and the Serious Adverse Event Fax Cover Sheet provided by Verastem Oncology. The event term(s) being reported, start and stop dates of the events, outcome of the events, toxicity grade for the events, assessment of causality for the events, action taken with the study drugs for the event will all be reported. SAEs should be reported as soon as they are determined to meet the definition, even if complete information is not yet available.

a. *Follow-Up Information*. Institutions will assist Verastem Oncology in investigating any SAE and will provide any follow-up information reasonably requested by Verastem Oncology.
b. *Regulatory Reporting.* Reporting an SAE to Verastem Oncology does not relieve Institutions of responsibility for reporting it to the FDA, as required.

8.4.4 **Reporting to FDA**

SAEs that are related, unexpected, fatal, or life-threatening are reportable through the Food and Drug Administration (FDA) MedWatch program by telephone or fax no later than 7 calendar days after initial receipt of the information. Further information on the timing of submissions are as directed by FDA guidelines (http://www.fda.gov/medwatch/index.html). Serious, unexpected events that suggest significant clinical risk will be submitted to within 15 calendar days after initial receipt of this information.

Food and Drug Administration:
Telephone 1-800-FDA-1088
Fax 1-332-FDA-0178
http://www.fda.gov/medwatch/report.htm

8.4.5 **Sponsor reporting responsibilities**

1. Adverse events which meet all of the following criteria must be reported to all participating institutions for IRB submission within 2 weeks of notification of the event.
    a. Unexpected (in terms of nature, severity, or frequency) given
        i. the research procedures that are described in the protocol-related documents, such as the IRB-approved research protocol and informed consent document; and
        ii. the characteristics of the subject population being studied;

   b. Possibly related to participation in the research (possibly related means there is a reasonable possibility that the incident, experience, or outcome may have been caused by the procedures involved in the research); and
   c. Serious (refer to above definition) or otherwise one that suggests that the research places subjects or others at a greater risk of physical or psychological harm than was previously known or recognized.
2. If the adverse event requires modification of the study protocol and informed consent Verastem will be informed. If these changes on the protocol are finalized, they will be provided to all participating institutions in the form of an amendment from the OCR for each site's IRB of record along with the report of the adverse event.
3. Copies of all related correspondence and reporting documents will be maintained in a centralized regulatory file for this study at OCR.
4. SAEs that are related, unexpected, fatal, or life-threatening are reportable through the Food and Drug Administration (FDA) MedWatch program by telephone or fax no later than 7 calendar days after initial receipt of the information. Further information on the timing of submissions are as directed by FDA guidelines (http://www.fda.gov/medwatch/index.html). Serious, unexpected events that suggest significant clinical risk will be submitted to within 15 calendar days after initial receipt of this information.

8.4.6 Recording of AE and SAEs in eCRFs

All AEs, including SAEs, must be recorded in the eCRF for eligible patients. All concomitant medications, including herbal medications and supplements must be recorded. Any therapy used to treat the event must be recorded. The eCRF will be reconciled with the safety database during and at the end of the trial. Therefore, the sites should ensure the data entered on the SAE report form and the data entered into the eCRF are consistent. The Safety Review Committee and the Investigator(s) will regularly review the safety data from both the safety and the clinical database.

Monthly, the sponsor-investigator site will reconcile a listing of SAEs or Safety Information provided by Verastem, Inc. with the site's records. Discrepancies between the Verastem, Inc. and sponsor-investigator site's records will be investigated by the sponsor-investigator site. The sponsor-investigator will send an updated AE form, MedWatch or CIOMS to Verastem, Inc. or designee to resolve identified discrepancies.

8.4.7 Reporting of Pregnancy

The effects of Defactinib (VS-6063) and VS-6766 on conception, pregnancy, and lactation are unknown.

At Screening, all male and female subjects of reproductive potential (i.e., not surgically sterile or female subjects who are not postmenopausal) must be willing to use **highly effective** methods of contraception for the duration of the study intervention and for 3 months after the last dose of Defactinib and VS-6766. Male subjects must also refrain from donating sperm during their participation in the study and for at least 3 months after the last dose of Defactinib and VS-6766. Pregnancy testing will be performed throughout the study and 28 days after the last dose.

A subject must immediately inform the Investigator if the subject or subject's partner becomes pregnant from the time of consent to 30 days after the last dose of study drug(s). Any female subjects receiving study drug(s) who become pregnant must immediately and permanently discontinue study drug(s). The Investigator should counsel the subject, discussing any risks of continuing the pregnancy and any possible effects on the fetus. The pregnant woman should be referred to an obstetrician/gynecologist experienced in reproductive toxicity for further evaluation and counseling.

Although pregnancy itself is not regarded as an AE unless there is cause to believe that the study intervention may have interfered with the effectiveness of contraceptive medication or if the outcome of pregnancy meets SAE criteria (miscarriage or congenital anomaly/birth defect, etc.), in which case it should be reported in the same manner and timelines as an SAE, the outcome will need to be documented.

Any SAEs associated with pregnancy (e.g. congenital abnormalities/birth defects/spontaneous miscarriage or any other serious events) must additionally be reported. In addition, any infant death or congenital anomaly occurring after 30 days that the Investigator suspects is related to the in-utero exposure to the study intervention should also be reported as an SAE. Hospitalization for normal delivery of a healthy newborn is not an SAE.

Consent to provide follow-up information regarding the outcome of the pregnancy and the health of the infant until 30 days old will be requested.

Any pregnancy occurring in a patient or a patient's partner during treatment with a study medication or occurring within six months of last study medication administration must be reported to the DSMB and Verastem, Inc. within 24 hours of the Investigator's knowledge of the pregnancy using a Pregnancy Report Form. The Investigator will observe the pregnant woman until completion of the pregnancy and must notify Verastem, Inc. of the outcome within 24 hours of the Investigator's knowledge of the pregnancy outcome using a Pregnancy Outcome Form. This notification includes pregnancies resulting in live, normal births.

Highly Effective Methods of Contraception are methods that can achieve a failure rate of less than 1% per year when used consistently and correctly are considered as highly effective birth control methods. Such methods include:

- combined (estrogen and progestogen containing) hormonal contraception associated with inhibition of ovulation[1]:
    - oral
    - intravaginal
    - transdermal
- progestogen-only hormonal contraception associated with inhibition of ovulation[1]:
    - oral
    - injectable
    - implantable[2]
- intrauterine device (IUD)
- intrauterine hormone-releasing system[2]
- bilateral tubal occlusion[2]
- vasectomized partner[2,3]
- sexual abstinence[4]

[1]Hormonal contraception may be susceptible to interaction with the IMP, which may reduce the efficacy of the contraception method.

[2]Contraception methods that are considered to have low user dependency.

[3]Vasectomized partner is a highly effective birth control method provided that partner is the sole sexual partner of the study participant and that the vasectomized partner has received medical assessment of the surgical success.

[4]Sexual abstinence is considered a highly effective method only if defined as refraining from heterosexual intercourse during the entire period of risk associated with the study treatments. The reliability of sexual abstinence needs to be evaluated in relation to the duration of the clinical study and the preferred and usual lifestyle of the patient.

8.5 **Halting Rules**

Adverse events which meet all of the following criteria must be reported to all participating institutions for IRB submission within 2 weeks of notification of the event.

- Unexpected (in terms of nature, severity, or frequency) given

        - the research procedures that are described in the protocol-related documents, such as the IRB-approved research protocol and informed consent document; and
        - the characteristics of the subject population being studied.
    - Possibly related to participation in the research (possibly related means there is a reasonable possibility that the incident, experience, or outcome may have been caused by the procedures involved in the research); and
    - Serious (refer to above definition) or otherwise one that suggests that the research places subjects or others at a greater risk of physical or psychological harm than was previously known or recognized.

This may prompt temporary suspension of enrollment and/or study interventions until a safety review is convened (either routine or ad hoc). The objective of the safety review is to decide whether the study (or intervention for an individual or study cohort) should continue per protocol, proceed with caution, be further investigated, be discontinued, or be modified and then proceed. Suspension of enrollment (for a particular group, a particular study site or for the entire study) is a potential outcome of a safety review.

Subsequent review of serious, unexpected, and related AEs by the Medical Monitor, DSMC, IRB, the sponsor(s), or the FDA or relevant local regulatory authorities may also result in suspension of further trial interventions/administration of study product at a site. The FDA and study sponsor(s) retain the authority to suspend additional enrollment and study interventions/administration of study product for the entire study, as applicable.

Urgent safety measures
The Sponsor or Investigator may take appropriate urgent safety measures (USMs) in order to protect the patient of a clinical trial against any immediate hazard to their health or safety. This includes procedures taken to protect patients from pandemics or infections that pose serious risk to human health.

USMs may be taken without prior authorization from the competent authority.

The Food and Drug Administration (FDA) and the Research Ethics Committee (REC) must be notified within three days of such measures being taken.

9 Study Oversight

In addition to the PI's responsibility for oversight, study oversight will be under the direction of the SKCC's Data and Safety Monitoring Committee (DSMC). The SKCC DSMC operates in compliance with a Data and Safety Monitoring Plan (DSMP) that is approved by the NCI.

10 Clinical Site Monitoring and Auditing

Clinical site monitoring and auditing is conducted to ensure that the rights of human participants are protected, that the study is implemented in accordance with the protocol and/or other operating procedures, and that the quality and integrity of study data and data collection methods are maintained. Monitoring and auditing for this study will be performed in accordance with the SKCC's Data and Safety Monitoring Plan (DSMP) developed by the SKCC Data and Safety Monitoring Committee (DSMC). The DSMP specifies the frequency of monitoring, monitoring procedures, the level of clinical site monitoring activities (e.g., the percentage of participant data to be reviewed), and the distribution of monitoring reports. Some monitoring activities may be performed remotely, while others will take place at the study site(s). Appropriate staff will conduct monitoring activities and provide reports of the findings and associated action items in accordance with the details described in the SKCC DSMP.

11 Statistical Considerations

10.1 Study Hypotheses

The primary objective null hypothesis is that the true response rate is 20%, and the alternative hypothesis is that the true response rate is 50%.

10.2 Analysis Plans

In order to further characterize the efficacy of the combination of VS-6766 and Defactinib (VS-6063) in metastatic uveal melanoma patients, Simon's Optimal two-stage design will be used for conducting this clinical trial (44). The best response during study treatment in individual patients will be used for efficacy analysis.

The null hypothesis is that the true response rate is 20%, and the alternative hypothesis is that the true response rate is 50%. The Simon's Optimal two-stage design was selected with a maximum of 18 patients and target alpha level of 0.05 and power 80%.

The cohort escalation design is guided by a desire to stop the trial early if the actual stabilization rate is 20% or less. No medication is approved for MUM by the US FDA and potential clinical benefit could be obtained if MUM patients achieve stable disease (SD); therefore, we will consider overall Disease Control Rate (DCR) (CR+PR+SD) of 50% as a meaningful endpoint for this early phase clinical study. This is considered to be an appropriate goal since the Overall Response Rates (ORR) (CR+PR) and overall Disease Control Rate (DCR) (CR+PR+SD) of combination treatments using the FDA approved medications ipilimumab and nivolumab are reported to be 11.8-16.7% and 29.4-64%, respectively (15).

We hope that we will have at least 50% overall DCR with reasonable toxicity in this expansion cohort. A total of 18 UM patients with systemic metastasis will be enrolled based on Simon's Optimal two-stage design. If overall DCR is less than 20%, we may not consider further clinical studies with this combination treatment (Table 4).

The trial is carried out in two stages. In stage I, a total number of 8 patients are accrued. If there are 2 or fewer overall responses (CR+PR+SD) among these 8 patients, further enrollment of patients may be stopped with the conclusion that the DCR cannot be 50% or greater. Otherwise, an additional 10 patients will be accrued in stage II, resulting in a total sample size of 18. If there are 7 or more responses among these 18 patients, we reject the null hypothesis and claim that the treatment is promising [Optimal Design].

With this design, we have no more than a 5% chance of concluding effective (>=50% stabilization rate) when the success rate is at most 20%. Similarly, we have no more than a 20% chance of concluding ineffective (<=20% stabilization rate) when it is effective (50% stabilization rate). If the actual response rate is 20% or worse, we have at least a 0.79 probability that the trial will stop after the first 14 subjects. The overall power of this design is 80.0%.

Table 4: Cohort Expansion Stages

Stage	Look after this number of patients	Stop if number of successes is less than or equal to
I	8	2
II	10	6

The final analysis will be conducted after one of the following conditions is met.

- The trial is terminated early (for example, due to toxicity or inefficacy as defined above).
- All patients have had the opportunity to receive 1 cycle of treatment and have completed their 'off-study' visit (i.e. 28 days after the last dose of VS-6766 and/or Defactinib).

Once one of the conditions is met, a data cut-off date will be established. All patient visits occurring on or before this date will be analyzed and summarized in the final clinical study report. Any data collected after this date will be summarized in a supplemental report.

10.3 Interim Analyses and Stopping Rules

10.3.1 Efficacy Review

As described in section 11.2, the trial is carried out in two stages. In stage I, a total number of 8 patients are accrued. If there are 2 or fewer overall responses (CR+PR+SD) among these 8 patients, further enrollment of patients may be stopped with the conclusion that the DCR cannot be 50% or greater. If this happens, the study results will be shared with Verastem Oncology and the future study design will be discussed.

Otherwise, an additional 10 patients will be accrued in stage II, resulting in a total sample size of 18.

10.4 Defining the end of trial

The 'end of trial' is defined as the date when the last patient has completed the 'off-study' visit or the final follow-up visit (whichever is the latter).

It is the responsibility of the Principal Investigator to inform the Food and Drug Administration (FDA) within 90 days of the 'end of the trial' that the trial has closed.

The entire trial will be stopped when:

- The drug is considered too toxic to continue treatment before the required number of patients being recruited.
- The drug is considered ineffective (actual stabilization rate is 20% or less); see section 11.2.
- The stated number of patients to be recruited is reached.
- The stated objectives of the trial are achieved.

Regardless of the reason for termination, all data available for the patient at the time of discontinuation of follow-up must be recorded in the eCRF. All reasons for discontinuation of treatment must be documented.

In terminating the trial, the Investigators must ensure that adequate consideration is given to the protection of the patient's interest.

10.5 Sample Size Considerations

Based on the Simon's Optimal two-stage design, a total number of 8 patients are accrued in stage I. If there are 2 or fewer overall responses (CR+PR+SD) among these 8 patients, further enrollment of patients may be stopped with the conclusion that the DCR cannot be 50% or greater. Otherwise, an additional 10 patients will be accrued in stage II, resulting in a total sample size of 18.

Simon's Optimal two-stage design was selected with a maximum of 18 patients and target alpha level of 0.05 and power 80%. With this design, we have no more than a 5% chance of concluding effective (>=50% stabilization rate) when the success rate is at most 20%. Similarly, we have no more than a 20% chance of concluding ineffective (<=20% stabilization rate) when it is effective (50% stabilization rate). If the actual response rate is 20% or worse, we have at least a 0.79 probability that the trial will stop after the first 14 subjects. The overall power of this design is 80.0%.

10.5.1 Replacement Policy

Patients who do not complete one cycle of dosing with VS-6766 and Defactinib (VS-6063) for reasons other than toxicity will be replaced.

10.5.2 Accrual Estimates

It is expected that the trial recruitment duration will be 6 to 18 months with an expected accrual rate of approximately 1-3 patients per month at Thomas Jefferson University Hospital. A total of up to 18 patients will be entered into this trial, depending on the disease control rate in the first stage of the study.

10.6 Data Analysis

Data will be presented in a descriptive fashion. Variables will be analyzed to determine whether the criteria for the trial conduct are met. This will include a description of patients who did not meet all the eligibility criteria, an assessment of protocol violations, study medication accountability and other data that study impact on the general conduct of the trial.

Baseline characteristics will be summarized for all enrolled patients. Patients who died or withdrew before treatment started or did not complete the required safety observations will be described and evaluated separately.

Treatment administration will be described for all cycles. Dose administration, dose modifications or delays and the duration of therapy will be described.

Documenting anti-tumor activity is the primary objective of this trial. Patients must receive at least one cycle of the trial medication and have had at least one post baseline RECIST assessment (or have progressed prior to this assessment) to be evaluable for response. Best overall response and overall Disease control rate (CR+PR+SD) achieved by each patient while on trial and the time to progression will be presented in the data listings.

Progression free survival (PFS) will be calculated from trial entry until the time of documented disease progression or death (whichever occurs first). Overall survival (OS) will be calculated from trial entry until the time of documented death. Patients who are alive and event free or lost to follow up at the time of analysis will be censored at the time the patient was last known to be alive and progression free. A Kaplan Meier graph and median PFS will be presented overall and by cohort.

All estimates of rates (e.g. Disease Control Rate and toxicity) will be presented with corresponding 95% exact confidence intervals. For Disease Control Rates, the method of Atkinson and Brown will be used to allow for the two-stage design.

10.7 Evaluation of Safety

Safety data will be collected from the date of written consent. Safety variables will be summarized by descriptive statistics. Laboratory variables will be described using the National Cancer Institute (NCI) Common Terminology Criteria for Adverse Events (CTCAE) Version 5.0.

Adverse events (AEs) will be reported for each dose level and presented as tables of frequency of AEs by body system and by worse severity grade observed. AE term start and end date, severity captured by CTCAE Version 5.0, seriousness, outcome, action taken regarding study drugs and relationship to each study drug will also be reported. The summary tables will also discuss AEs leading to death and AEs leading to study drug(s) discontinuation. Tables should indicate related and unrelated events. Laboratory data will be presented by dose level at each observation time. Values outside normal limits will be identified and summarized by frequency distribution.

10.8 Pharmacodynamics

Tumor biopsies for PD analysis will be collected from patients. Tumor biopsies will be collected at baseline (within 28 days before starting treatment), on-treatment (1-24 hours after taking the combination (VS-6766 and Defactinib) during Cycle 1 or Cycle 2), and upon progression as long as the biopsy of the tumor is feasible. Circulating cell free DNA will be collected prior to, before starting Cycle 2, and upon progression.

The PD data will be presented by listings and summary statistics

11 Source Documents and Access to Source Data/Documents

Source data is defined as all information in original records and certified copies of original records of clinical findings, observations, or other activities in a clinical trial necessary for

the reconstruction and evaluation of the trial. Source data are contained in source documents (original records or certified copies).

All data collected in the eCRF must be verifiable by the source data. The electronic data capture system for this trial will be Recap. Therefore, it is the Investigator's responsibility to ensure that both he/she and his/her study team records all relevant data in the medical records. The Investigator must allow the SKCC monitor direct access to relevant source documentation for verification of data entered into the eCRF, taking into account data protection regulations. Entries in the eCRF will be compared with patients' medical records and the verification will be documented on the source data verification (SDV) form and the monitoring report.

The patients' medical records, and other relevant data, may also be reviewed by appropriate qualified personnel independent from the study site(s) appointed to audit the trial, and by regulatory authorities. Details will remain confidential and patients' names will not be recorded outside the hospital.

Study staff will maintain appropriate medical and research records for this study, in compliance with ICH E6, and regulatory and institutional requirements for the protection of confidentiality of participant information. Study staff will permit authorized representatives of SKCC and regulatory agencies to examine (and when required by applicable law, to copy) research records for the purposes of quality assurance reviews, audits, and evaluation of the study safety, progress and data validity.

The study data will also be shared with University of San Diego and Verastem Oncology for the development of future collaborative activities. In that case, identifiable data on individual patients will be completely removed before sharing study data.

12 Quality Control and Quality Assurance

Quality Management is the overall process of establishing and ensuring the quality of processes, data, and documentation associated with clinical research activities. It encompasses both quality control (QC), and quality assurance (QA) activities. All sites conducting research under the sponsorship of the SKCC are required to have a plan in place for assuring the quality of the research being conducted.

Data will be evaluated for compliance with the protocol and for accuracy in relation to source documents by the KCC medical monitor. The Principal Investigator will ensure that the KCC medical monitor or other compliance or quality assurance reviewer is given access to all study-related documents and study related facilities (e.g. pharmacy, diagnostic laboratory, etc.), and has adequate space to conduct the monitoring visit.

All documents including eCRFs, clinic notes, product accountability records, specimen tracking logs, will be reviewed by the KCC medical monitor every 3 months or as required by the institutional policy.

Quality assurance issues (correcting procedures that are not in compliance with protocol) and quality control issues (correcting errors in data entry) will be addressed by the Principal Investigator. The summary of protocol deviation and violation will be included in the KCC investigator's reports to the TJU IRBs as part of its annual progress report.

Clinical trial staff will be trained in-person on the protocol study design, schedule, and data handling by the PI. The PI will track the training of all staff.

13 Ethics/Protection of Human Participants

13.1 Ethical Standard

This study is to be conducted according to US and international standards of Good Clinical Practice (FDA Title 21 part 312 and International Conference on Harmonization guidelines), applicable government regulations and Institutional research policies and procedures. The study will be conducted in accordance with the Declaration of Helsinki and with rules and regulations in accord with the U.S. Office of Protection from Research Risks (OPRR).

13.2 Institutional Review Board

The study will be reviewed and approved by a duly constituted IRB before patients are screened for entry. The investigator will ensure that all aspects of the IRB review are conducted in accordance with current institutional, local, and national regulations. Amendments to the protocol will be subject to the same requirements as the original protocol. The Investigator will submit all periodic reports and updates that the IRB may require, including any final close out reports. The Investigator will inform the IRB of any reportable adverse events as required by local regulations.

13.3 Informed Consent Process

Informed consent is a process that is initiated prior to the individual agreeing to participate in the study and continues throughout study participation. Extensive discussion of risks and possible benefits of study participation will be provided to participants and their families, if applicable. A consent form describing in detail the study procedures and risks will be given to the participant. Consent forms will be IRB-approved, and the participant is required to read and review the document or have the document read to him or her. The investigator or designee will explain the research

study to the participant and answer any questions that may arise. The participant will sign the informed consent document prior to any study-related assessments or procedures. Participants will be given the opportunity to discuss the study with their surrogates or think about it prior to agreeing to participate. They may withdraw consent at any time throughout the course of the study. A copy of the signed informed consent document will be given to participants for their records. The rights and welfare of the participants will be protected by emphasizing to them that the quality of their clinical care will not be adversely affected if they decline to participate in this study. The consent process will be documented in the clinical or research record.

13.4 Exclusion of Women, Minorities, and Children (Special Populations)

Males or females younger than 18 years of age will be excluded (children). Individuals of any gender or racial/ethnic group may participate.

13.5 Participant Confidentiality

Information about study subjects will be kept confidential and managed according to the requirements of the Health Insurance Portability and Accountability Act of 1996 (HIPAA). Those regulations require a signed subject authorization (may be part of Informed Consent Form) informing the subject of the following:

- What protected health information (PHI) will be collected from subjects in this study
- Who will have access to that information and why
- Who will use or disclose that information
- The rights of a research subject to revoke their authorization for use of their PHI.

In documents or reports or on biological samples (i.e. blood, tissue) that may be viewed by those other than study staff, patients will be identified only by their initials or a coded study number. No other PHI will be included in these documents or reports. Study results by be published but will contain no personally identifying information about study subjects.

In the event that a subject revokes authorization to collect or use PHI, the investigator, by regulation, retains the ability to use all information collected prior to the revocation of subject authorization. For subjects that have revoked authorization to collect or use PHI, attempts should be made to obtain permission to collect at least vital status (i.e. that the subject is alive) at the end of their scheduled study period.

13.6 Future Use of Stored Specimens and Other Identifiable Data

Tumor biopsies and PD blood samples will be collected, analyzed, and stored at the laboratories of the Investigators listed in the protocol. Please refer to the Study Laboratory Standard Operating Procedures for handling, storage and shipment of samples. The informed consent will specify whether the patient agrees to the future use of his/her specimens. If patients agree, residual biopsy specimen and blood samples will be stored at the protocol laboratories after the study is complete and used for unrelated research. In that case, patient information will be de-identified on the samples. No genetic testing related to inheritable non-cancer disease will be performed on the tumor biopsies or blood samples.

14 Data Handling and Record Keeping

The investigators are responsible for ensuring the accuracy, completeness, legibility, and timeliness of the data reported. All source documents must be completed in a neat, legible manner to ensure accurate interpretation of data. The investigators will maintain adequate case histories of study participants, including accurate case report forms (CRFs), and source documentation.

14.1 Data Management Responsibilities

Data collection and accurate documentation are the responsibility of the study staff under the supervision of the investigator. All source documents and laboratory reports must be reviewed by the study team and data entry staff, who will ensure that they are accurate and complete. Unanticipated problems and adverse events must be reviewed by the investigator or designee.

14.2 Data Capture Methods

Electronic CRFs (eCRFs) will be used to collect the data. These eCRF’s will be in the RedCap Electronic Data Capture system. The Investigator is responsible for ensuring the accuracy, completeness, clarity and timeliness of the data reported in the eCRFs.

Only the Investigator and those personnel who have signed the Study Team Responsibilities Signature Log/Delegation Log have been authorized by the Investigator should enter or change data in the eCRFs. All protocol required investigations must be reported in the eCRF. The Investigators must retain all source data (contained in original reports, traces and images from protocol investigations) for future reference.

14.3 Types of Data

Types of data to be collected for this trial will include demographic information, medical history, laboratory results (blood and urine), pregnancy test results, tumor biopsy results, pharmacodynamic analysis data, physical examination, vital signs, ECG data, ophthalmologic examination data, radiological disease assessments (CT and MRI), circulating free DNA analysis data, concomitant treatments, and adverse event assessments.

14.4 Study Records Retention

During the clinical trial and after trial closure the Investigator must maintain adequate and accurate records to enable both the conduct of a clinical trial and the quality of the data produced to be evaluated and verified. These essential, including source documents such as scans, trial related documents and copies of the eCRFs, associated audit trail and serious adverse event (SAE) report forms, shall show whether the Investigator has complied with the principles and guidelines of Good Clinical Practice (GCP).

All essential documents required to be held by the Investigator must be stored in such a way that ensures that they are readily available, upon request, to the Regulatory Agencies, for the minimum period of 2 years after completion of the study in accordance with the FDA guideline [21CFR 812.140(d), 12 CRF 312.62].

The medical files of trial subjects shall be retained in accordance with the FDA guideline and in accordance with the maximum period of time permitted by the hospital, institution or private practice.

14.5 Protocol Deviations

A protocol deviation is any noncompliance with the clinical study protocol, Good Clinical Practice, or Manual of Procedures requirements. The noncompliance may be on the part of the participant, the investigator, or study staff. As a result of deviations, corrective actions are to be developed by the study staff and implemented promptly.

All deviations from the protocol must be addressed in study participant source documents and promptly reported to the IRB and other regulatory bodies according to their requirements.

Eligibility waivers are strictly prohibited. Protocol deviations will be recorded, will be considered at the time of study analysis and where appropriate, will be documented in the clinical study report.

Amendments to the protocol may only be made with the approval of the IRB. Amendments will be subject to review by the sponsor. Non-substantial amendments do not require FDA approval. Urgent safety measures may be implemented immediately where necessary but the sponsor should be informed immediately (see section 8.4).

15 Study Finances

15.1 Funding Source

The study will be funded by Verastem Oncology, Inc, as well as institutional funds (Metastatic Uveal Melanoma Research Fund) at Thomas Jefferson University.

15.2 Conflict of Interest

Any investigator who has a conflict of interest with this study (patent ownership, royalties, or financial gain greater than the minimum allowable by their institution, etc.) must have the conflict reviewed by a properly constituted Conflict of Interest Committee with a Committee-sanctioned conflict management plan that has been reviewed and approved by the study sponsor prior to participation in this study. All Jefferson University Investigators will follow the TJU Conflicts of Interest Policy for Employees (107.03).

15.3 Participant Stipends or Payments

There is no compensation for participation in this study.

16 Publication and Data Sharing Policy

This study will comply with the NIH Public Access Policy, which ensures that the public has access to the published results of NIH funded research. It requires scientists to submit final peer-reviewed journal manuscripts that arise from NIH funds to the digital archive PubMed Central upon acceptance for publication.

The International Committee of Medical Journal Editors (ICMJE) member journals have adopted a clinical trials registration policy as a condition for publication. The ICMJE defines a clinical trial as any research project that prospectively assigns human participants to intervention or concurrent comparison or control groups to study the cause-and-effect relationship between a medical intervention and a health outcome. Medical interventions include drugs, surgical procedures, devices, behavioral treatments, process-of-care changes, and the like. Health outcomes include any biomedical or health-

related measures obtained in patients or participants, including pharmacokinetic measures and adverse events. The ICMJE policy requires that all clinical trials be registered in a public trials registry such as ClinicalTrials.gov, which is sponsored by the National Library of Medicine. Other biomedical journals are considering adopting similar policies. The ICMJE does not review specific studies to determine whether registration is necessary; instead, the committee recommends that researchers who have questions about the need to register err on the side of registration or consult the editorial office of the journal in which they wish to publish.

U.S. Public Law 110-85 (Food and Drug Administration Amendments Act of 2007 or FDAAA), Title VIII, Section 801 mandates that a "responsible party" (i.e., the sponsor or designated principal investigator) register and report results of certain "applicable clinical trials:"

Trials of Drugs and Biologics: Controlled, clinical investigations, other than Phase I investigations, of a product subject to FDA regulation;

Trials of Devices: Controlled trials with health outcomes of a product subject to FDA regulation (other than small feasibility studies) and pediatric postmarket surveillance studies.

NIH grantees must take specific steps to ensure compliance with NIH implementation of FDAAA.

17 Literature References

1. Singh AD, Turell ME, Topham AK. Uveal melanoma: trends in incidence, treatment, and survival. Ophthalmology. 2011 Sep;118(9):1881-5.
2. Diener-West M, Reynolds SM, Agugliaro DJ, Caldwell R, Cumming K, Earle JD, et al. Development of metastatic disease after enrollment in the COMS trials for treatment of choroidal melanoma: Collaborative Ocular Melanoma Study Group Report No. 26. Arch Ophthalmol. 2005 Dec;123(12):1639-43.
3. Kujala E, Makitie T, Kivela T. Very long-term prognosis of patients with malignant uveal melanoma. Invest Ophthalmol Vis Sci. 2003 Nov;44(11):4651-9.
4. Economou MA, All-Ericsson C, Bykov V, Girnita L, Bartolazzi A, Larsson O, et al. Receptors for the liver synthesized growth factors IGF-1 and HGF/SF in uveal melanoma: intercorrelation and prognostic implications. Acta Ophthalmol. 2008 Nov;86:20-5.
5. Bedikian AY. Metastatic uveal melanoma therapy: current options. Int Ophthalmol Clin. 2006 Winter;46(1):151-66.
6. Albert DM, Ryan LM, Borden EC. Metastatic ocular and cutaneous melanoma: a comparison of patient characteristics and prognosis. Archives of ophthalmology. 1996 Jan;114(1):107-8.
7. Bedikian AY, Legha SS, Mavligit G, Carrasco CH, Khorana S, Plager C, et al. Treatment of uveal melanoma metastatic to the liver: a review of the M. D. Anderson Cancer Center experience and prognostic factors. Cancer. 1995 Nov 1;76(9):1665-70.
8. Kath R, Hayungs J, Bornfeld N, Sauerwein W, Hoffken K, Seeber S. Prognosis and treatment of disseminated uveal melanoma. Cancer. 1993 Oct 1;72(7):2219-23.
9. Gragoudas ES, Egan KM, Seddon JM, Glynn RJ, Walsh SM, Finn SM, et al. Survival of patients with metastases from uveal melanoma. Ophthalmol. 1991 Mar;98(3):383-9.
10. Krauthammer M, Kong Y, Ha BH, Evans P, Bacchiocchi A, McCusker JP, et al. Exome sequencing identifies recurrent somatic RAC1 mutations in melanoma. Nat Genet. 2012 Sep;44(9):1006-14.
11. Furney SJ, Pedersen M, Gentien D, Dumont AG, Rapinat A, Desjardins L, et al. SF3B1 mutations are associated with alternative splicing in uveal melanoma. Cancer Discov. 2013 Oct;3(10):1122-9.
12. Yang J, Manson DK, Marr BP, Carvajal RD. Treatment of uveal melanoma: where are we now? Ther Adv Med Oncol. 2018;10:1758834018757175.
13. Johnson DB, Daniels AB. Continued Poor Survival in Metastatic Uveal Melanoma: Implications for Molecular Prognostication, Surveillance Imaging, Adjuvant Therapy, and Clinical Trials. JAMA Ophthalmol. 2018 Sep 1;136(9):986-8.
14. Algazi AP, Tsai KK, Shoushtari AN, Munhoz RR, Eroglu Z, Piulats JM, et al. Clinical outcomes in metastatic uveal melanoma treated with PD-1 and PD-L1 antibodies. Cancer. 2016 Nov 15;122(21):3344-53.
15. Schank TE, Hassel JC. Immunotherapies for the Treatment of Uveal Melanoma-History and Future. Cancers (Basel). 2019 Jul 24;11(8).

16. Bol KF, Ellebaek E, Hoejberg L, Bagger MM, Larsen MS, Klausen TW, et al. Real-World Impact of Immune Checkpoint Inhibitors in Metastatic Uveal Melanoma. Cancers (Basel). 2019 Oct 3;11(10).
17. Heppt MV, Amaral T, Kahler KC, Heinzerling L, Hassel JC, Meissner M, et al. Combined immune checkpoint blockade for metastatic uveal melanoma: a retrospective, multi-center study. J Immunother Cancer. 2019 Nov 13;7(1):299.
18. Harbour JW. The genetics of uveal melanoma: an emerging framework for targeted therapy. Pigment Cell Melanoma Res. 2012 Mar;25(2):171-81.
19. Van Raamsdonk CD, Bezrookove V, Green G, Bauer J, Gaugler L, O'Brien JM, et al. Frequent somatic mutations of GNAQ in uveal melanoma and blue naevi. Nature. 2009 Jan 29;457(7229):599-602.
20. Van Raamsdonk CD, Griewank KG, Crosby MB, Garrido MC, Vemula S, Wiesner T, et al. Mutations in GNA11 in uveal melanoma. N Engl J Med. 2010;363(23):2191-9.
21. Onken MD, Worley LA, Long MD, Duan S, Council ML, Bowcock AM, et al. Oncogenic mutations in GNAQ occur early in uveal melanoma. Invest Opthal Vis Sci. 2008 Dec;49(12):5230-4.
22. Vaque JP, Dorsam RT, Feng X, Iglesias-Bartolome R, Forsthoefel DJ, Chen Q, et al. A genome-wide RNAi screen reveals a Trio-regulated Rho GTPase circuitry transducing mitogenic signals initiated by G protein-coupled receptors. Mol Cell. 2013 Jan 10;49(1):94-108.
23. Khalili JS, Yu X, Wang J, Hayes BC, Davies MA, Lizee G, et al. Combination small molecule MEK and PI3K inhibition enhances uveal melanoma cell death in a mutant GNAQ- and GNA11-dependent manner. Clin Cancer Res. 2012 Aug 15;18(16):4345-55.
24. Carvajal RD, Sosman JA, Quevedo JF, Milhem MM, Joshua AM, Kudchadkar RR, et al. Effect of selumetinib vs chemotherapy on progression-free survival in uveal melanoma: a randomized clinical trial. Jama. 2014 Jun 18;311(23):2397-405.
25. Carvajal RD, Schwartz GK, Mann H, Smith I, Nathan PD. Study design and rationale for a randomised, placebo-controlled, double-blind study to assess the efficacy of selumetinib (AZD6244; ARRY-142886) in combination with dacarbazine in patients with metastatic uveal melanoma (SUMIT). BMC Cancer. 2015;15:467.
26. Feng X, Degese MS, Iglesias-Bartolome R, Vaque JP, Molinolo AA, Rodrigues M, et al. Hippo-independent activation of YAP by the GNAQ uveal melanoma oncogene through a trio-regulated rho GTPase signaling circuitry. Cancer Cell. 2014 Jun 16;25(6):831-45.
27. Feng X, Arang N, Rigiracciolo DC, Lee JS, Yeerna H, Wang Z, et al. A Platform of Synthetic Lethal Gene Interaction Networks Reveals that the GNAQ Uveal Melanoma Oncogene Controls the Hippo Pathway through FAK. Cancer Cell. 2019 Mar 18;35(3):457-72 e5.
28. Sulzmaier FJ, Jean C, Schlaepfer DD. FAK in cancer: mechanistic findings and clinical applications. Nat Rev Cancer. 2014 Sep;14(9):598-610.
29. Serrels A, Lund T, Serrels B, Byron A, McPherson RC, von Kriegsheim A, et al. Nuclear FAK controls chemokine transcription, Tregs, and evasion of anti-tumor immunity. Cell. 2015 Sep 24;163(1):160-73.

30. Martinez-Garcia M, Banerji U, Albanell J, Bahleda R, Dolly S, Kraeber-Bodere F, et al. First-in-human, phase I dose-escalation study of the safety, pharmacokinetics, and pharmacodynamics of RO5126766, a first-in-class dual MEK/RAF inhibitor in patients with solid tumors. Clin Cancer Res. 2012 Sep 1;18(17):4806-19.
31. Honda K, Yamamoto N, Nokihara H, Tamura Y, Asahina H, Yamada Y, et al. Phase I and pharmacokinetic/pharmacodynamic study of RO5126766, a first-in-class dual Raf/MEK inhibitor, in Japanese patients with advanced solid tumors. Cancer Chemother Pharmacol. 2013 Sep;72(3):577-84.
32. Lynch TJ, Jr., Kim ES, Eaby B, Garey J, West DP, Lacouture ME. Epidermal growth factor receptor inhibitor-associated cutaneous toxicities: an evolving paradigm in clinical management. Oncologist. 2007 May;12(5):610-21.
33. Kaliki S, Shields CL. Uveal melanoma: relatively rare but deadly cancer. Eye (Lond). 2017 Feb;31(2):241-57.
34. van Nimwegen MJ, van de Water B. Focal adhesion kinase: a potential target in cancer therapy. Biochem Pharmacol. 2007 Mar 1;73(5):597-609.
35. Siesser PM, Hanks SK. The signaling and biological implications of FAK overexpression in cancer. Clin Cancer Res. 2006 Jun 1;12(11 Pt 1):3233-7.
36. Weis SM, Lim ST, Lutu-Fuga KM, Barnes LA, Chen XL, Gothert JR, et al. Compensatory role for Pyk2 during angiogenesis in adult mice lacking endothelial cell FAK. J Cell Biol. 2008 Apr 7;181(1):43-50.
37. Gerber DE, Camidge DR, Morgensztern D, Cetnar J, Kelly RJ, Ramalingam SS, et al. Phase 2 study of the focal adhesion kinase inhibitor defactinib (VS-6063) in previously treated advanced KRAS mutant non-small cell lung cancer. Lung Cancer. 2020 Jan;139:60-7.
38. Flaherty KT, Puzanov I, Kim KB, Ribas A, McArthur GA, Sosman JA, et al. Inhibition of mutated, activated BRAF in metastatic melanoma. N Engl J Med. 2010 Aug 26;363(9):809-19.
39. Banerji U, Camidge DR, Verheul HM, Agarwal R, Sarker D, Kaye SB, et al. The first-in-human study of the hydrogen sulfate (Hyd-sulfate) capsule of the MEK1/2 inhibitor AZD6244 (ARRY-142886): a phase I open-label multicenter trial in patients with advanced cancer. Clin Cancer Res. 2010 Mar 1;16(5):1613-23.
40. LoRusso PM, Krishnamurthi SS, Rinehart JJ, Nabell LM, Malburg L, Chapman PB, et al. Phase I pharmacokinetic and pharmacodynamic study of the oral MAPK/ERK kinase inhibitor PD-0325901 in patients with advanced cancers. Clin Cancer Res. 2010 Mar 15;16(6):1924-37.
41. Ishii N, Harada N, Joseph EW, Ohara K, Miura T, Sakamoto H, et al. Enhanced inhibition of ERK signaling by a novel allosteric MEK inhibitor, CH5126766, that suppresses feedback reactivation of RAF activity. Cancer Res. 2013 Jul 1;73(13):4050-60.
42. Eisenhauer EA, Therasse P, Bogaerts J, Schwartz LH, Sargent D, Ford R, et al. New response evaluation criteria in solid tumours: revised RECIST guideline (version 1.1). Eur J Cancer. 2009 Jan;45(2):228-47.
43. Schwartz LH, Litiere S, de Vries E, Ford R, Gwyther S, Mandrekar S, et al. RECIST 1.1-Update and clarification: From the RECIST committee. Eur J Cancer. 2016 Jul;62:132-7.

44. Simon R. Optimal two-stage designs for phase II clinical trials. Control Clin Trials. 1989 Mar;10(1):1-10.
45. Clinical Trial Facilitation Group (CTFG). (2014). Recommendations related to contraception and pregnancy testing in clinical trials. 2014 [06/22/20]; Available from: https://www.hma.eu/fileadmin/dateien/Human_Medicines/01-About_HMA/Working_Groups/CTFG/2014_09_HMA_CTFG_Contraception.pdf.

Appendices

The following documents are officially affiliated with the protocol and will be submitted to the IRB as a part of the protocol. As such, changes to these items require a protocol amendment.

APPENDIX A: SCHEDULE OF EVENTS

APPENDIX B: MEASUREMENT OF DISEASE

APPENDIX C: RECOMMENDATIONS RELATED TO CONTRACEPTION AND PREGNANCY TESTING IN CLINICAL TRIALS

APPENDIX D: PROTOCOL HISTORY

APPENDIX A: SCHEDULE OF EVENTS

1 cycle = 28 days*

Observation/Investigation	Baseline/Pre-study		Cycles 1 & 2**				Cycles 3 & 4 **	Cycle 5 onwards **	Off study and Follow-up	
	Within 4 weeks prior to first dose	Within 2 weeks prior to first dose	Day 1	Day 8	Day 15	Day 22	Day 1	Day 1	Off study: within 28 days after last dose	Follow-up: monthly (a)
Written informed consent	X									
Demographics & medical history	X									
Adverse event evaluation	X		Continually review						X	Until resolution (a)
Concomitant treatments	X		Continually review						X	
Echocardiogram or MUGA	X									
Radiological (CT, MRI) disease assessment (b)	X						X C3D1	X (b)	X	
Clinical disease assessment (b)		X					X C3D1	X (b)	X	
Ophthalmological examination (c)	X		X C2D1				X C3D1	X C5D1(c)	X	
Triplicate electrocardiogram (ECG)		X	X C2D1						X	
Pregnancy test (d)		X	X				X	X	X	
Physical examination		X	X				X	X	X	
ECOG performance status and Vital Signs		X	X				X	X	X	
Urinalysis		X	X				X	X	X	
Laboratory tests: Hematology and biochemistry (e)		X	X	X	X	X	X	X	X	To resolution of drug-related lab AEs

Coagulation (f)	X		X						X	
Tumor biopsy (f)	X		X						X	
Blood for pharmacodynamic (PD) analysis (g)		X					X C3D1		X	
Blood for ctDNA analysis		X					X (h)	X (h)	X	
VS-6766 administration			X	X	X		X	X		
Defactinib administration			X	X	X		X	X		

<u>* There will be visit windows of +/-2 days in Cycle 1, or on Day 1 of any cycle. For interim safety visits (Cycle 2 onwards, Day 8, 15 or 22) a window of +/- 2 days is acceptable. The cycle length must always remain 28 +/- 2 days.</u>

<u>** Patients will be followed up weekly during Cycles 1 and 2, then seen on Day 1 of each cycle thereafter. These assessments can be performed within the 24 hours prior to dosing.</u>

a) Monthly follow-up required for all AEs regardless of causality present at time the patient comes off the trial. Monthly follow-up to continue until resolution, to baseline, stabilization or patient starts another anti-cancer treatment.
b) Disease assessment must be performed at baseline and repeated every 2 cycle(s)/8 weeks of continuous treatment and at off study visit (unless assessment has been performed within previous 4 weeks). For patients who continue on treatment after 6 cycles frequency may be reduced. Clinical disease assessment and tumor markers should be assessed if applicable.
c) Ophthalmological examination must be performed at baseline (within 28 days prior to first dose), end of Cycle 1 (between Cycle 1 Day 22 and pre-dose Cycle 2 Day 1), end of Cycle 2 (between Cycle 2 Day 22 and pre-dose Cycle 3 Day 1), end of Cycle 4 (between Cycle 4 Day 22 and pre-dose Cycle 5 Day 1) and every 3 cycles thereafter and within 28 days after the last dose of study drugs. Result must be available prior to dosing (Section 6.1.2).
d) Pregnancy test only required if the patient is a woman of child bearing potential.
e) In the event of a Grade 4 neutropenia or Grade 4 thrombocytopenia, a full blood count must be performed at least on Day 5 after the onset of the event to determine if a dose limiting toxicity has occurred. Continue close monitoring until resolution to Grade 3 or less.
f) Three tumor biopsy samples will be taken at the following time points: at baseline (within 4 weeks), after cycle 1 or 2, and within 28 days after the last dose of the study medications. Coagulation should be tested prior to each biopsy as per institutional standard practice (at minimum within 7 days prior to procedure). On-treatment biopsy should be performed 2-6 hours after VS-6766 dose during day 1-21. Post-progression biopsy (is possible) should also be 2-6 hours after VS-6766 dose.
g) Blood sample for PD analysis will be taken at the following time points: at baseline (within 14 days of first dose), on C3D1 prior to administration of study medication, and within 28 days after the last dose of the study medications
h) Blood for ctDNA analysis will be taken at the end of every 2 cycles for up to 6 cycles, i.e. C3D1 & C5D1 (Section 6.6.2.2).

APPENDIX B: MEASUREMENT OF DISEASE (42, 43)

Eisenhaur et al. (2009). New response evaluation criteria in solid tumours: Revised RECIST Guideline (version 1.1). *Eur J Cancer*, 45(2), 228-247. doi:10.1016/j.ejca.2008.10.026. (42)

Schwartz et al. (2016). RECIST 1.1-Update and clarification: From the RECIST committee. *Eur J Cancer, 62*, 132-137. doi:10.1016/j.ejca.2016.03.081. (43)

APPENDIX C: RECOMMENDATIONS RELATED TO CONTRACEPTION AND PREGNANCY TESTING IN CLINICAL TRIALS (45)

Clinical Trial Facilitation Group (CTFG). (2014). Recommendations related to contraception and pregnancy testing in clinical trials. Retrieved from https://www.hma.eu/fileadmin/dateien/Human_Medicines/01-About_HMA/Working_Groups/CTFG/2014_09_HMA_CTFG_Contraception.pdf. (45)

APPENDIX D: PROTOCOL HISTORY

1. **First Version (06/12/2020)**; written by Dr. Rino Seedor and modified by Dr. Takami Sato and was approved by TJU IRB as part of New Study Submission.

2. **Second Version (07/07/2021)**; modified by Dr. Rino Seedor. Revisions include:
   - Investigator and research staff changes; **Title page, Signature page**
   - Updated IND number; **Title page**
   - Clarification of radiologic disease assessments; **Sec. 6.1.2**
   - Modified AE/SAE reviewer; **Sec. 6.2**
   - Clarification of ophthalmological examination schedule; **Sec. 6.2**
   - Modified schedule for blood draws for PD testing; **Schematic of study design, Sec. 6.1.3, 6.2, 6.6.2.1, Appendix A**
   - Modified schedule for blood draw for ctDNA testing; **Schematic of study design, Sec. 6.1.3, 6.6.2.2, Appendix A**
   - Modified tumor biopsy schedule and procedure; **Schematic of study design, Sec. 6.2, 6.6.2.1, Appendix A**
   - Clarification of coagulation testing schedule; **Sec. 6.1.2, 6.1.3, 6.6.1**
   - Added External Laboratory to approved laboratories; **Sec. 6.6.2.1 and 6.6.2.2**
   - Removal of BAP1 testing; **Sec. 6.6.2.2**

3. **Third Version (06/2022)**
   - Investigator and research staff changes; **Title page, Signature page**
   - Modified follow up duration; **Study Summary, Sec. 3.4, 3.6.1, 6.4**
   - Modified tumor biopsy timing; **Sec. 6.2.1, Appendix A**
