# Supplementary material for: A Phase II Trial of Defactinib Combined with Avutometinib in Patients with Metastatic Uveal Melanoma
Source: Cancers (Basel). 2026 Jul 11;18(14):2232. doi: 10.3390/cancers18142232 (PMC13407007; doi:10.3390/cancers18142232)
Supplement: Supplementary file 1 [file cancers-18-02232-s001.zip › Supplementary Figures.pdf]

Supplemental Figure S1. Immunohistochemical staining for the ALDH1A3 protein in matched pre- and post-treatment PDX specimens from MUM patients.

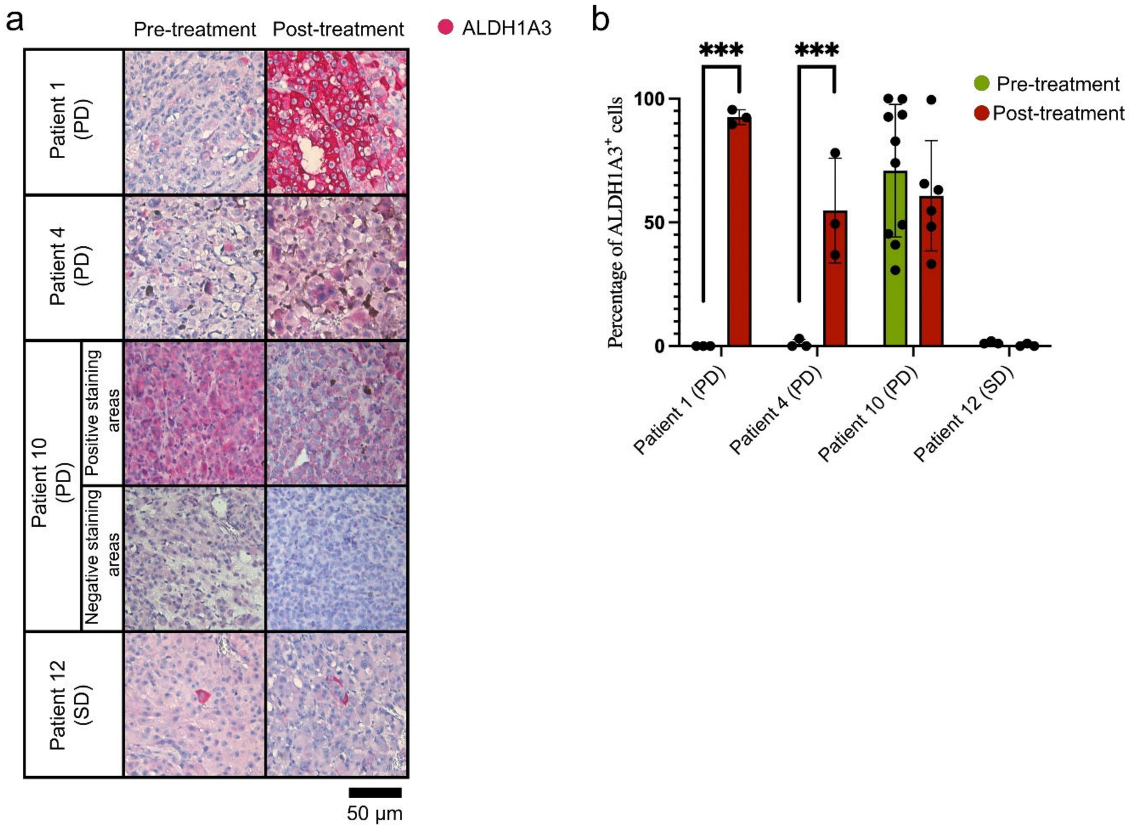

(a) ALDH1A3 protein was detected by immunohistochemistry using an alkaline phosphatase-conjugated secondary antibody. Representative 200× fields are shown; scale bar, 50 μm. (b) Quantification of ALDH1A3-positive cells in 4 matched pre- and post-treatment PDX specimens derived from MUM patients. Green bars indicate pre-treatment samples, and red bars indicate post-treatment samples. Individual dots represent quantified tumor fields, and bars show the mean ± SD. For quantification, ALDH1A3-positive cells were counted in 3–10 non-overlapping viable tumor fields per PDX tumor at 200× magnification. Three fields were analyzed when staining was homogeneous, whereas additional fields, up to 10 total, were evaluated when staining was heterogeneous or equivocal. ***$p < 0.001$.

Supplemental Figure S2. Descriptive boxplot of TPM-normalized expression of selected pathway-related genes in an independent institutional cohort of metastatic uveal melanoma biopsy specimens.

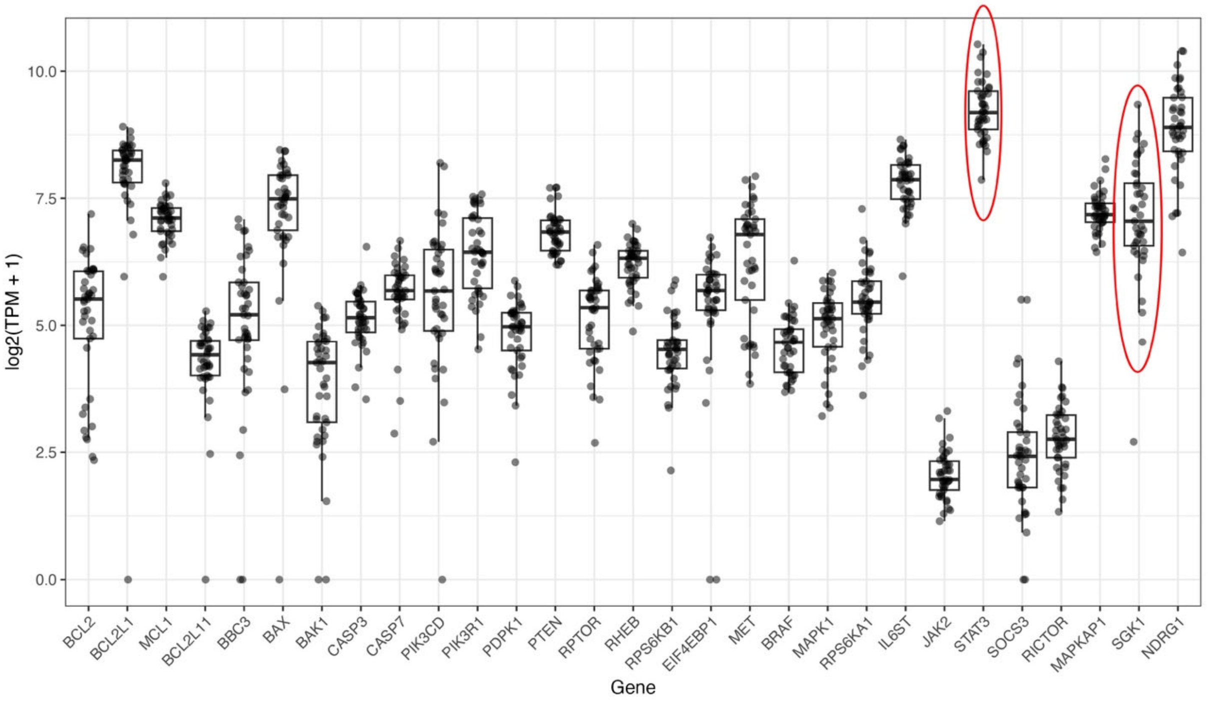

Boxplots show log2(TPM + 1) mRNA expression values for selected pathway-related genes in metastatic biopsy specimens from our institutional MUM omics database (*n* = 41). These specimens were obtained from patients who were not included in the current clinical trial and served as an independent descriptive reference cohort. Individual points represent samples. Red circles highlight selected pathway-related genes of interest, STAT3 (JAK/STAT pathway) and SGK1 (SGK/mTORC2 pathway).

Supplemental Figure S3. Descriptive heatmap of pathway-related protein and phosphoprotein abundance measured by RPPA in metastatic biopsy specimens.

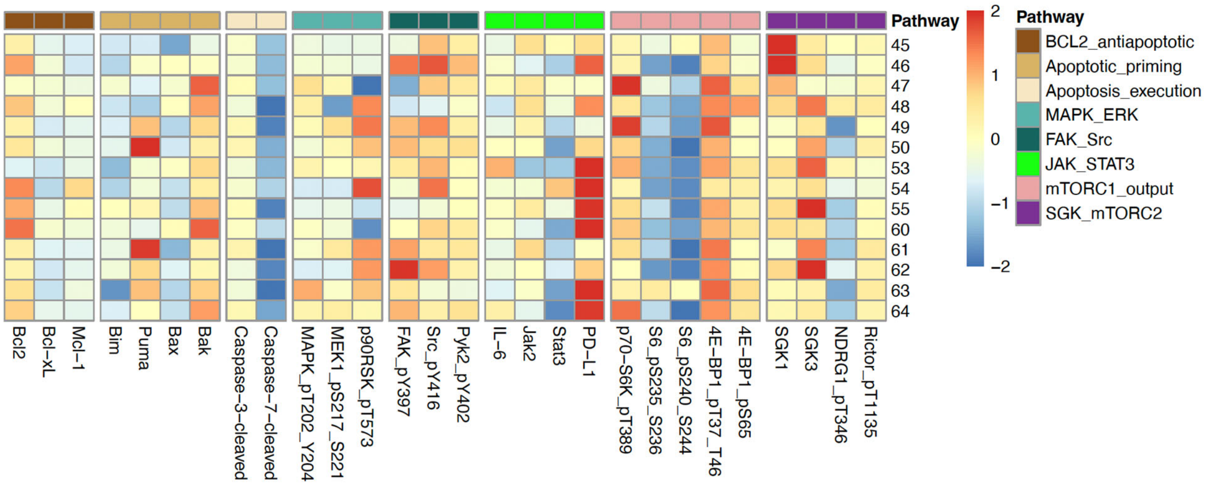

RPPA values are shown as L4 log2-normalized protein abundance, where 0 represents the cohort median, +1 indicates approximately 2-fold higher abundance, and -1 indicates approximately 2-fold lower abundance of a given protein or phosphoprotein relative to the cohort. Positive values indicate higher protein abundance and/or phosphorylation (consistent with increased pathway output), whereas negative values indicate lower abundance and/or phosphorylation compared with the typical tumor in the dataset. Antibodies were grouped by signaling module, while samples were displayed without unsupervised clustering. No statistical testing was applied to this visualization. The heatmap should be interpreted as a descriptive overview of pathway-related RPPA patterns in metastatic UM biopsy specimens.
